# Treating to target in multiple sclerosis: Do we know how to measure whether we hit it?

**DOI:** 10.1111/ene.16526

**Published:** 2024-10-24

**Authors:** Gabriel Bsteh, Nik Krajnc, Patrick Altmann, Barry Hendin, Trishna Bharadia, Sonja Jaruszowic, Fred Lublin, Jiwon Oh, Detlev Parow, Annemie Ribbens, Aoife Shields, Dirk Smeets, Eric Thouvenot, Andrew Chan, Thomas Berger

**Affiliations:** ^1^ Department of Neurology Medical University of Vienna Vienna Austria; ^2^ Comprehensive Center for Clinical Neurosciences and Mental Health Medical University of Vienna Vienna Austria; ^3^ Neurology University of Arizona Tucson Arizona USA; ^4^ Patient author Buckinghamshire UK; ^5^ Neuro Centrum Science Erbach Germany; ^6^ Corinne Goldsmith Dickinson Center for Multiple Sclerosis Icahn School of Medicine at Mount Sinai New York New York USA; ^7^ Division of Neurology, Department of Medicine, St. Michael's Hospital University of Toronto Toronto Ontario Canada; ^8^ Pharmaceutical Department Deutsche Angestellten‐Krankenkasse (DAK) Gesundheit Hamburg Germany; ^9^ icometrix Leuven Belgium; ^10^ University College Hospital London London UK; ^11^ Department of Neurology Centre hospitalier universitaire (CHU) de Nîmes Nîmes France; ^12^ Institut de Génomique Fonctionnelle University of Montpellier Montpellier France; ^13^ Department of Neurology, Inselspital Bern University Hospital and University of Bern Bern Switzerland

**Keywords:** applicability, measures, multiple sclerosis, outcome, review, treat‐to‐target, utility

## Abstract

**Background and purpose:**

The rapidly evolving landscape of effective treatment options in multiple sclerosis has led to a shift of treatment objectives towards a treat‐to‐target approach aiming to suppress disease activity below the level of detectability early during the disease. To enable treat‐to‐target, a thorough reappraisal of available outcome measures with respect to their ability in this regard is required.

**Methods:**

To that end, we conducted a comprehensive systematic literature review of more than 1000 studies using PRISMA (Preferred Reporting Items for Systematic Reviews and Meta‐Analyses) 2020 methodology focusing on underlying evidence as well as utility and implementability in clinical practice.

**Results:**

From there, we propose a set of measurable outcomes for everyday routine clinical practice as well as advanced/aspirational measurables requiring additional resources. We also outline remaining knowledge/technology gaps that need to be overcome to enable a treat‐to‐target approach.

**Conclusions:**

This work provides the basis for an evidence‐based definition of outcome targets for relevant stakeholders and regulatory authorities.

## INTRODUCTION

Multiple sclerosis (MS), an immune‐mediated chronic inflammatory demyelinating disorder of the central nervous system, represents the most frequent cause of neurological disability in young adults [1].

The rapidly evolving landscape of effective disease‐modifying treatments (DMTs) has led to a shift of treatment objectives towards aiming to suppress disease activity below the level of detectability in the sense of a treat‐to‐target approach already during the earliest stages of the disease. This concept is already common custom in other immune‐mediated inflammatory diseases such as rheumatoid arthritis and psoriasis [2, 3]. However, MS is characterized by a high degree of heterogeneity, extending from clinical course over radiological features to underlying pathology [4, 5, 6, 7]. Clinical parameters such as relapse rate and disability progression have been the mainstays of assessing treatment response in MS and are supplemented by paraclinical markers, mainly magnetic resonance imaging (MRI). Although a multitude of other surrogate markers were and are investigated regarding their capability in assessing treatment response, few (if any) have made their way to implementation into clinical routine so far.

Thus, assessing treatment response with a treat‐to‐target approach in people with MS (pwMS) might still correspond to aiming at a moving and shape‐shifting target. To advance treat‐to‐target in MS, a thorough reappraisal of available outcome measures (OMs) with respect to their ability in this regard is required.

## METHODOLOGY

The objective of this systematic literature review (SLR) was to identify current and potential measurable DMT outcome targets in MS with the specific goal of focusing on utility and implementability in clinical practice to improve quality of care in MS.

**FIGURE 1:**
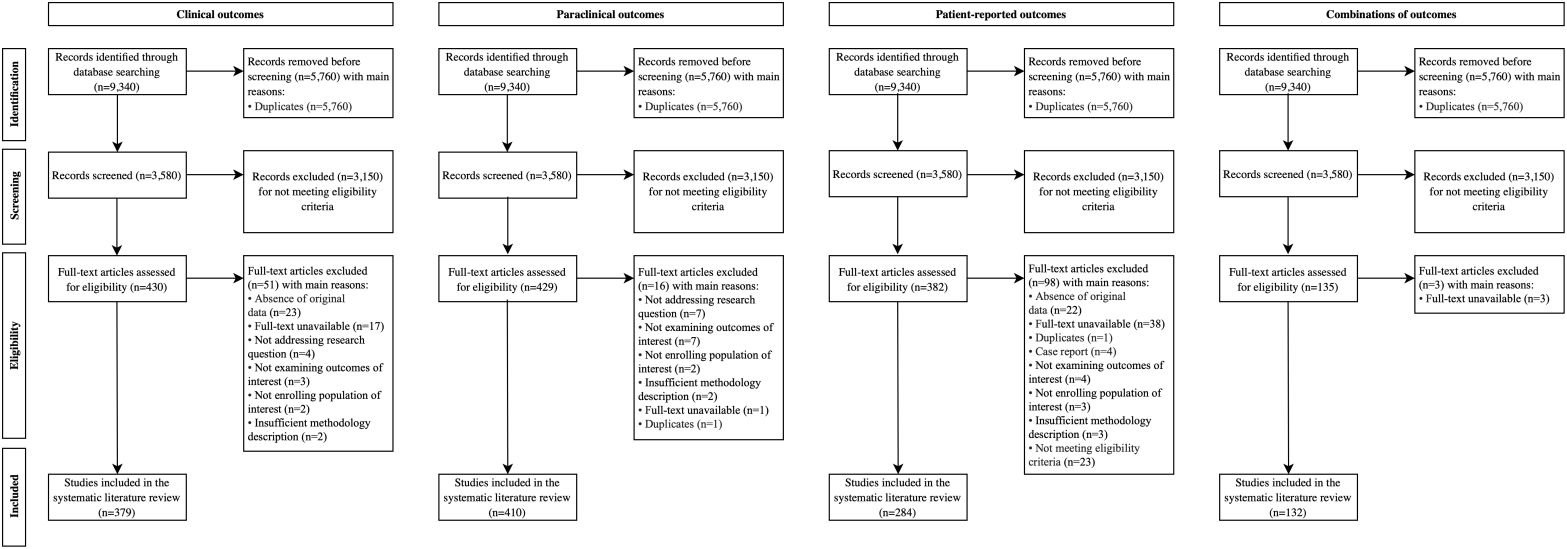
Inclusion/exclusion process.

The SLR was conducted according to the Preferred Reporting Items for Systematic Reviews and Meta‐Analyses (PRISMA) 2020 methodology [8]. The study search and selection process is described in detail in the Supplemental Methods and transparently documented in Figure 1 and a screening list (Table S1).

Briefly, the literature search was conducted in the databases of MEDLINE, Embase, and the Cochrane library with the main search terms “multiple sclerosis” and “outcome.” Eligibility criteria comprised studies conducted in persons with MS reporting any outcome viewed as a potential treatment target and published between 1 January 2017 and 31 August 2022 in English.

### Grading of evidence

The methodological quality for each outcome was graded according to the quality of evidence (QoE) using the GRADE (Grades of Recommendation, Assessment, Development, and Evaluation) tool for best‐evidence synthesis following the four‐eye principle (authors G.B./N.K./P.A. and T.Be.) [9, 10]. All studies included were systematically analyzed regarding several key criteria (study design, sample size, study population, minimal detectable change [MDC], clinically relevant change [CRC], external and ecological validity, sources of bias), separately assessed for each OM. In accordance with PRISMA 2020 methodology, each step of the grading process was transparently documented in Table S2 [8].

## RESULTS

Here, the results of the conducted SLR are narratively summarized within the framework of the four groups of OMs. The Supplemental Results section discusses those not described here.

### Objectively measurable clinical outcomes

The search process identified 430 studies, of which 379 on 80 different objectively measurable clinical outcomes were eventually included (see Figure 1). Here, we describe only those most relevant in assessing treatment response in clinical practice. Table S3 provides a detailed summary.

#### Relapses

Relapses were used as OM in 177 studies comprising 180,914 pwMS, with the majority (137) conducted within a real‐world setting (RW) and using relapses as primary outcome endpoint. The overall QoE for relapses as OM was rated as high; their clinical usefulness is well documented and can be generalized to all patients with relapsing MS (relapsing–remitting and secondary progressive ) but naturally not to primary progressive MS (PPMS). Although relapses are well applicable in RW, their usefulness is limited by their rarity as well as their low sensitivity regarding detection of disease activity, which is formally represented by a complete congruence of MDC and CRC. Another known limitation is the heterogenic use of varying definitions of what constitutes a relapse, especially regarding the differentiation between reported relapses as opposed to those confirmed by a neurologist and those requiring an increase in Expanded Disability Status Scale (EDSS). Within the identified studies, we found 10 different relapse definitions. Relapses require little time for assessment but need to be confirmed by a trained neurologist without the possibility of sourcing out to trained staff or digitalization/self‐application by the patient.

Relapses must be considered a core OM within the relapsing multiple sclerosis (RMS) population. Still, there remain knowledge gaps regarding whether a further differentiation beyond the dichotomization of a patient remaining free of relapses or not, for example, according to severity of relapses, functional system affected, or degree of relapse recovery beyond the first clinical attack, would provide additional valuable information.

#### Expanded Disability Status Scale

The second mainstay of clinical MS outcomes after relapses, EDSS was applied as OM in 246,604 pwMS and 210 studies, of whom 139 took place within RW. QoE for EDSS as OM was rated as high. Clinical usefulness is well documented but also limited by the low sensitivity for change in disability, as MDC and CRC are congruent. This is aggravated by the varying definitions of what constitutes EDSS worsening. We identified 672 different definitions of EDSS worsening depending on minimal increase, required confirmation, and fixed/roving‐baseline score. EDSS is also strongly driven by walking ability (especially between scores 4–7), mostly disregards upper‐extremity function and neuropsychological disability, and is a nonlinear metric. Regarding external validity, EDSS is generalizable to all pwMS independent of age and disease course, although there is some interrater variability (IRV) strongly depending on the level of training of raters. For ecological validity, EDSS is applicable in RW, mainly due to its familiarity within the MS community, even though it requires 10–20 min (depending on the individual level of disability) by a trained neurologist without the possibility of outsourcing to trained staff or digitalization/self‐application by the patient. Despite these limitations, EDSS is considered a core OM. Important knowledge gaps remain regarding the optimal definition of EDSS worsening, most essentially determining the required observation frequency and time for confirmation, as well as consistent and rigorous training of raters to improve IRV and comparability.

#### Timed 25 Foot Walk Test

The Timed 25 Foot Walk Test (T25FW), a measure of short‐range walking speed, was used as OM in 87 studies on 40,359 pwMS, in 16 studies as a primary endpoint, and in 12 studies in RW. QoE for T25FW as OM was graded as high. MDC for T25FW, although rarely formally investigated, is reported at ≥2.7 s or 24%–36%, referring to a baseline measure with a lower cutoff of ≥0.6 s or 12% for pwMS with unrestricted walking ability (EDSS < 4.0) [11, 12]. CRC of T25FW ranges from ≥1.4 s or 10.4%–20%, which is mostly applied as the threshold. Due to the proximity of MDC and CRC thresholds, sensitivity for change is limited and requires confirmation over time. Generalizability applies to all pwMS with walking ability (EDSS = 0–6.5) with good to excellent test–retest reliability and no relevant IRV. T25FW is easily applicable in RW due to the very short administration time of approximately 1 min and because it can be obtained by trained staff as well as the treating neurologist.

Thus, T25FW is clinically useful in quantitatively assessing walking impairment over time and is deemed as a core measure in patients with progressive MS and an aspirational OM for all pwMS with preserved walking ability (EDSS = 0–6.5), especially in those with suspicion of imminent progressive MS (PMS).

#### Symbol Digit Modalities Test

The Symbol Digit Modalities Test (SDMT), a test primarily measuring cognitive processing speed, was employed as OM in 52 studies (eight as a primary endpoint) on 22,307 pwMS, with eight studies undertaken in RW. QoE for SDMT as OM received a grade of high. MDC of SDMT has rarely been formally investigated but is reportedly ≥12% referring to a baseline measure, although this was only studied in pwMS with EDSS between 4.0 and 6.5 [13]. CRC is widely considered at ≥20% or ≥4 points, but a well‐designed study found that CRC may be more appropriate at ≥7–10 points depending on age, intelligence quotient, and educational level [14]. Thus, SDMT provides moderate sensitivity for change but requires confirmation over time. SDMT is generalizable to most pwMS (EDSS = 0–9.0), as there is also an oral version available, it provides good to excellent test–retest reliability, and it has no relevant IRV. However, SDMT shows modest practice effects if testing frequency is <12 months, even when test keys are interchanged, limiting the frequency of application [15]. SDMT is applicable in RW due to the short administration time of approximately 5 min and because it can be obtained by trained staff as well as the treating neurologist. Moreover, a computerized version is available and provides normalized results to healthy controls matched for age, sex, and education level [16]. Hence, SDMT is likely to be clinically useful in quantitatively assessing processing speed over time and is deemed as an aspirational OM for all pwMS without severe cognitive impairment.

#### Nine Hole Peg Test

The Nine Hole Peg Test (9HPT), a measure of manual dexterity, was applied as OM in 28,261 pwMS and 50 studies, of which six took place within RW. QoE rating for 9HPT as OM yielded a grade of moderate to high. MDC and CRC for 9HPT were only rarely formally investigated. MDC is consistently reported at ≥18%–29% from a baseline measure; reported CRC ranges from >1 SD over ≥15%–≥20%, which is mostly applied as the threshold [17]. Due to the proximity of MDC and CRC thresholds, sensitivity for change is limited and requires confirmation over time. Generalizability applies to most pwMS (EDSS = 0–8.5), with excellent test–retest reliability and no relevant IRV. 9HPT is applicable in RW considering the short administration time of approximately 2–5 min and because it can be obtained by trained staff as well as the treating neurologist. However, it shows only a weak correlation with quality of life and identifies only a very low percentage of additional disability worsening not already captured by EDSS and T25FW. Therefore, it likely contributes minimal independent value.

9HPT is likely clinically useful in quantitatively assessing manual dexterity over time and is suggested as an aspirational OM for all pwMS with EDSS < 8.5, especially in those with PMS (or suspicion of imminent PMS) and those without walking ability (EDSS ≥ 7.0).

#### Knowledge and technology gaps for T25FW, SDMT, 9HPT

Open knowledge gaps concern the confirmation and refinement of MDC/CRC in larger cohorts with MS populations comprising a wider scope of age and level of disability, but also gender and ethnicity. As T25FW, SDMT, and 9HPT are very suitable for digitalization and self‐application by the patient, advancement of digital and smart solutions with a focus on standardization and broad applicability represent important technology gaps.

#### Wearables and digital outcome measures

A variety of studies using wearables and digital mobility outcome measures (DMOs) as OM was identified in the search process. The vast majority used various types of accelerometry or sensors, producing a plethora of measures aiming to reflect walking ability, with some also aiming to measure upper extremity function, that is, dexterity. Currently, none of the studied wearables and DMOs is useful as an OM in clinical routine practice, but they can be useful in studies or very specific settings (i.e., clinical rehabilitation or walking interventions). Open knowledge gaps remain regarding investigation of MDC and CRC in larger cohorts with MS populations comprising a wider scope of age and level of disability. Similarly, there is a need for analyses of clinical usefulness and ecological validity.

If clinical usefulness is shown, wearables and DMOs would require advancement of smart solutions with a focus on standardization and broad applicability, including patients' compliance, which would then represent important technology gaps.

### Objectively measurable paraclinical outcomes

We identified 429 studies, of which 410 on 33 different objectively measurable paraclinical outcomes were finally included (see Figure 1). Here, we describe only those most relevant for assessing treatment response in clinical practice. Table S4 provides a detailed summary.

#### New/enlarging T2 lesions

New/enlarging T2 lesions (T2Ls) on brain MRI were used as OM in 249 studies comprising 184,673 pwMS, with the majority (140) conducted within RW and as either primary or secondary endpoint. QoE for new/enlarging T2Ls as OM was rated as high. MDC was relatively broad and rarely investigated, varying from 0.13 to 6.3 lesions, whereas CRC was defined in most studies as ≥1 lesion. However, some studies also defined CRC as either ≥2 or even ≥3 lesions. In that way, sensitivity and specificity for change are likely limited. New T2Ls are generalizable to all relapsing pwMS (pwMS) without contraindications to performing MRI, providing excellent test–retest reliability (intraclass correlation coefficient [ICC] = 0.92–0.95) and good IRV (ICC = 0.82–0.86), although enlarging T2Ls are highly likely to display much lower test–retest and IRV. New T2Ls are applicable in RW and have additional value compared to clinical outcomes by capturing subclinical activity. Enlarging T2Ls are difficult to apply. Although brain MRI needs 30–45 min to be carried out, and an additional 30 min for rating, T2Ls are considered a core measurable among paraclinical measures. Consistent use of CRC definition is definitely one of the knowledge gaps that should be addressed in the near future. As for all MRI‐based measures, standardization of MRI protocols, intervals (including a re‐baseline MRI after DMT initiation or switch), and rating should be bridged, potentially by its digitalization or automatization.

#### Contrast‐enhancing lesions

Gadolinium‐ or contrast‐enhancing lesions (CELs) on brain MRI were used as OM in 235 studies comprising 178,698 pwMS, with the majority (130) conducted within RW and as either primary or secondary endpoint. QoE for CELs as OM was rated as high. MDC was relatively broad and rarely investigated, ranging from 0.5 to 7.8 CEL, whereas CRC was mostly considered ≥1 lesions. By that, its sensitivity and specificity for change are most probably limited. It is generalizable to all RpwMS who do not have contraindications to performing MRI, with excellent test–retest reliability (ICC = 0.92–0.95) and good to excellent IRV (ICC = 0.80–0.96). Like new/enlarging T2Ls, CEL have an additional value to clinical biomarkers by capturing subclinical activity; however, they are limited by the infrequency of events. Although the MRI itself needs 30–45 min to be carried out, and additionally 30 min for rating, it is still applicable in RW. Therefore, CELs are considered a core measurable among paraclinical biomarkers.

#### Whole brain volume

Whole brain volume (WBV), a well‐established imaging marker of neurodegeneration, was used as OM in 138 studies comprising 82,948 pwMS, with 55 conducted within RW. QoE for WBV as OM was rated as moderate to high. MDC was relatively broad and rarely investigated (0.04%–2.14% change per year), and CRC was mostly considered 0.4% change per year but was also rarely formally investigated, varying from 0.20% to 2.18% change per year. WBV has excellent test–retest reliability (ICC = 0.97–0.99) but is susceptible to confounding factors, for example, hydration changes, diurnal fluctuations, lifestyle (smoking, alcohol consumption), medications, menstrual cycle, and comorbidities. Also, IRV considerably depends on the software used, which is one of the main factors prohibiting incorporation into clinical practice to date. WBV measures neurodegenerative processes rather than directly measuring neuroinflammatory disease processes, a target that has not yet been effectively treated in past clinical trials. Besides, WBV requires a relatively long observational time (at least 1 year, if not 2–3 years), and a re‐baseline MRI 3–6 months after DMT initiation to avoid confounding by pseudoatrophy. Although it is time‐consuming and clinically relevant cutoff values including the timeframe for evaluation are unclear in individual patients, it is still applicable in RW and is therefore considered an aspirational measurable.

#### Peripapillary retinal nerve fiber layer thickness

Peripapillary retinal nerve fiber layer (pRNFL) and ganglion cell–inner plexiform layer (GCIPL) thickness, markers of neuroaxonal degeneration measured by optical coherence tomography (OCT), were used as OM in 44/31 studies comprising 6035/5463 pwMS with 27/22 conducted in RW.

QoE for pRNFL/GCIPL thickness as OM was rated as moderate. It is potentially clinically useful, yet with broad MDC (0.48–4.5 μm/0.5–2.7 μm) and rarely studied CRC (1.5–2.0 μm/0.5–1.0 μm). As MDC is probably similar to CRC, both have limited sensitivity and specificity for change, and pRNFL is limited by susceptibility to edema in terms of optic neuritis. However, pRNFL/GCIPL are generalizable to all pwMS who do not have retinal comorbidity or severe myopia (>4–6 diopters), providing excellent test–retest reliability (ICC = 0.99) and IRV (ICC = 0.97–0.99). pRNFL/GCIPL thickness is principally applicable in RW but limited by availability of OCT in clinical settings. OCT requires trained staff to perform but is carried out and rated rather quickly (10–15 min). Therefore, it should be considered an aspirational measurable.

The technology gap that should be addressed is the standardization of OCT protocols, intervals, and rating, which recently have been already partially overcome by quality control criteria, for example, OSCAR‐IB.

#### Neurofilament light chain

Neurofilament light chain (NfL), a major component of the neuronal cytoskeleton that is released into the extracellular space and further into the cerebrospinal fluid (CSF) and the blood after neuroaxonal damage, was used as OM in 68 studies (57 soluble NfL, 19 CSF NfL) comprising 18,131 pwMS, with 34 conducted within RW. QoE for NfL as OM was rated as moderate to high. MDC was broad in both CSF (361.5–3816 pg/mL) and serum (0.53–85.8 pg/mL), whereas CRC was only studied in serum (8–30 pg/mL). Due to the likely close proximity of MDC and CRC thresholds, sensitivity and specificity for change are limited and require confirmation over time. It is generalizable to all pwMS, with moderate to good test–retest reliability (ICC = 0.76–0.81) and probably minimal IRV due to relatively standardized protocols for its detection. However, it is currently not applicable in RW due to low cost–time efficiency, lack of broad and affordable availability, and still‐to‐be‐explored clinical relevance. For now, it seems that a single measurement does not provide enough information but rather it needs to be observed over time in a 3–6‐month interval and be adjusted for age and body mass index (likely by applying percentiles or a *z*‐score). The blood draw can be easily performed in 5 min by any trained staff, with the rating taking an additional 120 min. In that way, it should be considered an aspirational measurable.

Open knowledge gaps regard harmonization and standardization of measurement techniques and reporting of results as well as diagnostic and prognostic cutoff values.

#### Measurable patient‐reported outcomes

The search process identified 280 studies with a total of 119 different patient‐reported outcomes (PROs; see Figure 1). Here, we describe only those most relevant for assessing treatment response in clinical practice. Table S5 provides a detailed summary of all PROs assessed.

More than 90% of the studies investigated did not specifically specify MDC/CRC. Hence, open knowledge gaps concern the development of MDC/CRC in large cohorts with MS populations comprising a wide scope of age and level of disability, but also sex/gender and ethnicity. As PROs are very suitable for digitalization and self‐application, advancement of digital and smart solutions with a focus on standardization and broad applicability represent important technology gaps.

#### Health‐related quality of life (HRQoL): Targeted measures for HRQoL specific to MS

The 29‐item Multiple Sclerosis Impact Scale (MSIS‐29) inquires regarding the impact of MS on a person's everyday life (e.g., difficulties moving around, being dependent of others, limitations to their social life). It generates two subscales that measure the psychological (nine items) and physical (20 items) impact of MS on an individual. Each subscore ranges from 0 (no impairment) to 100 (maximum impairment). A handful of studies have reported the CRC to be determined at eight points. Data on MDC are not reported. Thus, data are insufficient to assess sensitivity and specificity for change. All in all, MSIS‐29 is the most extensively used PRO for HRQoL, and it has been applied in 56 studies on 25,570 pwMS. It has been validated in different populations of pwMS [18]. MSIS‐29 is applicable in RW, as it is easy to use and has potential to be digitalized and an acceptable completion time (5–10 min). Even though the lack of evidence for MDC and CRC currently limits its usefulness, we classify it as an aspirational OM for clinical routine.

#### Depression, anxiety, and mental health

The Hospital Anxiety and Depression Scale (HADS) is a 14‐item questionnaire investigating symptoms of depression and anxiety, generating two separate subscales (HADS‐A and HADS‐D) [19]. It is the most extensively used screening tool for anxiety and depression in our sample (39 studies, 14,391 pwMS), yet it had not been developed specifically for pwMS. Two studies suggest the CRC to be 2 points per subscale. Fourteen years after its initial validation, a literature review reported on its validity in 747 articles and found HADS to perform well in assessing the symptom severity of anxiety disorders and depression in somatic, psychiatric, and primary care patients and in the general population [20]. The correlations between the two subscales varied from 0.40 to 0.74 (mean = 0.56). Cronbach alpha for HADS‐A varied from 0.68 to 0.93 (mean = 0.83) and for HADS‐D from 0.67 to 0.90 (mean = 0.82). In most studies, an optimal balance between sensitivity and specificity was achieved when defined by a score of 8 or above on both HADS‐A and HADS‐D.

HADS is applicable in RW, as it is easy to use and has the potential to be digitalized. Its completion time (5–10 min) is similar to other measures for depression. Even though the lack of evidence for MDC and CRC currently limits its usefulness, we still classify it as an aspirational OM. In comparison to other measures for depression, it investigates symptoms for both depression and anxiety, generating two different subscores. It has the potential to demonstrate possible changes in clinical depression or anxiety disorder for pwMS over time.

#### Fatigue

The Fatigue Scale for Motor and Cognitive Fatigue (FSMC) is a 20‐item questionnaire that assesses fatigue severity in pwMS and generates two subscores (motor and cognitive fatigue). It has been used in 13 studies on 1113 pwMS. FSMC is highly sensitive and specific in detecting fatigue in pwMS. In the validation study, both subscales significantly differentiated between pwMS and controls. Internal consistency (Cronbach alpha > 0.91) as well as test–retest reliability (*r* > 0.80) were high [21]. Values for CRC and MDC are not consistently reported. FSMC is a more elaborate fatigue scale in pwMS that takes slightly longer time to complete (5–10 min). It is applicable in RW, as it is easy to use and has the potential to be digitalized. With its two subscales, it generates a more refined understanding of fatigue. Even though the lack of evidence for MDC and CRC currently limits its usefulness, we still classify it as an aspirational OM, as it has undergone sound validation processes.

#### Specific motor symptoms: Ambulation

The Multiple Sclerosis Walking Scale (MSWS‐12) is a widely used MS‐specific PRO assessing a person's walking capabilities. In our sample, we found 52 studies reporting its use on 12,964 pwMS. Some of these studies report the MDC at 4–6 points and CRC at 5–8 points. In the validation study, test–retest reproducibility was high (≥0.78), scaling assumptions were satisfied, and reliability was high (≥0.94) [22].

MSWS‐12 is the most widely studied PRO for ambulation and easily applicable, as it takes 2–5 min to complete. Therefore, it is applicable in RW and has the potential to be digitalized. Even though the lack of evidence for MDC and CRC currently limits its usefulness, we still classify it as an aspirational OM.

#### Treatment satisfaction

The Treatment Satisfaction Questionnaire for Medication (TSQM) is a 14‐item scale investigating the level of satisfaction with medication, and it is suggested to be a good predictor of patients' medication adherence [23]. In our sample, we found 22 studies using TSQM on 8310 pwMS. Test–retest reliability has not been reported, and we did not find information on MDC and CRC either.

TSQM is the most widely studied PRO for medication treatment satisfaction and easily applicable, as it takes 5–10 min to complete. Therefore, we believe it to be applicable in RW, and it has the potential to be digitalized. Even though the lack of evidence for MDC and CRC currently limits its usefulness, we still classify it as an aspirational OM.

#### Patient‐Reported Outcomes Measurement Information System

The Patient‐Reported Outcomes Measurement Information System (PROMIS) has been developed by the National Institutes of Health with the aim of creating reliable and precise tools to measure PROs across various diseases and conditions. Regarding MS, there are various versions of PROs available, for example, to assess physical function (PROMISnq Short Form v2.0 PF–Multiple Sclerosis 15a) and fatigue (PROMIS–Fatigue 8a), which have been cumulatively used in eight studies on 458 pwMS. Internal consistency reliability estimates and test–retest ICCs are excellent (>0.90), with MDC set at ≥1 point for PROMIS measures and CRC at ≥2–3 points [24, 25]. The completion time varies depending on the versions used, with a range from 5 to 30 min. PROMIS measures are applicable in RW, as they are easy to use and have the potential to be digitalized. Although the low number of studies actually using them as OM currently limits their usefulness, we still classify PROMIS measures as an aspirational OM due to the sound validation processes.

### Combinations of measurable outcomes

The search process identified 135 studies, of which 132 on 32 different combinations of OMs were finally included (see Figure 1). Here, we describe only those OM combinations most relevant for assessing treatment response in clinical practice. Table S6 provides a detailed summary.

#### No evidence of disease activity with two dimensions

No evidence of disease activity with two dimensions (NEDA‐2), a composite measure of relapses and EDSS worsening, was used as OM in 25,676 pwMS and 12 studies as an endpoint, with the majority (10) conducted within RW but none as primary endpoint. QoE for NEDA‐2 as OM was rated as low to moderate, as all studies were observational. Like every composite measure, NEDA‐2 retains all the limitations of its ingredients. NEDA‐2 can be generalized to all patients with RMS (but not PPMS) and is clinically useful, although limited by the rarity of both relapses and EDSS as well as its low sensitivity for disease activity, which is formally represented by a complete congruence of MDC and CRC. NEDA‐2 is well applicable in RW but requires evaluation by a trained neurologist without the possibility for outsourcing to trained staff or digitalization/self‐application by the patient. As both relapses and EDSS worsening are core OMs within the RMS population, NEDA‐2 is also considered as a core measurable.

#### No evidence of disease activity with three dimensions

No evidence of disease activity with three dimensions (NEDA‐3), a composite measure of relapses, EDSS worsening, and MRI activity, was applied as an explicit OM in 41,829 pwMS and 76 studies (exclusively as a retrospectively defined post hoc endpoint), of which 40 took place within RW. QoE for NEDA‐3 as OM yielded grades of low to moderate. NEDA‐3 retains all the limitations of NEDA‐2 and its composites. “MRI activity” in NEDA‐3 is based on measuring T2Ls and/or CELs, which display only weak correlation with treatment response [26, 27]. There is no consensus on the definition of MRI activity in NEDA‐3, which ranges from only new T2Ls to new/enlarging T2Ls and consideration to no consideration of CELs.

NEDA‐3 can be generalized to all patients with relapsing MS (but not PPMS) and access to regular MRI follow‐up investigations. External validity in terms of test–retest reliability has not been formally studied but is likely limited by the IRV of EDSS and more importantly, of MRI activity, where especially new and enlarging T2Ls display poor IRV. The clinical usefulness of NEDA‐3 is often per se assumed, but NEDA‐3 is not validated as a surrogate marker of treatment response. Although MDC and CRC have not been formally studied, there is a complete congruence of MDC and CRC due to the dichotomous definition of NEDA‐3 and thus, very low sensitivity and specificity for change over time. Loss of NEDA‐3 is mostly driven by MRI activity, which is the parameter with the weakest association with treatment outcome, introducing a relevant imbalance to the composite measure.

Applicability in RW is dependent on access to regular MRI follow‐up but requires evaluation of relapses and EDSS by a trained neurologist as well as standardized MRI acquisition rated by trained neuroradiologists using a consensus definition of MRI activity.

Currently, NEDA‐3 can only be considered as an aspirational OM in MS, applicable at centers that can provide these prerequisites.

#### Knowledge gaps for NEDA‐2/3

To establish NEDA‐2/3 as the universally applied OM, multiple steps are required. The first is to compare definitions of relapse/EDSS worsening/MRI activity and confirmation intervals with thorough and sound methodology to establish consensus definitions and confirmation intervals. The second is consistent and rigorous training of raters. This step also requires standardization of MRI protocols, which could be aided by digitalization and automatization of MRI analyses, which therefore represents an important technology gap. Finally, the clinical value of targeting NEDA‐2/3 would have to be confirmed, again by thorough and sound methodology in larger cohorts with MS populations comprising a wider scope of age and level of disability.

#### Cognitive composite outcomes

The Brief International Cognitive Assessment for MS (BiCAMS), a composite of SDMT, California Verbal Learning Test‐II (CVLT‐II), and Brief Visuospatial Memory Test Revised (BVMT‐R), was applied as OM in nine studies (in one as a primary endpoint) on 731 pwMS, with one study conducted in RW.

The Brief Repeatable Battery (BRB), a composite of Selective Reminding Test (SRT), 10/36 Spatial Recall Test (SPART), SDMT, Paced Auditory Serial Addition Test (PAST), and Word List Generation (WLG), was used as OM in 565 pwMS and seven studies (in one as a primary endpoint), with one study conducted in RW. QoE rating for BiCAMS and BRB as OM yielded grades of low to moderate. MDC for the composites BiCAMS and BRB has not been formally studied and is known only for SDMT. CRC was only rarely investigated and is estimated at ≥10% and as worsening in ≥2 of the five domains, respectively. Consequently, data are insufficient to assess sensitivity and specificity for change.

Despite the small available sample size, BiCAMS and BRB can likely be generalized to most pwMS without severe visual or cognitive impairment, with good test–retest validity and no relevant IRV. However, both BiCAMS and BRB require considerable time resources (administration time = 20–25 min and 30–40 min, respectively), and independent contribution to information on health utility has not been shown. Therefore, neither BiCAMS nor BRB is currently useful or applicable as an OM in routine clinical practice, although they could be useful in studies or very specific settings (i.e., clinical rehabilitation or cognition interventions). Open knowledge gaps pertain to data on MDC/CRC and testing intervals (including assessment of practice effects depending on intervals) as well as analyses of clinical usefulness and ecological validity. If clinical usefulness is shown, BiCAMS and/or BRB would be suitable for digitalization and self‐application by the patient, in which case advancement of digital and smart solutions with a focus on standardization and broad applicability would represent technology gaps.

#### Virtual composite measures

The current state of evidence and knowledge/technology gaps regarding virtual composite measures such as the Floodlight or the DREAMS applications can be summarized, as there are no studies available on MDC and CRC, which is why sensitivity and specificity for change cannot be assessed currently. Virtual composite measures would be generalizable to most pwMS with walking ability (EDSS = 0–6.5) and without severe cognitive or visual impairment as well as access/willingness/ability to perform these tests on an electronic device. External validity regarding test–retest reliability is currently not sufficiently studied; no relevant IRV is expected. Applicability in RW is strongly dependent on availability of electronic devices as well as data collection and software solutions enabling storage, visualization, and interpretation of results. Currently, virtual composite measures are neither useful nor applicable as OMs in routine clinical practice but could be useful in studies or very specific settings. Open knowledge gaps remain regarding investigation of MDC and CRC in larger cohorts with MS populations comprising a wider scope of age and level of disability. Similarly, there is a need for analyses of clinical usefulness and ecological validity. Technology gaps comprise development of data collection and software solutions enabling storage, visualization, and interpretation of results as discussed.

## SUMMARY AND CONCLUSIONS

This SLR on four groups of MS outcomes has yielded a plethora of different OMs. The QoE and the state of knowledge as a basis for establishing a treat‐to‐target approach beyond the traditional mainstays of OMs, relapses and EDSS, are not overly strong.

**FIGURE 2:**
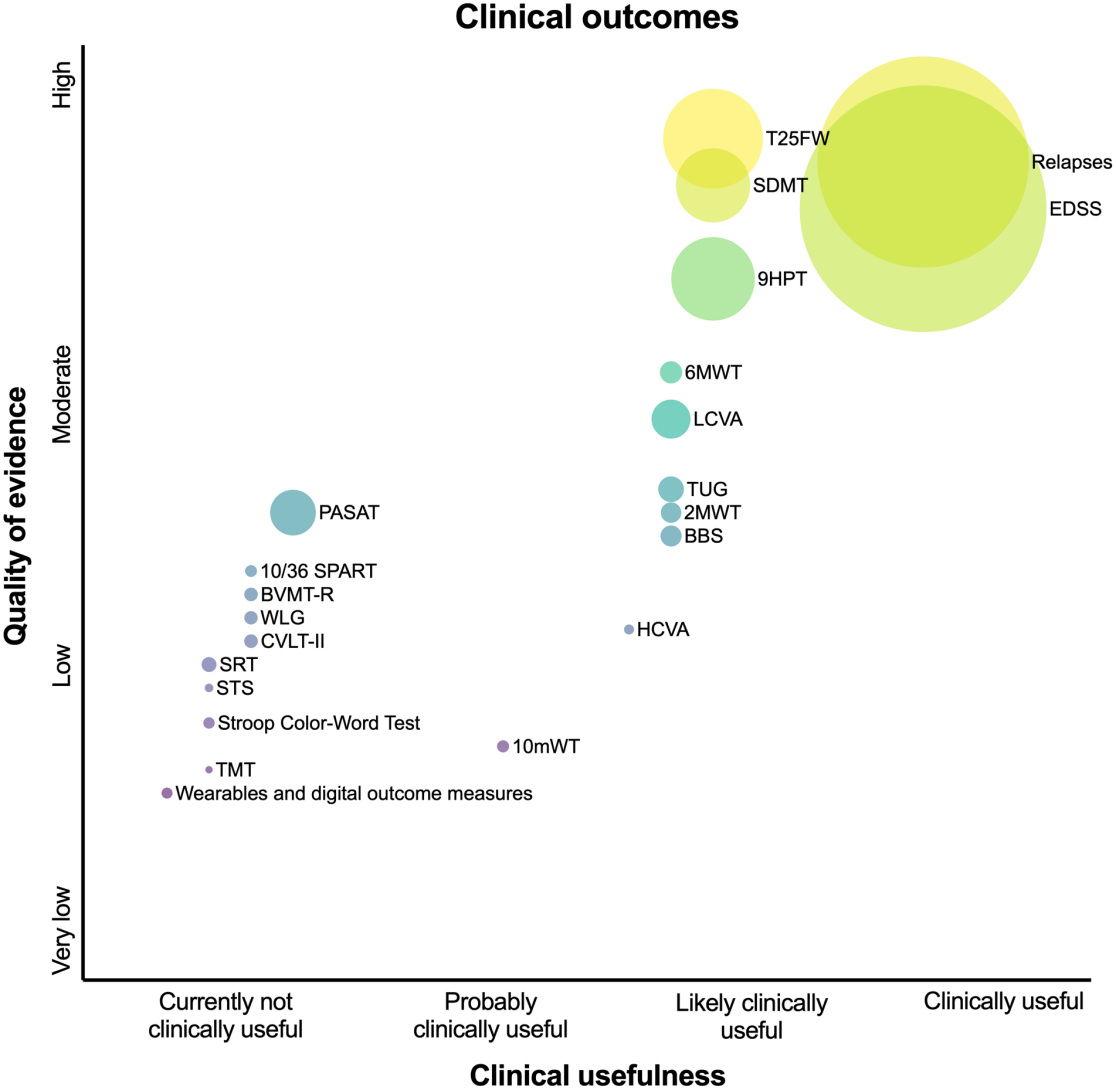
Clinical usefulness and quality of evidence for clinical outcomes in multiple sclerosis. Size of circles indicates available sample size of underlying evidence. 2MWT, 2 minute walking test; 6MWT, 6 minute walking test; 9HPT, 9‐Hole Peg Test; 10mWT, 10 minute walking test; BBS, Berg Balance SCale; BVMT‐R, Brief Visuospatial Memory Test Revised; CVLT‐II: California Verbal Learning Test II; EDSS, Expanded Disability Status Scale; HCVA, High Contrast Visual Acuity; LCVA, Low Contrast Visual Acuity; PASAT, Paced Auditory Serial Addition Test; SDMT, Symbol Digit Modalities Test; SPART, Spatial Recall Test; SRT, Selective Reminding Test; TMT, trail making test; TUG, Timed Up and Go Test; T25FW, Timed 25 Foot Walk Test; WLG, Word List Generation.

**FIGURE 3:**
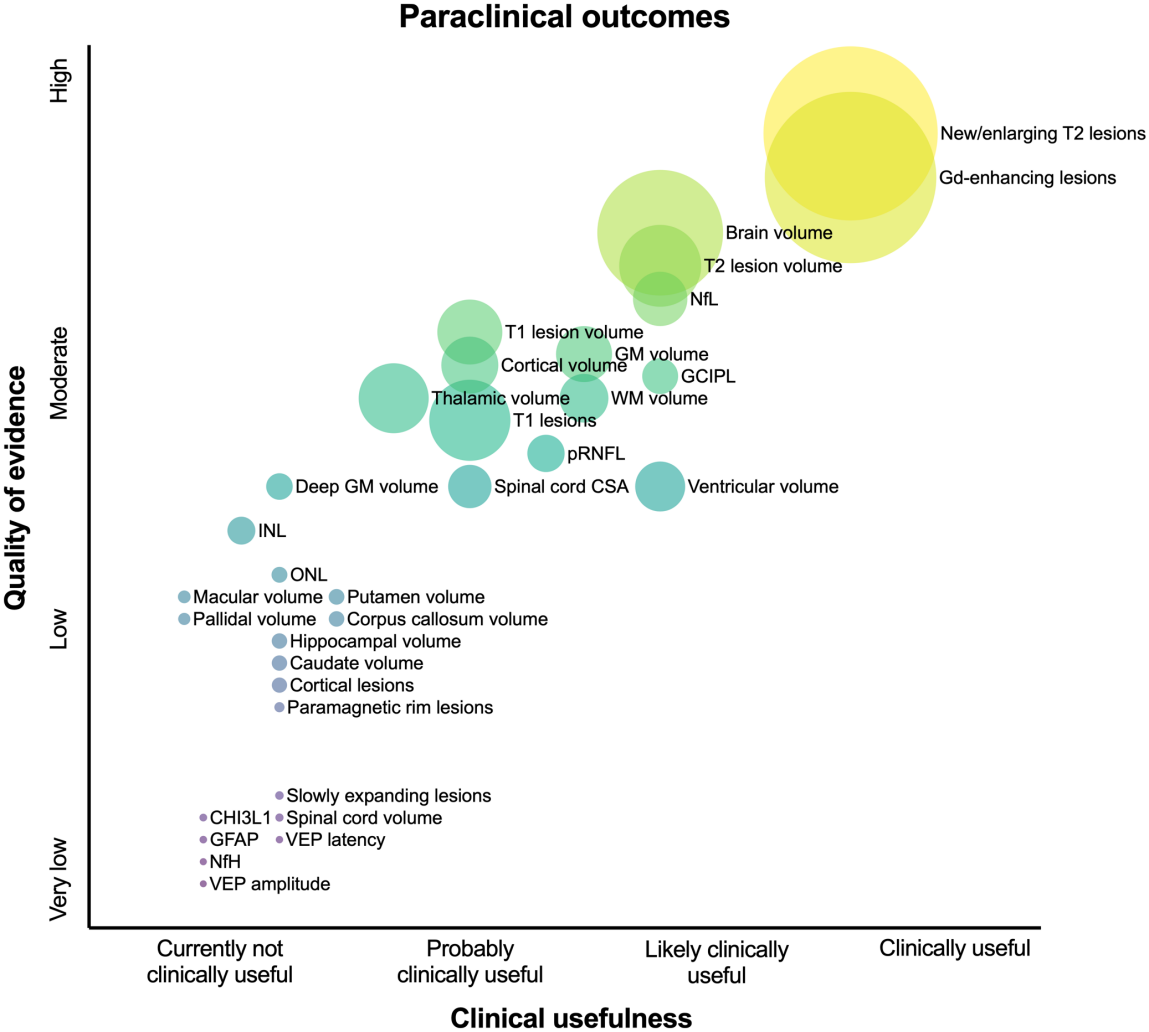
Clinical usefulness and quality of evidence for paraclinical outcomes in multiple sclerosis. Size of circles indicates available sample size of underlying evidence. CHI3L1, Chitinase 3 like 1; CSA, Cross Sectional Area; GCIPL, Ganglion Cell and Inner Plexiform Layer; GFAP, Glial Fibrillaric Astrocytic Protein; GM, Grey Matter; INL, Inner Nuclear Layer; NfH, Neurofilament Heavy chain; NfL, Neurofilament Light chain; ONL, Outer Nuclear Layer; pRNFL, peripapillary retinal nerve fiber layer; WM, White Matter; VEP, Visual Evoked Potentials.

**FIGURE 4:**
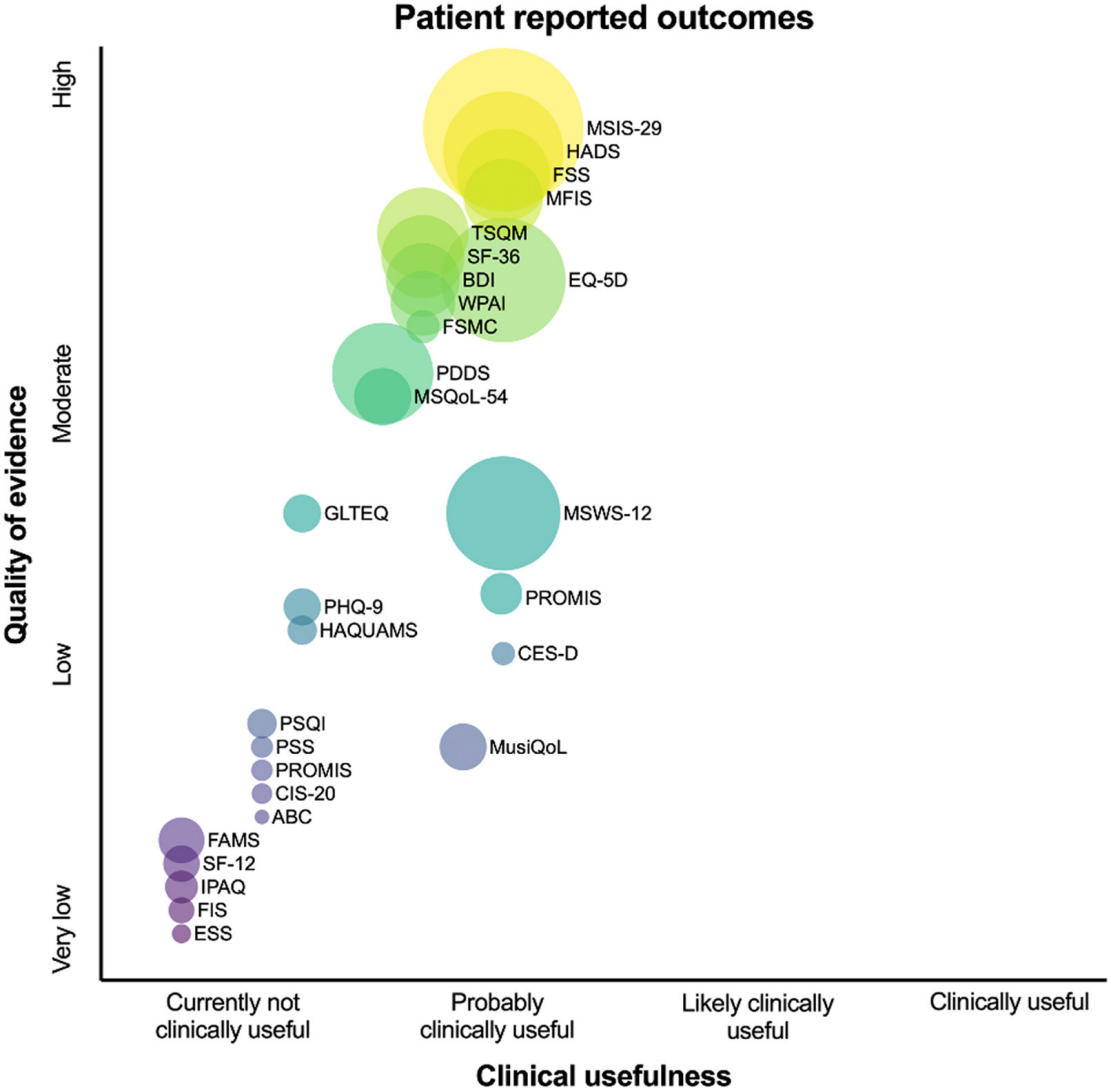
Clinical usefulness and quality of evidence for patient‐reported outcomes in multiple sclerosis. Size of circles indicates available sample size of underlying evidence. ABC, Activities‐Specific Balance Confidence Scale; BDI, Beck Depression Inventory; CES‐D, Center for Epidemiological Studies Depression Scale; CIS‐20, Checklist Individual Strength; EQ‐5D, The EuroQol 5 Dimensions questionnaire; ESS, Epworth Sleepiness Scale; FAMS, Functional Assessment of Multiple Sclerosis; FIS, Fatigue Impact Scale; FSS, Fatigue Severity Scale; FSMC, Fatigue Scale of Motor and Cognitive Fatigue; GLTEQ, Godin Leisure‐Time Questionnaire; HADS, Hospital Anxiety and Depression Scale; HAQUAMS, Hamburg Quality of Life Questionnaire in Multiple Sclerosis; IPAQ, International Physical Activity Questionnaire; MFIS, Modified Fatigue Impact Scale; MSIS‐29, Multiple Sclerosis Impact Scale; MSQoL‐54, Multiple Sclerosis Quality of Life‐54; MSWS‐12, Multiple Sclerosis Walking Scale; MusiQoL, Multiple Sclerosis International Quality of Life; PDDS, Patient Determined Disease Steps; PHQ‐9, Patient Health Questionnaire; PSS, Perceived Stress Scale; PROMIS, Patient‐Reported Outcomes Measurement Information System; PSQI, Pittsburgh Sleep Quality Index; SF‐12, 12‐item Short Form Survey; SF‐36, 36‐item Short Form Survey; TSQM, Treatment Satisfaction Questionnaire for Medication; WPAI, Work Productivity and Activity Impairment.

**FIGURE 5:**
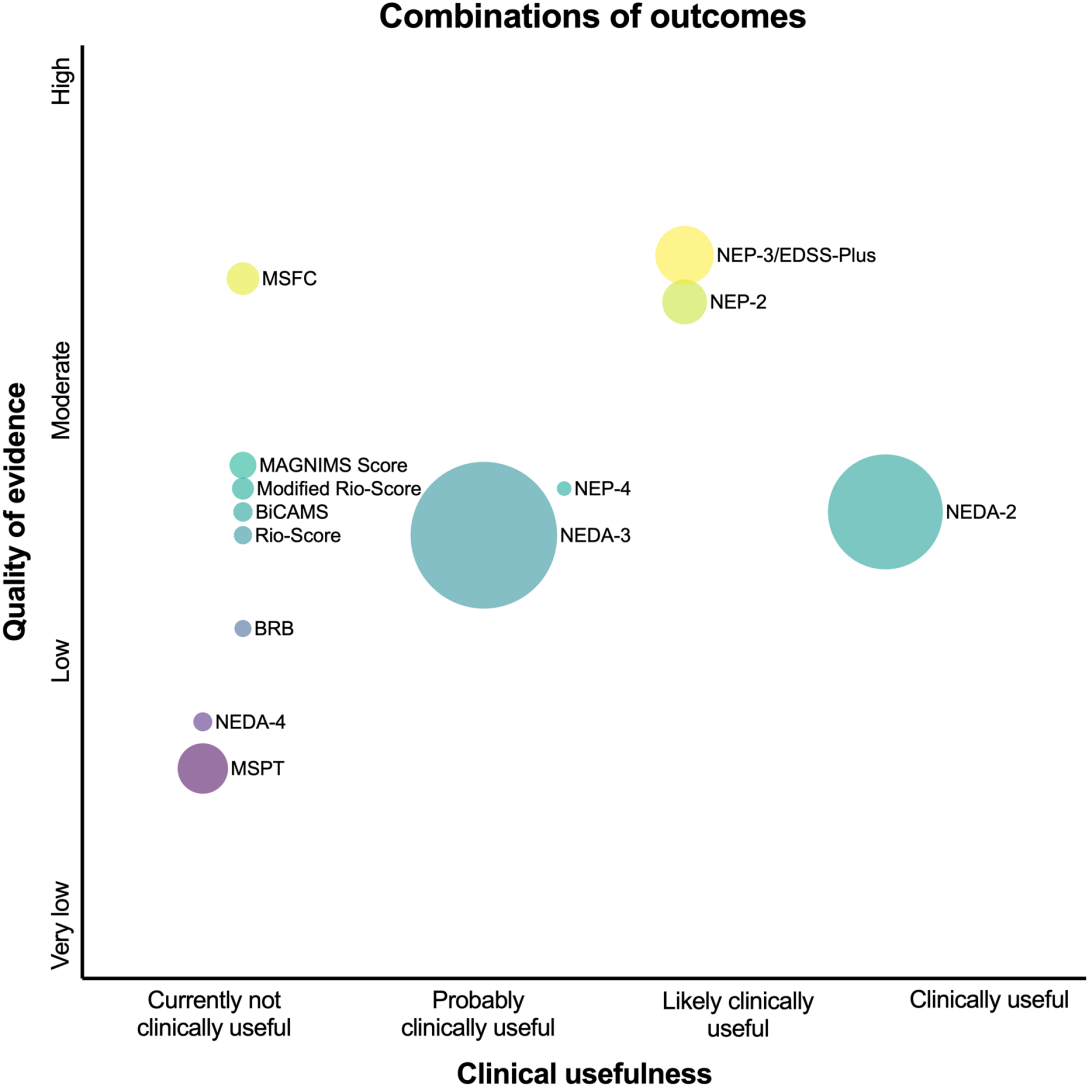
Clinical usefulness and quality of evidence for combinations of outcomes in multiple sclerosis. Size of circles indicates available sample size of underlying evidence. BiCAMS, Brief International Cognitive Assessment for MS; BRB, Brief Repeatable Battery; MSFC, Multiple Sclerosis Functional Composite; MSPT, Multiple Sclerosis Performance Test; MRS, Modified Rio‐Score; NEDA‐2, No Evidence of Disease Activity 2; NEDA‐3, No Evidence of Disease Activity 3; NEDA‐4, No Evidence of Disease Activity 4; NEP‐2, No Evidence of Progression 2; NEP‐3, No Evidence of Progression 3; NEP‐4, No Evidence of Progression 4.

Although there are numerous promising candidates, there is a striking lack of indispensable evidence for implementation in clinical routine (see Figures 2, 3, 4, 5). This is especially relevant when defining “clinical routine” beyond the realities of specialized academic centers, where availability of resources in personnel, time, and costs may be much more restrictive. Efforts to improve this issue should start by investigating these candidate measures in large cohorts with different MS populations reflecting the RW variability in age, gender, ethnicity, disability level, and relevant comorbidities, establishing reliable thresholds of MDC and CRC, as well as describing external and ecological validity under these circumstances. From there, a broad consensus on a set of OMs including a standardized testing protocol needs to be established based on this evidence. For these measures (and also for others of interest in special settings), practicable means for data collection and software solutions enabling storage, visualization, and interpretation of results have to be developed. An emphasis should be placed on developing OMs by digitalized self‐applications and self‐testing by pwMS to widen the spectrum of outcomes but also to save precious resources of personnel and time in clinical practice.

A proposal of a set of measurable MS outcomes was created for two different scenarios (see Table 1):

1. Core measurables required for every pwMS in every routine clinical setting
2. Advanced/aspirational measurables requiring additional resources.

**TABLE 1:** Core and aspirational measurables for people with MS in the routine clinical setting.

Measure	Clinical outcomes	Paraclinical outcomes	Patient‐reported outcomes	Combination of outcomes
Core measures				
RMS	Relapses, EDSS	New/enlarging T2 lesions, Gd‐enhancing lesions	None	NEDA‐2
PMS	EDSS	New/enlarging T2 lesions, Gd‐enhancing lesions	None	
If EDSS ≤ 6.5	T25FW	New/enlarging T2 lesions, Gd‐enhancing lesions	None	NEP‐2
Aspirational measures				
RMS	T25FW (+9HPT), SDMT	Brain volume, T2 lesion volume, sNfL, pRNFL and GCIPL thicknesses	BDI/HADS MSIS‐29/MSQoL‐54 MSWS‐12 FSS/MFIS/FSMC TSQM	NEDA‐3 and NEP‐3
PMS and EDSS ≤ 6.5	T25FW, 9HPT, SDMT	Brain volume (sNfL)		NEP‐3/EDSS plus
PMS and EDSS ≥ 7	9HPT, SDMT	Brain volume (sNfL)	BDI/HADS MSIS‐29/MSQoL‐54 FSS/MFIS/FSMC TSQM	NEP‐3/EDSS plus

Abbreviations: 9HPT, Nine Hole Peg Test; BDI, Beck Depression Inventory; EDSS, Expanded Disability Status Scale; FSMC, Fatigue Scale for Motor and Cognitive Fatigue; FSS, Fatigue Severity Scale; GCIPL, ganglion cell and inner plexiform layer; Gd, gadolinium; HADS, Hospital Anxiety and Depression Scale; MFIS, Modified Fatigue Impact Scale; MS, multiple sclerosis; MSIS‐29, 29‐item Multiple Sclerosis Impact Scale; MSQoL‐54, Multiple Sclerosis Quality of Life‐54; MSWS‐12, Multiple Sclerosis Walking Scale; NEDA, no evidence of disease activity; NEP, no evidence of progression; PMS, progressive MS; pRNFL, peripapillary retinal nerve fiber layer; RMS, relapsing MS; SDMT, Symbol Digit Modalities Test; sNfL, soluble neurofilament light chain; T25FW, Timed 25 Foot Walk Test; TSQM, Treatment Satisfaction Questionnaire for Medication.

## AUTHOR CONTRIBUTIONS

**Gabriel Bsteh:** Conceptualization; data curation; writing – original draft; methodology. **Nik Krajnc:** Data curation; writing – original draft; methodology. **Patrick Altmann:** Data curation; writing – original draft; methodology. **Barry Hendin:** Writing – review and editing. **Trishna Bharadia:** Writing – review and editing. **Sonja Jaruszowic:** Writing – review and editing. **Fred Lublin:** Writing – review and editing. **Jiwon Oh:** Writing – review and editing. **Detlev Parow:** Writing – review and editing. **Annemie Ribbens:** Writing – review and editing. **Aoife Shields:** Writing – review and editing. **Dirk Smeets:** Writing – review and editing. **Eric Thouvenot:** Writing – review and editing. **Andrew Chan:** Writing – review and editing. **Thomas Berger:** Writing – review and editing; conceptualization; supervision.

## FUNDING INFORMATION

This review was conducted as an academic partner for the Multiple Sclerosis Quality of Care Alliance funded by Novartis.

## CONFLICT OF INTEREST STATEMENT

G.B. has participated in meetings sponsored by or received speaker honoraria or travel funding from Biogen, Celgene/BMS, Janssen, Lilly, Merck, Novartis, Roche, Sanofi‐Genzyme, and Teva and has received honoraria for consulting from Biogen, Celgene/BMS, Janssen, Merck, Novartis, Roche, Sanofi‐Genzyme, and Teva. He has received unrestricted research grants from Celgene/BMS and Novartis. N.K. has participated in meetings sponsored by or received speaker honoraria or travel funding from Alexion, BMS/Celgene, Janssen‐Cilag, Merck, Novartis, Roche, and Sanofi‐Genzyme and has held a grant for a Multiple Sclerosis Clinical Training Fellowship Program from the European Committee for Treatment and Research in Multiple Sclerosis. P.A. has participated in meetings sponsored by or received speaker honoraria or travel funding from Biogen, Merck, Roche, Sanofi‐Genzyme, and Teva and has received honoraria for consulting from Biogen. He received a research grant from Quanterix International and was awarded a combined sponsorship from Biogen, Merck, Sanofi‐Genzyme, Roche, and Teva for a clinical study. B.H. has participated in meetings sponsored by and received honoraria (lectures, advisory boards, consultations) from Novartis, Genentech, Biogen, Sanofi, EMD Serono, Amgen, Bristol, Myers, Squibb, and Alexion. T.Bh. has received honoraria and consulting fees from AbbVie, Boehringer Ingelheim, Bristol Myers Squibb, Envision Pharma, Flatiron UK, Heel Pharma, Hollister, Insmed, King's College London, Medable, Medidata, Merck, MSD, Novartis, Oxford Health, Parexel, Pfizer, Queen Mary University London, Roche, Sandoz, Servier, Talking Medicines, Teva, UCB, University College London, and University of Nottingham. S.J. has participated in meetings sponsored by Merck, Roche, Sanofi‐Genzyme, and Teva and has received honoraria for consulting from Novartis. F.L. has participated in meetings sponsored by and received honoraria (lectures, advisory boards, consultations) from Biogen, EMD Serono, Novartis, Actelion/Janssen, Sanofi‐Genzyme, Roche/Genentech, Horizon Therapeutics/Amgen, Celgene/BMS, Mapi Pharma, Brainstorm Cell Therapeutics, Mylan/Viatris, Immunic, Avotres, Neurogene, LabCorp, Entelexo Biotherapeutics, Neuralight, SetPoint Medical, Hexal/Sandoz, Baim Institute, Sudo Biosciences, Lapix Therapeutics, Biohaven Pharmaceuticals, Abata Therapeutics, Cognito Therapeutics, and ImmPACT Bio. His institution has received financial support for research from Biogen, Novartis, Sanofi, NMSS, NIH, and Brainstorm Cell Therapeutics. J.O. has received grant funding from Biogen‐Idec, Roche, and EMD‐Serono and has received personal compensation for consulting or speaking from Biogen‐Idec, BMS, EMD‐Serono, Eli Lilly, Horizon Therapeutics, Novartis, Roche, and Sanofi‐Genzyme. D.P. has received honoraria for consulting and advising from Celgene/BMS, Janssen, and Novartis. A.R. has been an employee of icometrix (a company aiming to bring relevant outcome measures to clinical practice). A.S. has participated in meetings sponsored by and received honoraria (lectures, advisory boards, consultations) from pharmaceutical companies marketing treatments for MS including Biogen, Merck, Mylan, Novartis, Roche, Sanofi‐Genzyme, and Viatris. D.S. has been an employee of icometrix (a company aiming to bring relevant outcome measures to clinical practice). E.T. has received consulting and lecturing fees, travel grants, or unconditional research support from the following pharmaceutical companies: Actelion, Biogen, BMS, Merck, Novartis, Roche, and Teva Pharma. A.C. has received speaker/board honoraria from Actelion (Janssen/J&J), Alexion, Almirall, Bayer, Biogen, Celgene (BMS), Genzyme, Merck, Novartis, Roche, and Teva, all for hospital research funds. He has received research support from Biogen, CSL Behring, Genzyme, UCB, the European Union, and the Swiss National Foundation. T.Be. has participated in meetings sponsored by and received honoraria (lectures, advisory boards, consultations) from pharmaceutical companies marketing treatments for MS including Allergan, Bayer, Biogen, Bionorica, BMS/Celgene, Genesis, GSK, GW/Jazz Pharma, Horizon, Janssen‐Cilag, MedDay, Merck, Novartis, Octapharma, Roche, Sandoz, Sanofi‐Genzyme, Teva, and UCB. His institution has received financial support in the past 12 months by unrestricted research grants (Biogen, Bayer, BMS/Celgene, Merck, Novartis, Roche, Sanofi‐Genzyme, Teva) and for participation in clinical trials in multiple sclerosis sponsored by Alexion, Bayer, Biogen, Merck, Novartis, Octapharma, Roche, Sanofi‐Genzyme, and Teva.

## Supporting information


Data S1.

Data S2.

Data S3.

**Table S1.** Screening list for patient‐reported outcome.

**Table S2.** Full study list.

**Table S3.** Overview of clinical outcomes in multiple sclerosis.

**Table S4.** Overview of paraclinical outcomes in multiple sclerosis.

**Table S5.** Overview of patient reported outcomes in multiple sclerosis.

**Table S6.** Overview of composite outcomes in multiple sclerosis.

## Data Availability

All data used in the manuscript are provided in the Supplements.
