# Supplementary material for: Treating to target in multiple sclerosis: Do we know how to measure whether we hit it?
Source: Eur J Neurol. 2024 Oct 24;31(12):e16526. doi: 10.1111/ene.16526 (PMC11554867; doi:10.1111/ene.16526)
Supplement: Supplementary file 1 — Data S1. [file ENE-31-e16526-s006.docx]

# Supplemental Results

Here, the results of the conducted SLR not decribed in the main manuscript are narratively summarized within the framework of the four groups of MS outcomes (objectively measurable clinical outcomes, objectively measurable paraclinical outcomes, measurable patient-reported outcomes and any combination of those).

Supplementary Table 2 provides a full list of all included studies with extracted data comprising study population, outcome measures analyzed, mode of implementation, MDC, CRC and assessment of bias.

## *Objectively measurable clinical outcomes*

Supplementary Table 3 provides a detailed summary of all objectively measurable clinical outcomes assessed. Here, the clinical outcomes not described in the main manuscript are discussed.

### *Low Contrast Visual Acuity*

Low contrast visual acuity (LCVA) was used as an outcome measure in 6 studies on 6,171 pwMS, in one study as a primary endpoint and in one study within a real-world setting.

Quality of evidence for LCVA as an outcome measure was graded as moderate. MDC for LCVA is reported at ≥0.1 logMAR or ≥7 letters or ≥7% from baseline, while CRC is usually considered at ≥6-7 letters and/or ≥20%. Due to the likely close proximity of MDC and CRC thresholds, sensitivity for change is limited and requires confirmation over time. LCVA can be generalized to all pwMS without severe cognitive impairment, providing moderate to good test-retest reliability, no relevant inter-rater variability and displays no practice effect.

LCVA is quite easily applicable in real-world setting due to the short administration time of about 1-3 minutes and as it can be obtained by trained staff as well as the treating neurologist. LCVA displays significantly stronger correlation with subjective visual impairment as perceived by pwMS than high contrast visual acuity (HCVA), although it does not seem to correlate with QoL questioning its value in clinical practice [1]. Thus, evidence is currently insufficient to consider LCVA an aspirational outcome measure in routine practice, although potentially clinically useful in quantitatively assessing visual impairment over time.

### *Knowledge and technology gaps for LCVA*

Open knowledge gaps concern the confirmation and refinement of MDC/CRC in larger cohorts with MS populations comprising a wider scope of age and level of disability, but also gender and ethnicity. As LCVA is very suitable for digitalization and self-application by the patient, advancement of digital and smart solutions with a focus on standardization and broad applicability represent important technology gaps.

### *Wearables and digital outcome measures*

A variety of studies using wearables and digital mobility outcome measures (DMO) as outcome measures was identified in the search process. The vast majority used various types of accelerometry or sensors producing a plethora of measures aiming to reflect walking ability, with some also aiming to measure upper extremity function, i.e. dexterity. These outcome measures are subsumed here as the state of available evidence is very similar.

Overall, wearables and DMOs were used as an outcome measure in 475 pwMS and 7 studies (in 2 as a primary endpoint), of which none took place within a real-world setting.

Quality of evidence rating for all wearables and DMOs as an outcome measure yielded grades of very low to low. With the exception of one study measuring U turn speed via a smart-phone, there are no studies available on MDC. CRC of wearables and DMOs has not been studied in MS. Thus, sensitivity and specificity for change cannot be assessed currently.

Theoretically, wearables and DMOs would be generalizable to most pwMS, although limitations apply based on walking ability, cognitive impairment (if active measurements are required) or if pwMS were unwilling/unable to carry/use wearables. External validity regarding test-retest reliability is currently not sufficiently clear for any of the used wearables and DMOs, while no relevant inter-rater variability is expected. Applicability in real world is strongly dependent on the mode of testing (with passive monitoring having obvious advantages compared to active monitoring), patient compliance, availability of devices as well as data collection and software solutions enabling storage, visualization and interpretation of results.

Currently, none of the studied wearables and DMOs are useful as an outcome measure in clinical routine practice, but can be useful in studies or very specific settings (i.e. clinical rehabilitation or walking interventions).

Open knowledge gaps remain regarding investigation of MDC and CRC in larger cohorts with MS populations comprising a wider scope of age and level of disability. Similarly, there is a need for analyses of clinical usefulness and ecological validity.

If clinical usefulness was shown, wearables and DMOs would require advancement of smart solutions with a focus on standardization and broad applicability including patients compliance, which would then represent important technology gaps.

## *Objectively measurable paraclinical outcomes*

Supplementary Table 4 provides a detailed summary of all objectively measurable paraclinical outcomes assessed. Here, the paraclinical outcomes not described in the main manuscript are discussed.

### T2 lesion volume

T2 lesion volume on brain MRI, a measurement that has been commonly used to evaluate disease activity but has been lately proven to correlate with disability progression as well (atrophied T2 lesion volume), was used as an outcome measure in 91 studies comprising 41,366 pwMS with 38 conducted within a real-world setting.

Quality of evidence for T2 lesion volume as an outcome measure was rated as moderate to high. MDC was relatively broad and rarely investigated (0.8–39.2 ml), with CRC being only studied once (0.4 ml). Hence, there is insufficient data to assess sensitivity and specificity for change. It is generalizable to all (R)PwMS without contraindications to performing MRI providing excellent test-rest reliability (ICC 0.92–0.95), and probably good to excellent inter-rater reliability but depending on the software used. It is applicable in real world but time consuming, requiring a volumetric software and trained staff to perform it, and clinically-relevant cut-off values including the time-frame for evaluation are unclear. In that way, it is considered an aspirational measurable.

### Thalamic volume

Thalamic volume, another MRI volumetric measure of neuroaxonal damage in MS, was used as an outcome measure in 41 studies comprising 25,249 pwMS with 17 conducted in a real-world setting.

Quality of evidence for thalamic volume as an outcome measure was rated as moderate. MDC was broad and rarely investigated (0.1–4.5 ml and 0.06–5.1%), with CRC studied only once (–0.56%). Therefore, there is insufficient data to assess sensitivity and specificity for change. It is generalizable to all pwMS who do not have contraindications to performing MRI, and has excellent test-retest reliability (ICC 0.92–0.95) and probably good to excellent inter-rater reliability, which depends on the software used. It is currently not applicable in real world as it is time consuming and has no additional independent value. In that way, it is only reserved for studies. Knowledge gaps concern protocol standardization and confirmation of clinical value in larger cohorts with different MS populations.

### Gray matter volume

Gray matter (GM) volume, an outcome reflecting neuronal loss, was used as an outcome measure in 47 studies comprising 18,747 pwMS with 27 conducted in a real-world setting.

Quality of evidence for GM volume as an outcome measure was rated as moderate. MDC was broad and rarely investigated (53.2–131.3 ml and 0.02–10.6%), and CRC was not studied. Hence, there is insufficient data to assess sensitivity and specificity for change. It is generalizable to all pwMS without contraindications to performing MRI providing excellent test-retest reliability (ICC 0.91–0.95) and probably good to excellent inter-rater reliability, depending on the software used. It is, however, currently not applicable in real world and is only reserved for studies.

Open knowledge gaps regard evaluation of clinical value in larger cohorts with different MS populations, with technology gaps concerning standardization of MRI protocols and rating, potentially by digitalization/automatization.

### White matter volume

White matter (WM) volume, another outcome that measures tissue loss but is more susceptible to errors and artefacts due to WM lesions for both technical and biological reasons, was used as an outcome measure in 40 studies comprising 13,992 PwMS with 22 conducted in a real-world setting.

Quality of evidence for WM volume as an outcome measure was rated as moderate. MDC was broad and rarely investigated (81.1–158.5 ml and 0.03–5.88%), and CRC was not studied. Therefore, there is no sufficient data to assess sensitivity and specificity for change. However, it has an advantage of being generalizable to all PwMS without contraindications to performing MRI providing excellent reproducibility (ICC 0.95–0.99) and probably good to excellent inter-rater reliability, depending on the software used. It is, however, currently not applicable in real world, and is still reserved for studies, with a need for standardization of MRI protocols and automatization.

### Paramagnetic rim lesions

Paramagnetic rim lesions (PRL), a biomarker of persistent chronic inflammation, axonal transection and therefore disease progression, were used as an outcome measure in 8 studies comprising 639 PwMS with 2 conducted in a real-world setting.

Quality of evidence for PRL as an outcome measure was rated as very low to low. PRL are currently not clinically useful. MDC was rarely studied (2–5.9), and CRC was studied only once (≥4). Hence, they have a limited sensitivity for change due to proximity/congruence of MDC and CRC. PRL are probably generalizable to all PwMS without contraindications to performing MRI, providing good to excellent inter-rater agreement (0.86–0.99), whereas test-retest reliability was, to our knowledge, never studied. PRL are currently not applicable in real world due to limited availability of the required MRI sequences, and therefore reserved for studies.

### Slowly expanding lesions

Slowly expanding lesions (SEL), also known as smoldering plaques, which are associated with incomplete remyelination, resulting in irreparable myelin loss and axonal degeneration, were used as an outcome measure in 3 studies comprising 316 PwMS with 2 conducted in a real-world setting.

Quality of evidence for SELs as an outcome measure was rated as very low. They are currently not clinically useful, with MDC being measured in different units (number of lesions, ml, mm^3^), and CRC only studied once (≥1 lesion). Hence, there is insufficient data to assess sensitivity and specificity for change. SELs are likely generalizable to all PwMS without contraindications to performing MRI, although with unknown but probably good to excellent test-retest reliability and probably good to excellent inter-rater reliability, too, depending on the software used. They are applicable in real world, but are for now reserved for studies only.

### Spinal cord cross-sectional area

Spinal cord cross-sectional area (CSA) on spinal MRI, another promising biomarker of MS-associated neurodegeneration, especially in (primary) progressive MS, was used as an outcome measure in 13 studies comprising 11,003 PwMS with 8 conducted in a real-world setting.

Quality of evidence for spinal cord CSA as an outcome measure was rated as low to moderate. MDC was broad and rarely investigated (1.55–28.2 mm^2^), with CRC not studied. Thus, there is insufficient data to assess sensitivity and specificity for change. However, it is generalizable to all PwMS without contraindications to performing MRI, providing good to excellent test-retest reliability (ICC 0.84–0.99) and probably good to excellent inter-rater reliability, too, depending on the software used. CSA is currently not applicable in real world and should be reserved for studies only.

### Cortical lesions

Cortical lesions (CL) differ substantially from lesions located in the white matter as far as the histopathological characteristics are concerned, suggesting location-dependent expression of the MS-related immunopathological processes. CL were used as an outcome measure in 15 studies comprising 1,011 PwMS with 5 conducted in a real-world setting. Quality of evidence for CL as an outcome measure was rated as low. It is currently not clinically useful. MDC was rarely investigated (0.2–0.8 mm), and CRC studied only once (≥1 CL). Hence, there is insufficient data to assess sensitivity and specificity for change. CL are generalizable to all PwMS without contraindications to performing MRI, providing excellent inter-rater reliability for 7T MRI (ICC 0.97) yet poor reliability for 3T MRI (ICC 0.62). To our knowledge, test-retest reliability was never studied. CL are currently not applicable in real world due to low cost/time efficiency and availability of ultra-high-field MRI scanners, and are currently reserved for studies only.

### Paramagnetic rim lesions

Paramagnetic rim lesions (PRL), a biomarker of persistent chronic inflammation, axonal transection and therefore disease progression, were used as an outcome measure in 8 studies comprising 639 PwMS with 2 conducted in a real-world setting.

Quality of evidence for PRL as an outcome measure was rated as very low to low. PRL are currently not clinically useful. MDC was rarely studied (2–5.9), and CRC was studied only once (≥4). Hence, they have a limited sensitivity for change due to proximity/congruence of MDC and CRC. PRL are probably generalizable to all PwMS without contraindications to performing MRI, providing good to excellent inter-rater agreement (0.86–0.99), whereas test-retest reliability was, to our knowledge, never studied. PRL are currently not applicable in real world due to limited availability of the required MRI sequences, and therefore reserved for studies.

***Glial fibrillary acidic protein***

Glial fibrillary acidic protein (GFAP), one of the major intermediate cytoskeletal proteins of astrocytes and a well-established marker of reactive astrogliosis, was used as an outcome measure in 5 studies (3 _CSF_GFAP, 2 sGFAP) comprising 354 PwMS with 1 conducted in a real-world setting. Quality of evidence for GFAP as an outcome measure was rated as very low. GFAP is currently not clinically useful, with MDC being broad in CSF (282.2–535 pg/ml) but low variability in serum (64.5–74.7 pg/ml). CRC was only studied once in serum (79.2 pg/ml), and due to proximity/congruence of MDC and CRC, there is probable limited sensitivity and specificity for change. It is also limited by high costs and poor availability. GFAP is probably generalizable to all PwMS, with moderate to good test-retest reliability (ICC 0.76–0.81) and probably minimal inter-rater variability due to relatively standardized protocols for its detection. However, GFAP is currently not applicable in real world due to low cost/time efficiency, lack of availability, and still-to-be-explored clinical relevance. In that way, it is reserved for studies, with a need for standardization of measurement techniques, i.e., sample collection, assay methods, and well-defined diagnostic and prognostic cut-off values.

## *Measurable patient reported outcomes*

Supplementary Table 5 provides a detailed summary of all PROs assessed. As this systematic literature review includes PROs that have been used as endpoints in clinical trials, we regard evidence and reports for the MDC and CRC to be of utmost importance. More than 90 percent of the studies investigated did not specifically specify these benchmarks. Overall, authors stated the MDC for 10 PROs and CRC for 45 PROS. Therefore, we can only identify a very few PROs that may have the potential to be clinically useful. Much less, no PRO met the requirement to be regarded as a core measure.

Here, the PROs not described in the main manuscript are discussed.

### *Health related quality of life (HrQoL) – targeted measures for HRQoL specific to MS*

The MSQoL-54 (Multiple Sclerosis Quality of Life-54 Instrument) is a 54-item questionnaire derived from the generic SF-36 instrument. It has been used in 35 studies on 3,268 pwMS. Internal consistency reliability estimates for the 12 multi-item scales ranged from 0.75 to 0.96 In a sample of 179 pwMS. Test-retest intraclass correlation coefficients ranged from 0.66 to 0.96. Only 1 study reported the CDC to be at 2.5 points. There is no established evidence on CRC or MDC, thus, usefulness is less established compared to other targeted HRQoL-measures in pwMS [2]. It takes longer to complete (10-20 minutes) compared to other measures, which could be an obstacle in establishing usefulness for clinical routine.

### Health related quality of life (HrQoL) – generic measures for HRQoL not specific to MS

The EuroQol (EQ-5D) is a generic health index reporting on the current clinical status. It is the most widely used generic HRQoL PRO in our sample and it has been used in 41 studies on 15,426 pwMS. It has the shortest completion time of all generic HRQoL measures. There is no robust evidence on CRC and MDC, thus, its usefulness is less established compared to targeted HRQoL measures in pwMS.

The SF-36 is a generic HRQoL questionnaire and it has been used in 42 studies on 6,851 PwMS. It reports on eight different domains: vitality, physical functioning, bodily pain, general health perceptions, physical role functioning, emotional role functioning, social role functioning, mental health or emotional wellbeing. It generates a physical and mental subscore. It is likely not applicable in clinical routine as it takes a considerable amount of time to complete (15-20min). There is some evidence indicating CRC at ≥5 points.

Based on these findings, it seems more appropriate to relate to targeted rather than generic PROs in MS.

### *Depression, Anxiety and Mental Health*

The Beck Depression Inventory (BDI) is a 21-item questionnaire investigating the severity of depression [5]. In our sample we found 35 studies reporting its use with 5,416 pwMS. Two studies mention CRC (5 points; 20%). Completion time is similar to the HADS (5-10mins). It had not been developed specifically for pwMS, yet, its usefulness is somewhat established. BDI is likely generalizable to detect depression. Out of one study and one meta-analysis examining internal consistency, both reported excellent internal consistency. One study examining test-retest reliability and reported adequate test-retest properties. The BDI is applicable in a real-world setting as it is easy to use and it has the potential to be digitalized. Its completion time (5-10min) is similar to other measures for depression. Even though the lack of evidence for MDC and CRC currently limit its usefulness, we would still classify it as an aspirational outcome measure. However, in comparison to HADS, BDI measures only depression. Based on the broad range of studies it has been used in, it has the potential to demonstrate possible changes in clinical depression for pwMS over time.

### *Fatigue*

The Fatigue Severity Scale (FSS) is a 9-item questionnaire that measures fatigue severity. Higher scores for each item indicate greater fatigue severity. Its initial validation was for people with MS and systemic lupus erythematosus [6]. As a brief self-report of fatigue severity, the FSS yields acceptable internal consistency, stability over time, and sensitivity to clinical changes. The studies investigated in our sample (44 studies, 8,558 pwMS) with only one reports on MDC and none on CRC. The FSS is the most broadly used fatigue scale in pwMS and takes the shortest amount of time to complete. The FSS is applicable in a real-world setting as it is easy to use, takes a few minutes to complete (<5min) and has the potential to be digitalized. Even though the lack of evidence for MDC and CRC currently strongly limit its usefulness, we would still classify it as an aspirational outcome measure as efforts have been made to validate this PRO for pwMS. Based on the broad range of studies it has been used in, it has the potential to demonstrate possible changes in fatigue severity for pwMS over time.

The Modified Fatigue Impact Scale (MFIS) is a 21-item questionnaire validated to assess fatigue in pwMS generating 3 separate subscores (physical, cognitive, psychosocial). It has been used in 68 studies on 6,237 pwMS. It was comprised by a consortium in 1998, in our sample there were some reports on MDC (7-8 points) and CRC (7-10 points).

The MFIS is a more elaborate fatigue scale in pwMS that takes slightly longer time to complete (5-10minutes). MFIS is applicable in a real-world setting as it is easy to use and it has the potential to be digitalized. With its three subscales it generates a more granular understanding of fatigue bearing the risk of unbalanced psychometric properties, particularly of the psychosocial subscale. Even though the lack of evidence for MDC and CRC strongly limit its usefulness, we would still classify it as an aspirational outcome measure as efforts have been made to validate this PRO for pwMS.

The Patient-Reported Outcomes Measurement Information System (PROMIS) assesses

Standardizing fatigue measurement in multiple sclerosis: the validity,

responsiveness and score interpretation of the PROMIS SF v1.0 – Fatigue

(MS) 8a

The PROMIS Fatigue (MS) 8a was developed as a measure of fatigue

experience and impacts over 7 days, in PwMS. The short form’s eight

items were derived from the PROMIS fatigue item bank based on input

from PwMS and clinical experts (Cook et al., 2012). The PROMIS Fatigue

(MS) 8a is scored on a T-score metric, which has a mean of 50 and a

standard deviation (SD) of 10; higher scores indicate higher fatigue. The

T-score metric is referenced to the US general population with respect to

race/ethnicity, age, education, and sex; for example, a T-score of 40

would be one SD below the US general population.

### *Work-related*

The Work Productivity and Activity Impairment (WPAI) questionnaire in 6 questions inquires the numbers of days and hours missed from work, days and hours worked, days during which performing work was difficult and the extent to which the individual was limited at work (work impairment) during the past 7 days [7]. We found 12 studies using the WPAI in 4,154 pwMS. Test-retest reliability was good (0.7-0.9). We did not find reports on CRC and MDC for pwMS.

Based on current evidence, we currently do not regard the WPAI to be ready for clinical and routine practice. To establish usefulness, this PRO should be studied and validated in large cohorts of pwMS at a broad range of age and amount of disability. However, it can be useful in studies with distinct endpoints or questions (i.e. working abilities).

## *Combinations of measurable outcomes*

Supplementary Table 6 provides a detailed summary of all combination of measurable outcomes assessed. The Supplemental Results Section discusses the those not described here.

Here, the combination not described in the main manuscript are discussed.

### *No evidence of disease activity with 4 dimensions*

No evidence of disease activity with 4 dimensions (NEDA-4), a composite measure of relapses, EDSS worsening, MRI activity and brain atrophy, was used as an outcome measure in 5 studies (in none as a primary endpoint) on 699 pwMS, with three studies undertaken in a real-world setting. The overall quality of evidence for NEDA-4 as an outcome measure was graded as very low to low. NEDA-4 retains all the limitations and varying definitions of NEDA-3 and its composites. There is also no consensus on the definition of “brain atrophy” in NEDA-4 with definitions comprising annualized ≥0.4% of whole brain atrophy (3 studies with varying MRI intervals), “no enlargement of third ventricle volume” (1 study) or no increased thalamic atrophy (z-score ≤-1.645, 1 study). NEDA-4 can be theoretically generalized to all patients with relapsing MS (but not PPMS), access to regular MRI follow-up investigations. External validity in terms of test-retest reliability has not been formally studied and is likely strongly limited by the inter-rater variability of EDSS, more importantly MRI activity, and most importantly brain atrophy. NEDA-4 is not validated as a surrogate marker of treatment response. As for NEDA-3, MDC and CRC are congruent by definition with subsequently very low sensitivity and specificity for change over time. Loss of NEDA-4 is likely driven mostly by MRI activity and/or brain atrophy, inducing imbalance to the composite measure. Thus, NEDA-4 is neither clinically useful nor applicable in real-world as brain atrophy is not available, and even if offered, not sufficiently valid. Currently, NEDA-4 can be only considered as an additional outcome measure reserved for studies in centers, which can provide all these prerequisites.

Steps for advancing NEDA-4 to an aspirational outcome measure are similar to those outlined above for NEDA-3.

### *No evidence of progression with 3 dimensions*

No evidence of progression with 3 dimensions (NEP-3) sometimes also termed “EDSS plus”, a composite measure of EDSS, T25FW and 9HPT, was used as an outcome measure in 14 studies (in 1 as a primary endpoint) on 6,689 pwMS, with three studies undertaken in a real-world setting. The overall quality of evidence for NEP-3 as an outcome measure was graded as moderate to high. In the identified studies, NEP-3 definitions included 3 different definitions of EDSS worsening, while T25FW and 9HPT worsening were consistently defined as ≥20%. As every composite measure, NEP-3 retains all the limitations of its ingredients (EDSS, T25FW and 9HPT as discussed above). MDC and CRC of NEP-3 have not been formally investigated, but due to the dichotomous definition of the three outcomes, MDC and CRC are congruent and, consequently, sensitivity and specificity for change is low and requires confirmation over time, especially in RMS where spontaneous fluctuations are more likely to occur.

NEP-3 is generalizable to all pwMS with walking ability (EDSS 0-6.5). External validity of NEP-3 has not been formally studied but is likely moderate to good based on results for EDSS, T25FW and 9HPT, with no relevant inter-rater variability or practice effects.

NEP-3 is likely applicable in real-world setting due to the moderate additional administration time (2-5 minutes for T25FW and 9HPT) and as it can be obtained by trained staff as well as the treating neurologist.

Hence, NEP-3 is likely clinically useful in assessing disability progression over time and deemed as an aspirational outcome measure for all pwMS with retained walking ability (EDSS 0-6.5), especially in those with PMS or suspicion of imminent PMS.

Open knowledge gaps regard the confirmation and refinement of MDC/CRC in larger cohorts with MS populations comprising a wider scope of age and level of disability, but also gender and ethnicity. Investigating whether a further differentiation beyond the dichotomization of a patient remaining free of EDSS/T25FW/9HPT worsening or not, would provide additional valuable information. Advancement of digital and smart solutions regarding administration of the T25FW and 9HPT with a focus on standardization and broad applicability are important technology gaps.

### *Multiple Sclerosis Functional Composite*

The Multiple Sclerosis Functional Composite (MSFC), a composite of T25FW, 9HPT and PASAT, was employed as an outcome measure in 9 studies (in 1 as a primary endpoint) on 2,073 pwMS, with one study undertaken in a real-world setting.

Quality of evidence for MSFC as an outcome measure was rated as moderate to high. MDC of MSFC has not been formally studied, but can be extrapolated for T25FW (≥12-20%) and 9HPT (≥18-29%) but not for PASAT (no studies available). CRC for MSFC as a composite has been rarely studied with results ranging from ≥10-20%. As a consequence, data is insufficient to conduct a valid assessment of sensitivity and specificity for change, although it is likely low.

The MSFC is generalizable to pwMS with walking ability (EDSS 0-6.5) and without severe cognitive impairment. Concerning external validity, test-retest reliability is excellent with no relevant inter-rater variability, but there is a considerable practice effect for PASAT and therefore also for MSFC. The MSFC is considerably time consuming (administration time 20-25 minutes), mainly owed to the PASAT (10-15minutes) and does not contribute information on health utility independent of EDSS. Therefore, MSFC is currently neither useful nor applicable as an outcome measure in routine clinical practice.

Open knowledge gaps concern data on MDC/CRC in large cohorts with different MS populations and age groups (including assessment of practice effects depending on intervals) and analyses of clinical usefulness and ecological validity.

If clinical usefulness was shown, the MSFC would be suitable for digitalization and self-application by the patient, wherefore advancement of digital and smart solutions with a focus on standardization and broad applicability would be technology gaps.

### *Virtual composite measures*

The current state of evidence and knowledge/technology gaps regarding virtual composite measures such as the Floodlight® or the DREAMS® Applications can be summarized as there are no studies available on MDC and CRC of MSPT, which is why sensitivity and specificity for change cannot be assessed currently.

Virtual composite measures would be generalizable to most pwMS with walking ability (EDSS 0-6.5) and without severe cognitive or visual impairment as well as access/willingness/ability to perform these tests on an electronic device. External validity regarding test-retest reliability is currently not sufficiently studied, while no relevant inter-rater variability is expected. Applicability in real world is strongly dependent on availability of electronic devices as well as data collection and software solutions enabling storage, visualization and interpretation of results.

Currently, virtual composite measures are neither useful nor applicable as an outcome measure in routine clinical practice but could be useful in studies or very specific settings. Open knowledge gaps remain regarding investigation of MDC and CRC in larger cohorts with MS populations comprising a wider scope of age and level of disability. Similarly, there is a need for analyses of clinical usefulness and ecological validity. Technology gaps comprise development of data collection and software solutions enabling storage, visualization and interpretation of results as discussed.

# Supplemental References

[1] Goldman MD, LaRocca NG, Rudick RA, et al. Evaluation of multiple sclerosis disability outcome measures using pooled clinical trial data. *Neurology* 2019; 93: 10.1212/WNL.0000000000008519.

[2] Vickrey BG, Hays RD, Harooni R, et al. A health-related quality of life measure for multiple sclerosis. *Qual Life Res* 1995; 4: 187–206.

[3] Kamudoni P, Johns J, Cook KF, et al. A comparison of the measurement properties of the PROMIS Fatigue (MS) 8a against legacy fatigue questionnaires. *Mult Scler Relat Disord* 2022; 66: 104048.

[4] Kamudoni P, Amtmann D, Johns J, et al. The validity, responsiveness, and score interpretation of the PROMISnq Physical Function – Multiple Sclerosis 15a short form in multiple sclerosis. *Mult Scler Relat Disord* 2022; 62: 103753.

[5] Beck AT, Steer RA, Carbin MG. Psychometric properties of the Beck Depression Inventory: Twenty-five years of evaluation. *Clin Psychol Rev* 1988; 8: 77–100.

[6] Krupp LB, LaRocca NG, Muir-Nash J, et al. The Fatigue Severity Scale: Application to Patients With Multiple Sclerosis and Systemic Lupus Erythematosus. *Arch Neurol-chicago* 1989; 46: 1121–1123.

[7] Reilly MC, Zbrozek AS, Dukes EM. The Validity and Reproducibility of a Work Productivity and Activity Impairment Instrument. *Pharmacoeconomics* 1993; 4: 353–365.
