# Supplementary material for: Treating to target in multiple sclerosis: Do we know how to measure whether we hit it?
Source: Eur J Neurol. 2024 Oct 24;31(12):e16526. doi: 10.1111/ene.16526 (PMC11554867; doi:10.1111/ene.16526)
Supplement: Supplementary file 2 — Data S2. [file ENE-31-e16526-s002.docx]

# Supplemental Methodology

The objective of this systematic literature review (SLR) was to identify current and potential measurable disease-modifying treatment outcome targets in multiple sclerosis (MS) with the specific goal to focus on utility and implementability in clinical practice in order to improve quality of care in MS.

The SLR was conducted according to the PRISMA 2020 methodology [8].

## Search strategy and study selection

The literature search for the proposed SLR was conducted in the databases of MEDLINE, EMBASE, and the Cochrane library. The main search terms comprised “Multiple sclerosis“ and “outcome”. Additional search terms (“endpoint”, “treatment”, “target”, “patient-reported outcome”, “PRO”, “biomarker”, “measurable”) were combined with “multiple sclerosis” deploying Boole’s operators.

*Eligibility criteria* comprised any study reporting original data or any systematic review, which:

1. Were conducted in a population of persons with MS without any limitations or selections regarding MS subtype, MS disease course or age (thus also including neuropediatric MS)
2. Report any outcome viewed as a potential treatment target
3. Have been published between January 1^st^ 2017 and August 31^st^ 2022
4. Are published in English
5. Are published from any geographical area

Relevant articles were added by scanning the references of found articles (backward search) and locating newer articles that included the original cited paper (forward search).

*Criteria for exclusion* of studies from the SLR were defined as

1. Studies conducted in a population other than persons with MS, i.e. healthy persons, patients with diseases other than MS
2. Studies reporting of no outcomes viewed as a potential treatment target
3. In vitro/animal studies, non-systematic reviews, letters, editorials, expert/opinion papers, case reports, etc.
4. Publications before January 1^st^ 2017
5. Publications in any language other than English

The study selection process was designed as a two-step identification of evidence by conducting 1) a title/abstract screening, and 2) a full-text screening of the studies identified in the title/abstract screening. In accordance with PRISMA 2020 methodology, each step of the search process and the study identification/inclusion/exclusion is transparently documented in a flow-chart (Figure 1) and a screening list (Supplementary Table 1 including all relevant information (outcomes reported in the respective study, full study title, first study author, journal, date of publication and full-text link) [8].

All MS treatment outcomes identified in the included studies were then grouped according to the following four groups:

1. Objectively measurable clinical outcomes
2. Objectively measurable paraclinical outcomes
3. Measurable patient-reported outcomes
4. Any combination(s) 1-3

## *Grading of evidence*

The methodological quality for each outcome was graded according to the quality of available evidence using the Grades of Recommendation, Assessment, Development, and Evaluation (GRADE) tool for best-evidence synthesis following four eye principle (authors GB/NK/PA and TB) [9, 10]. All studies included were systematically analyzed regarding several key criteria (study design, sample size, study population, minimal detectable change (MDC), clinically relevant change (CRC), external and ecological validity, sources of bias), separately assessed for each MS outcome. In accordance with PRISMA 2020 methodology, each step of the grading process was transparently documented in a Microsoft Excel® File (“Full study lists”, Supplemental Table 2) including all relevant information (study design, sample size, study population, MDC, CRC, sources of bias) [8].

### *Study design and population*

Study design was categorized as 1) either retrospective or prospective, 2) either randomized, open or observational, and 3) within a distinct study setting or clinical routine.

Each respective MS outcome in each study was classified as either the primary, secondary or an exploratory outcome within each study. The available sample size was retrieved, also separately for each MS outcome. The study population was characterized for each study and each MS outcome regarding range of age and included subtypes of MS (relapsing MS, progressive MS) as well as level of disability.

### *Minimal detectable change/ clinically relevant change*

Each MS outcome in every study was analyzed regarding the applied definition or achieved results of what constitutes MDC and CRC.

### *Bias*

Potential sources of bias were evaluated according to the PRISMA 2020 methodology [8]:

- No serious limitations (no downgrade): Low risk of bias for all key criteria
- Serious limitations (downgrade one level): Crucial limitation for one criterion or some limitations for multiple criteria sufficient to lower confidence in the estimate of effect.
- Very serious limitations (downgrade two levels): Crucial limitation for one or more criteria sufficient to substantially lower one’s confidence in the estimate of effect.
