# Supplementary material for: Treating to target in multiple sclerosis: Do we know how to measure whether we hit it?
Source: Eur J Neurol. 2024 Oct 24;31(12):e16526. doi: 10.1111/ene.16526 (PMC11554867; doi:10.1111/ene.16526)
Supplement: Supplementary file 3 — Data S3. [file ENE-31-e16526-s001.docx]

# **Supplemental: Complete List of References**

[1] Compston A, Coles A. Multiple sclerosis. *Lancet* 2002; 359: 1221–1231.

[2] Smolen JS, Landewé RBM, Bergstra SA, et al. EULAR recommendations for the management of rheumatoid arthritis with synthetic and biological disease-modifying antirheumatic drugs: 2022 update. *Ann Rheum Dis* 2023; 82: 3–18.

[3] Yeung J, Bourcier M, Gooderham MJ, et al. Management of moderate‐to‐severe plaque psoriasis with biologics: A treat‐to‐target position paper. *Dermatol Ther* 2022; 35: e15777.

[4] Lublin FD, Reingold SC. Defining the clinical course of multiple sclerosis. *Neurology* 1996; 46: 907-911.

[5] Bielekova B, Kadom N, Fisher E, et al. MRI as a marker for disease heterogeneity in multiple sclerosis. *Neurology* 2005; 65: 1071–1076.

[6] Lucchinetti C, Brück W, Parisi J, et al. Heterogeneity of multiple sclerosis lesions: Implications for the pathogenesis of demyelination. *Ann Neurol* 2000; 47: 707–717.

[7] Group BDW. Biomarkers and surrogate endpoints: Preferred definitions and conceptual framework. *Clin Pharmacol Ther* 2001; 69: 89–95.

[8] Page MJ, McKenzie JE, Bossuyt PM, et al. The PRISMA 2020 statement: an updated guideline for reporting systematic reviews. *Syst Rev* 2021; 10: 89.

[9] Balshem H, Helfand M, Schünemann HJ, et al. GRADE guidelines: 3. Rating the quality of evidence. *J Clin Epidemiol* 2011; 64: 401–406.

[10] Guyatt GH, Oxman AD, Vist G, et al. GRADE guidelines: 4. Rating the quality of evidence—study limitations (risk of bias). *J Clin Epidemiol* 2011; 64: 407–415.

[11] Lanzillo R, Carotenuto A, Moccia M, et al. A longitudinal real‐life comparison study of natalizumab and fingolimod. *Acta Neurol Scand* 2017; 136: 217–222.

[12] Ozakbas S, Cinar BP, Kosehasanoğullari G, et al. Monthly methylprednisolone in combination with interferon beta or glatiramer acetate for relapsing-remitting multiple sclerosis: A multicentre, single-blind, prospective trial. *Clin Neurol Neurosur* 2017; 160: 69–72.

[13] Kalincik T, Manouchehrinia A, Sobisek L, et al. Towards personalized therapy for multiple sclerosis: prediction of individual treatment response. *Brain* 2017; 140: 2426–2443.

[14] Lorscheider J, Jokubaitis VG, Spelman T, et al. Anti-inflammatory disease-modifying treatment and short-term disability progression in SPMS. *Neurology* 2017; 89: 1050–1059.

[15] Havrdova E, Arnold DL, Cohen JA, et al. Alemtuzumab CARE-MS I 5-year follow-up: Durable efficacy in the absence of continuous MS therapy. *Neurology* 2017; 89: 1107–1116.

[16] Lanzillo R, Carbone F, Quarantelli M, et al. Immunometabolic profiling of patients with multiple sclerosis identifies new biomarkers to predict disease activity during treatment with interferon beta-1a. *Clin Immunol* 2017; 183: 249–253.

[17] Diaz-Cruz C, Chua AS, Malik MT, et al. The effect of alcohol and red wine consumption on clinical and MRI outcomes in multiple sclerosis. *Mult Scler Relat Dis* 2017; 17: 47–53.

[18] Jesus-Ribeiro J, Correia I, Martins AI, et al. Pregnancy in Multiple Sclerosis_ A Portuguese cohort study. *Mult Scler Relat Dis* 2017; 17: 63–68.

[19] Lau AY, Ip W, Au C, et al. Prevalence of neutralising antibodies to interferon-beta and clinical response in Chinese patients with relapsing multiple sclerosis. *Multiple Scler J - Exp Transl Clin* 2017; 3: 2055217317733485.

[20] Bisgaard A, Pihl-Jensen G, Frederiksen JL. The neutrophil-to-lymphocyte ratio as disease actvity marker in multiple sclerosis and optic neuritis. *Mult Scler Relat Dis* 2017; 18: 213–217.

[21] Lublin FD, Cofield SS, Cutter GR, et al. Long-term follow-up of a randomized study of combination interferon and glatiramer acetate in multiple sclerosis: Efficacy and safety results up to 7 years. *Mult Scler Relat Dis* 2017; 18: 95–102.

[22] Cohen M, Brochet B, Clavelou P, et al. Cognition and quality of life in clinically isolated syndrome patients starting a disease modifying therapy in the QUALICIS study may not predict treatment response at one year. *J Neurol Sci* 2017; 382: 73–78.

[23] Selmaj K, Barkhof F, Belova AN, et al. Switching from branded to generic glatiramer acetate: 15-month GATE trial extension results. *Multiple Scler Houndmills Basingstoke Engl* 2017; 23: 1909–1917.

[24] Alroughani R, Ahmed SF, Behbehani R, et al. Effectiveness and Safety of Dimethyl Fumarate Treatment in Relapsing Multiple Sclerosis Patients: Real-World Evidence. *Neurology Ther* 2017; 6: 189–196.

[25] Comi G, Patti F, Rocca MA, et al. Efficacy of fingolimod and interferon beta-1b on cognitive, MRI, and clinical outcomes in relapsing–remitting multiple sclerosis: an 18-month, open-label, rater-blinded, randomised, multicentre study (the GOLDEN study). *J Neurol* 2017; 264: 2436–2449.

[26] Ziemssen T, Lang M, Tackenberg B, et al. Clinical and Demographic Profile of Patients Receiving Fingolimod in Clinical Practice in Germany and the Benefit–Risk Profile of Fingolimod After 1 Year of Treatment: Initial Results From the Observational, Noninterventional Study PANGAEA. *Neurotherapeutics* 2018; 15: 190–199.

[27] Azary S, Schreiner T, Graves J, et al. Contribution of dietary intake to relapse rate in early paediatric multiple sclerosis. *J Neurology Neurosurg Psychiatry* 2018; 89: 28.

[28] Moccia M, Palladino R, Carotenuto A, et al. A 8-year retrospective cohort study comparing Interferon-β formulations for relapsing‐remitting multiple sclerosis. *Mult Scler Relat Dis* 2018; 19: 50–54.

[29] Zecca C, Merlini A, Disanto G, et al. Half-dose fingolimod for treating relapsing-remitting multiple sclerosis: Observational study. *Mult Scler J* 2017; 24: 167–174.

[30] Guger M, Enzinger C, Leutmezer F, et al. Real‐life clinical use of natalizumab and fingolimod in Austria. *Acta Neurol Scand* 2018; 137: 181–187.

[31] Signoriello E, Landi D, Monteleone F, et al. Fingolimod reduces the clinical expression of active demyelinating lesions in MS. *Mult Scler Relat Dis* 2018; 20: 215–219.

[32] Petersen ER, Oturai AB, Koch-Henriksen N, et al. Smoking affects the interferon beta treatment response in multiple sclerosis. *Neurology* 2018; 90: e593–e600.

[33] Río J, Rovira À, Tintoré M, et al. Disability progression markers over 6–12 years in interferon-β-treated multiple sclerosis patients. *Mult Scler J* 2017; 24: 322–330.

[34] Granqvist M, Boremalm M, Poorghobad A, et al. Comparative Effectiveness of Rituximab and Other Initial Treatment Choices for Multiple Sclerosis. *Jama Neurol* 2018; 75: 1–8.

[35] Koda T, Namba A, Nakatsuji Y, et al. Beneficial effects of fingolimod in MS patients with high serum Sema4A levels. *Plos One* 2018; 13: e0193986.

[36] Fleischer V, Friedrich M, Rezk A, et al. Treatment response to dimethyl fumarate is characterized by disproportionate CD8+ T cell reduction in MS. *Mult Scler J* 2017; 24: 632–641.

[37] Esposito F, Ferrè L, Clarelli F, et al. Effectiveness and baseline factors associated to fingolimod response in a real-world study on multiple sclerosis patients. *J Neurol* 2018; 265: 896–905.

[38] Kocsik AS, Klein DE, Liedke M, et al. Induction of disease remission with one cycle of alemtuzumab in relapsing–remitting MS. *J Neurol* 2018; 265: 1226–1229.

[39] Zecca C, Roth S, Findling O, et al. Real‐life long‐term effectiveness of fingolimod in Swiss patients with relapsing‐remitting multiple sclerosis. *Eur J Neurol* 2018; 25: 762–767.

[40] D’Amico E, Patti F, Zanghì A, et al. Lateral switch to IFN beta-1a 44 mcg may be effective as escalation switch to fingolimod in selected persons with relapsing remitting multiple sclerosis: a real-world setting experience. *Expert Rev Clin Phar* 2018; 11: 531–536.

[41] Cohan SL, Moses H, Calkwood J, et al. Clinical outcomes in patients with relapsing-remitting multiple sclerosis who switch from natalizumab to delayed-release dimethyl fumarate: A multicenter retrospective observational study (STRATEGY). *Mult Scler Relat Dis* 2018; 22: 27–34.

[42] Lorscheider J, Benkert P, Lienert C, et al. Comparative analysis of natalizumab versus fingolimod as second-line treatment in relapsing–remitting multiple sclerosis. *Mult Scler J* 2018; 24: 777–785.

[43] Scotti B, Disanto G, Sacco R, et al. Effectiveness and safety of Rituximab in multiple sclerosis: an observational study from Southern Switzerland. *Plos One* 2018; 13: e0197415.

[44] Smoot K, Spinelli KJ, Stuchiner T, et al. Three-year clinical outcomes of relapsing multiple sclerosis patients treated with dimethyl fumarate in a United States community health center. *Mult Scler J* 2017; 24: 942–950.

[45] Spelman T, Frisell T, Piehl F, et al. Comparative effectiveness of rituximab relative to IFN-β or glatiramer acetate in relapsing-remitting MS from the Swedish MS registry. *Multiple Sclerosis Journal* 2018; 24: 1087–1095.

[46] Prosperini L, Lucchini M, Haggiag S, et al. Fingolimod vs dimethyl fumarate in multiple sclerosis: A real-world propensity score-matched study. *Neurology* 2018; 91: e153–e161.

[47] Vollmer B, Honce JM, Sillau S, et al. The impact of very short transition times on switching from Natalizumab to Fingolimod on imaging and clinical effectiveness outcomes in multiple sclerosis. *J Neurol Sci* 2018; 390: 89–93.

[48] Casanova B, Lacruz L, Villar ML, et al. Different clinical response to interferon beta and glatiramer acetate related to the presence of oligoclonal IgM bands in CSF in multiple sclerosis patients. *Neurol Sci* 2018; 39: 1423–1430.

[49] Mallucci G, Annovazzi P, Miante S, et al. Two-year real-life efficacy, tolerability and safety of dimethyl fumarate in an Italian multicentre study. *J Neurol* 2018; 265: 1850–1859.

[50] Defer G, Seze J de, Bouee S, et al. Outcomes and treatment management of a French cohort suffering from multiple sclerosis: A retrospective epidemiological study. *Mult Scler Relat Dis* 2018; 25: 276–281.

[51] Traboulsee AL, Machan L, Girard JM, et al. Safety and efficacy of venoplasty in MS: A randomized, double-blind, sham-controlled, phase II trial. *Neurology* 2018; 91: 10.1212/WNL.0000000000006423.

[52] Fakih R, Matiello M, Chitnis T, et al. Efficacy and safety of mycophenolate mofetil in progressive multiple sclerosis patients. *J Neurol* 2018; 265: 2688–2694.

[53] Cohan S, Smoot K, Kresa-Reahl K, et al. Outcomes of Stable Multiple Sclerosis Patients Staying on Initial Interferon Beta Therapy Versus Switching to Another Interferon Beta Therapy: A US Claims Database Study. *Adv Ther* 2018; 35: 1894–1904.

[54] Pakdaman H, Abbasi M, Gharagozli K, et al. A randomized double-blind trial of comparative efficacy and safety of Avonex and CinnoVex for treatment of relapsing-remitting multiple sclerosis. *Neurol Sci* 2018; 39: 2107–2113.

[55] Prosperini L, Annovazzi P, Boffa L, et al. No evidence of disease activity (NEDA-3) and disability improvement after alemtuzumab treatment for multiple sclerosis: a 36-month real-world study. *J Neurol* 2018; 265: 2851–2860.

[56] Group NS, Braune S, Grimm S, et al. Comparative effectiveness of delayed-release dimethyl fumarate versus interferon, glatiramer acetate, teriflunomide, or fingolimod: results from the German NeuroTransData registry. *J Neurol* 2018; 265: 2980–2992.

[57] Kresa-Reahl K, Repovic P, Robertson D, et al. Effectiveness of Delayed-release Dimethyl Fumarate on Clinical and Patient-reported Outcomes in Patients With Relapsing Multiple Sclerosis Switching From Glatiramer Acetate: RESPOND, a Prospective Observational Study. *Clin Ther* 2018; 40: 2077–2087.

[58] Huppke P, Huppke B, Ellenberger D, et al. Therapy of highly active pediatric multiple sclerosis. *Mult Scler J* 2017; 25: 72–80.

[59] Zivadinov R, Medin J, Khan N, et al. Impact of fingolimod on clinical and magnetic resonance imaging outcomes in routine clinical practice: A retrospective analysis of the multiple sclerosis, clinical and MRI outcomes in the USA (MS-MRIUS) study. *Mult Scler Relat Dis* 2019; 27: 65–73.

[60] Ontaneda D, Nicholas J, Carraro M, et al. Comparative effectiveness of dimethyl fumarate versus fingolimod and teriflunomide among MS patients switching from first-generation platform therapies in the US. *Mult Scler Relat Dis* 2019; 27: 101–111.

[61] Pfeuffer S, Schmidt R, Straeten FA, et al. Efficacy and safety of alemtuzumab versus fingolimod in RRMS after natalizumab cessation. *J Neurol* 2018; 354: 899–9.

[62] Ayoobi F, Moghadam-Ahmadi A, Amiri H, et al. Achillea millefolium is beneficial as an add-on therapy in patients with multiple sclerosis: A randomized placebo-controlled clinical trial. *Phytomedicine* 2019; 52: 89–97.

[63] Chalmer TA, Kalincik T, Laursen B, et al. Treatment escalation leads to fewer relapses compared with switching to another moderately effective therapy. *J Neurol* 2019; 266: 306–315.

[64] Kobelt G, Jönsson L, Pavelcova M, et al. Real-Life Outcome in Multiple Sclerosis in the Czech Republic. *Multiple Scler Int* 2019; 2019: 7290285.

[65] Baroncini D, Zaffaroni M, Moiola L, et al. Long-term follow-up of pediatric MS patients starting treatment with injectable first-line agents: A multicentre, Italian, retrospective, observational study. *Mult Scler J* 2018; 25: 399–407.

[66] Condé S, Moisset X, Pereira B, et al. Dimethyl fumarate and teriflunomide for multiple sclerosis in a real‐life setting: a French retrospective cohort study. *Eur J Neurol* 2019; 26: 460–467.

[67] Ziemssen T, Lang M, Tackenberg B, et al. Real-world persistence and benefit–risk profile of fingolimod over 36 months in Germany. *Neurology - Neuroimmunol Neuroinflammation* 2019; 6: e548.

[68] Mariottini A, Innocenti C, Forci B, et al. Safety and efficacy of autologous hematopoietic stem‐cell transplantation following natalizumab discontinuation in aggressive multiple sclerosis. *Eur J Neurol* 2019; 26: 624–630.

[69] Buron MD, Chalmer TA, Sellebjerg F, et al. Comparative effectiveness of teriflunomide and dimethyl fumarate: A nationwide cohort study. *Neurology* 2019; 92: e1811–e1820.

[70] Evdoshenko E, Stepanova A, Shumilina M, et al. Real-world study of efficacy, risk management and reasons for discontinuation of natalizumab for treatment of multiple sclerosis in Russia. *Plos One* 2019; 14: e0217303.

[71] Buratti L, Iacobucci DE, Viticchi G, et al. Sleep quality can influence the outcome of patients with multiple sclerosis. *Sleep Med* 2019; 58: 56–60.

[72] Perumal J, Fox RJ, Balabanov R, et al. Outcomes of natalizumab treatment within 3 years of relapsing-remitting multiple sclerosis diagnosis: a prespecified 2-year interim analysis of STRIVE. *Bmc Neurol* 2019; 19: 116.

[73] Boziki M, Lagoudaki R, Melo P, et al. Induction of apoptosis in CD4(+) T-cells is linked with optimal treatment response in patients with relapsing-remitting multiple sclerosis treated with Glatiramer acetate. *J Neurol Sci* 2019; 401: 43–50.

[74] Montalban X, Arnold DL, Weber MS, et al. Placebo-Controlled Trial of an Oral BTK Inhibitor in Multiple Sclerosis. *New Engl J Med* 2019; 380: 2406–2417.

[75] Hyun J-W, Shin H-J, Jang H, et al. Therapeutic Outcome of Alemtuzumab in Korean Patients with Multiple Sclerosis: 2-Year Follow-Up. *J Clin Neurol* 2019; 15: 328–333.

[76] Zecca C, Disanto G, Sacco R, et al. Use of glatiramer acetate between 2010–2015: effectiveness, safety and reasons to start GA as first or second line treatment in Swiss multiple sclerosis patients. *Bmc Neurol* 2019; 19: 159.

[77] Roquemaurel A de, Galli P, Landais A, et al. Fingolimod for the treatment of multiple sclerosis in French West Indies, a real-world study in patients from African ancestry. *J Neurol Sci* 2019; 402: 180–187.

[78] Boremalm M, Juto A, Axelsson M, et al. Natalizumab, rituximab and fingolimod as escalation therapy in multiple sclerosis. *Eur J Neurol* 2019; 26: 1060–1067.

[79] Camu W, Lehert P, Pierrot-Deseilligny C, et al. Cholecalciferol in relapsing-remitting MS: A randomized clinical trial (CHOLINE). *Neurology - Neuroimmunol Neuroinflammation* 2019; 6: e597.

[80] Laplaud D-A, Casey R, Barbin L, et al. Comparative effectiveness of teriflunomide vs dimethyl fumarate in multiple sclerosis. *Neurology* 2019; 93: 10.1212/WNL.0000000000007938.

[81] Frau J, Saccà F, Signori A, et al. Outcomes after fingolimod to alemtuzumab treatment shift in relapsing–remitting MS patients: a multicentre cohort study. *J Neurol* 2019; 266: 2440–2446.

[82] Yano H, Gonzalez C, Healy BC, et al. Discontinuation of disease-modifying therapy for patients with relapsing-remitting multiple sclerosis: Effect on clinical and MRI outcomes. *Multiple Sclerosis and Related Disorders* 2019; 35: 119–127.

[83] Guger M, Enzinger C, Leutmezer F, et al. Switching from natalizumab to fingolimod treatment in multiple sclerosis: real life data from the Austrian MS Treatment Registry. *J Neurol* 2019; 266: 2672–2677.

[84] Boz C, Terzi M, Özer B, et al. Comparative analysis of fingolimod versus teriflunomide in relapsing–remitting multiple sclerosis. *Mult Scler Relat Dis* 2019; 36: 101376.

[85] Barkhof F, Kappos L, Wolinsky JS, et al. Onset of clinical and MRI efficacy of ocrelizumab in relapsing multiple sclerosis. *Neurology* 2019; 93: e1778–e1786.

[86] Hupperts R, Smolders J, Vieth R, et al. Randomized trial of daily high-dose vitamin D 3in patients with RRMS receiving subcutaneous interferon β-1a. *Neurology* 2019; 10.1212/WNL.0000000000008445-12.

[87] Paolicelli D, Lucisano G, Manni A, et al. Retrospectively acquired cohort study to evaluate the long-term impact of two different treatment strategies on disability outcomes in patients with relapsing multiple sclerosis (RE.LO.DI.MS): data from the Italian MS Register. *J Neurol* 2019; 266: 3098–3107.

[88] Berger T, Brochet B, Brambilla L, et al. Effectiveness of delayed-release dimethyl fumarate on patient-reported outcomes and clinical measures in patients with relapsing–remitting multiple sclerosis in a real-world clinical setting: PROTEC. *Multiple Scler J - Exp Transl Clin* 2019; 5: 2055217319887191.

[89] Vollmer B, Ontaneda D, Harris H, et al. Comparative discontinuation, effectiveness, and switching practices of dimethyl fumarate and fingolimod at 36-month follow-up. *J Neurol Sci* 2019; 407: 116498.

[90] Clerico M, Mercanti SFD, Signori A, et al. Extending the Interval of Natalizumab Dosing: Is Efficacy Preserved? *Neurotherapeutics* 2020; 17: 200–207.

[91] Preziosa P, Rocca MA, Riccitelli GC, et al. Effects of Natalizumab and Fingolimod on Clinical, Cognitive, and Magnetic Resonance Imaging Measures in Multiple Sclerosis. *Neurotherapeutics* 2020; 17: 208–217.

[92] Chung KK, Altmann D, Barkhof F, et al. A 30‐Year Clinical and Magnetic Resonance Imaging Observational Study of Multiple Sclerosis and Clinically Isolated Syndromes. *Ann Neurol* 2020; 87: 63–74.

[93] Ceccarelli A, Mifsud V, Abusamra E, et al. Short term real-world Fingolimod efficacy and safety in Emirati patients with multiple sclerosis. *J Clin Neurosci* 2020; 71: 39–42.

[94] Diem L, Daponte A, Findling O, et al. Dimethyl fumarate vs fingolimod following different pretreatments: A retrospective study. *Neurology - Neuroimmunol Neuroinflammation* 2020; 7: e660.

[95] Ledinek AH, Jakob GB, Jerše J, et al. Intravenous immunoglobulins for the prevention of postpartum relapses in multiple sclerosis. *Mult Scler Relat Dis* 2020; 38: 101519.

[96] Lanzillo R, Moccia M, Palladino R, et al. Clinical predictors of Dimethyl Fumarate response in multiple sclerosis: a real life multicentre study. *Mult Scler Relat Dis* 2020; 38: 101871.

[97] Zanghì A, D’Amico E, Callari G, et al. Pregnancy and the Postpartum Period in Women With Relapsing-Remitting Multiple Sclerosis Treated With Old and New Disease-Modifying Treatments: A Real-World Multicenter Experience. *Front Neurol* 2020; 11: 105.

[98] Albert C, Mikolajczak J, Liekfeld A, et al. Fingolimod after a first unilateral episode of acute optic neuritis (MOVING) – preliminary results from a randomized, rater-blind, active-controlled, phase 2 trial. *Bmc Neurol* 2020; 20: 75.

[99] Ellwardt E, Rolfes L, Klein J, et al. Ocrelizumab initiation in patients with MS: A multicenter observational study. *Neurology - Neuroimmunol Neuroinflammation* 2020; 7: e719.

[100] Kopp TI, Blinkenberg M, Petersen T, et al. Long term effect of delayed treatment on disability in patients with paediatric onset multiple sclerosis: A prospective Danish cohort study. *Mult Scler Relat Dis* 2020; 40: 101956.

[101] Airas L, Nylund M, Mannonen I, et al. Rituximab in the treatment of multiple sclerosis in the Hospital District of Southwest Finland. *Mult Scler Relat Dis* 2020; 40: 101980.

[102] Butzkueven H, Kappos L, Wiendl H, et al. Long-term safety and effectiveness of natalizumab treatment in clinical practice: 10 years of real-world data from the Tysabri Observational Program (TOP). *J Neurology Neurosurg Psychiatry* 2020; 91: 660–668.

[103] Kopp TI, Blinkenberg M, Chalmer TA, et al. Predictors of treatment outcome in patients with paediatric onset multiple sclerosis. *Mult Scler J* 2019; 26: 964–975.

[104] Krysko KM, Graves JS, Rensel M, et al. Real‐World Effectiveness of Initial Disease‐Modifying Therapies in Pediatric Multiple Sclerosis. *Ann Neurol* 2020; 88: 42–55.

[105] Bowen J, Mehta R, Pelletier C, et al. Treatment Patterns Among Patients with Multiple Sclerosis Initiating Second-Line Disease-Modifying Therapy. *Adv Ther* 2020; 37: 3163–3177.

[106] Deslandes MQ, Alves PT, Alvarenga MP, et al. Effectiveness and Adverse Events of Use of Natalizumab in a Brazilian Cohort of Patients With Multiple Sclerosis. *Clin Ther* 2020; 42: 1292–1301.

[107] Guger M, Traxler G, Drabauer M, et al. Pregnancy Outcomes in Patients With Multiple Sclerosis Exposed to Natalizumab—A Retrospective Analysis From the Austrian Multiple Sclerosis Treatment Registry. *Front Neurol* 2020; 11: 676.

[108] Signori A, Saccà F, Lanzillo R, et al. Cladribine vs other drugs in MS: Merging randomized trial with real-life data. *Neurology - Neuroimmunol Neuroinflammation* 2020; 7: e878.

[109] Buron MD, Chalmer TA, Sellebjerg F, et al. Initial high-efficacy disease-modifying therapy in multiple sclerosis: A nationwide cohort study. *Neurology* 2020; 95: e1041–e1051.

[110] Ziemssen T, Bass AD, Berkovich R, et al. Efficacy and Safety of Alemtuzumab Through 9 Years of Follow-up in Patients with Highly Active Disease: Post Hoc Analysis of CARE-MS I and II Patients in the TOPAZ Extension Study. *Cns Drugs* 2020; 34: 973–988.

[111] Vollmer BL, Nair K, Sillau S, et al. Rituximab versus natalizumab, fingolimod, and dimethyl fumarate in multiple sclerosis treatment. *Ann Clin Transl Neur* 2020; 7: 1466–1476.

[112] Roos I, Leray E, Frascoli F, et al. Delay from treatment start to full effect of immunotherapies for multiple sclerosis. *Brain* 2020; 143: 2742–2756.

[113] Houtchens M, Bove R, Healy B, et al. MRI activity in MS and completed pregnancy: Data from a tertiary academic center. *Neurology - Neuroimmunol Neuroinflammation* 2020; 7: e890.

[114] Hauser SL, Kappos L, Arnold DL, et al. Five-years of ocrelizumab in relapsing multiple sclerosis: OPERA studies open-label extension. *Neurology* 2020; 95: 10.1212/WNL.0000000000010376.

[115] Zecca C, Bovis F, Novi G, et al. Treatment of multiple sclerosis with rituximab: A multicentric Italian–Swiss experience. *Mult Scler J* 2019; 26: 1519–1531.

[116] Boffa G, Lapucci C, Sbragia E, et al. Aggressive multiple sclerosis: a single‐centre, real‐world treatment experience with autologous haematopoietic stem cell transplantation and alemtuzumab. *Eur J Neurol* 2020; 27: 2047–2055.

[117] Hunter SF, Thomas FP, Cascione M, et al. Switching to fingolimod in PREFERMS: Effect of treatment history and naïvety on clinical, MRI and treatment satisfaction outcomes✰. *Mult Scler Relat Dis* 2020; 45: 102346.

[118] Oliveira ML, Lucchetta RC, Bonetti A de F, et al. Efficacy outcomes reported in trials of multiple sclerosis: a systematic scoping review. *Mult Scler Relat Dis* 2020; 45: 102435.

[119] Hersh CM, Harris H, Ayers M, et al. Effect of tobacco use on disease activity and DMT discontinuation in multiple sclerosis patients treated with dimethyl fumarate or fingolimod. *Multiple Scler J Exp Transl Clin* 2020; 6: 2055217320959815.

[120] Anderson V, Bentley E, Loveless S, et al. Serum neurofilament-light concentration and real-world outcome in MS. *J Neurol Sci* 2020; 417: 117079.

[121] Butzkueven H, Licata S, Jeffery D, et al. Natalizumab versus fingolimod for patients with active relapsing-remitting multiple sclerosis: results from REVEAL, a prospective, randomised head-to-head study. *Bmj Open* 2020; 10: e038861.

[122] Razaz N, Piehl F, Frisell T, et al. Disease activity in pregnancy and postpartum in women with MS who suspended rituximab and natalizumab. *Neurology Neuroimmunol Neuroinflammation* 2020; 7: e903.

[123] Damasceno A, Pimentel-Silva LR, Damasceno BP, et al. Exploring the performance of outcome measures in MS for predicting cognitive and clinical progression in the following years. *Mult Scler Relat Dis* 2020; 46: 102513.

[124] Chisari CG, Grimaldi LM, Salemi G, et al. Clinical effectiveness of different natalizumab interval dosing schedules in a large Italian population of patients with multiple sclerosis. *J Neurology Neurosurg Psychiatry* 2020; 91: 1297–1303.

[125] Fuente BP de la, Sabín J, Galán V, et al. Three-Year Effectiveness of Dimethyl Fumarate in Multiple Sclerosis: A Prospective Multicenter Real-World Study. *Cns Drugs* 2020; 34: 1275–1286.

[126] Yang C-C, Ro L-S, Tsai N-W, et al. Real-world evidence on the safety and effectiveness of fingolimod in patients with multiple sclerosis from Taiwan. *J Formos Med Assoc* 2021; 120: 542–550.

[127] Tsantes E, Curti E, Ferraro D, et al. Dimethyl fumarate‐induced lymphocyte count drop is related to clinical effectiveness in relapsing–remitting multiple sclerosis. *Eur J Neurol* 2021; 28: 269–277.

[128] Bovis F, Kalincik T, Lublin F, et al. Treatment response score to glatiramer acetate or interferon beta-1a. *Neurology* 2020; 10.1212/WNL.0000000000010991.

[129] Pavelek Z, Sobíšek L, Šarláková J, et al. Comparison of Therapies in MS Patients After the First Demyelinating Event in Real Clinical Practice in the Czech Republic: Data From the National Registry ReMuS. *Front Neurol* 2021; 11: 593527.

[130] Cheshmavar M, Mirmosayyeb O, Badihian N, et al. Rituximab and glatiramer acetate in secondary progressive multiple sclerosis: A randomized clinical trial. *Acta Neurol Scand* 2021; 143: 178–187.

[131] Fernandez‐Diaz E, Perez‐Vicente JA, Villaverde‐Gonzalez R, et al. Real‐world experience of ocrelizumab in multiple sclerosis in a Spanish population. *Ann Clin Transl Neur* 2021; 8: 385–394.

[132] Anderson A, Krysko KM, Rutatangwa A, et al. Clinical and Radiologic Disease Activity in Pregnancy and Postpartum in MS. *Neurology - Neuroimmunol Neuroinflammation* 2021; 8: e959.

[133] Ramos-Lopes J, Batista S, Barradas P, et al. Clinical effectiveness of reduced fingolimod dose in relapsing remitting multiple sclerosis—a Portuguese cohort. *Neurol Sci* 2021; 42: 1039–1043.

[134] Riancho J, Setien S, Torre JRS de la, et al. Does Extended Interval Dosing Natalizumab Preserve Effectiveness in Multiple Sclerosis? A 7 Year-Retrospective Observational Study. *Front Immunol* 2021; 12: 614715.

[135] Le M, Malpas C, Sharmin S, et al. Disability outcomes of early cerebellar and brainstem symptoms in multiple sclerosis. *Mult Scler J* 2020; 27: 755–766.

[136] Alroughani R, AlKawi Z, Hassan A, et al. Real-world retrospective study of effectiveness and safety of FINgOlimod in relapsing remitting multiple sclerosis in the Middle East and North Africa (FINOMENA). *Clin Neurol Neurosur* 2021; 203: 106576.

[137] Zanghì A, Gallo A, Avolio C, et al. Exit Strategies in Natalizumab-Treated RRMS at High Risk of Progressive Multifocal Leukoencephalopathy: a Multicentre Comparison Study. *Neurotherapeutics* 2021; 18: 1166–1174.

[138] Batista S, Nunes CC, Cerqueira JJ, et al. REALMS study: real-world effectiveness and safety of fingolimod in patients with relapsing-remitting multiple sclerosis in Portugal. *Neurol Sci* 2021; 42: 1995–2003.

[139] Buron MD, Kalincik T, Sellebjerg F, et al. Effect of lateral therapy switches to oral moderate-efficacy drugs in multiple sclerosis: a nationwide cohort study. *J Neurology Neurosurg Psychiatry* 2021; 92: 556–562.

[140] Earla JR, Hutton GJ, Thornton DJ, et al. Comparative treatment effectiveness of oral fingolimod and conventional injectable disease‐modifying agents in multiple sclerosis. *Pharmacother J Hum Pharmacol Drug Ther* 2021; 41: 440–450.

[141] Ysrraelit MC, Caride A, Sinay V, et al. Real-world effectiveness of natalizumab treatment in patients with relapsing multiple sclerosis in Argentina and Chile. *Arq Neuro-psiquiat* 2021; 79: 407–414.

[142] Papp V, Buron MD, Siersma V, et al. Real-world outcomes for a complete nationwide cohort of more than 3200 teriflunomide-treated multiple sclerosis patients in The Danish Multiple Sclerosis Registry. *Plos One* 2021; 16: e0250820.

[143] Abdel-Mannan OA, Manchoon C, Rossor T, et al. Use of Disease-Modifying Therapies in Pediatric Relapsing-Remitting Multiple Sclerosis in the United Kingdom. *Neurology - Neuroimmunol Neuroinflammation* 2021; 8: e1008.

[144] Huttner A, Lascano AM, Roth S, et al. Rabies vaccination and multiple sclerosis relapse: A retrospective cohort study. *Mult Scler Relat Dis* 2021; 51: 102906.

[145] Reder AT, Arndt N, Roman C, et al. Real-world propensity score comparison of treatment effectiveness of peginterferon beta-1a vs. subcutaneous interferon beta-1a, glatiramer acetate, and teriflunomide in patients with relapsing-remitting multiple sclerosis. *Mult Scler Relat Dis* 2021; 51: 102935.

[146] Häußler V, Ufer F, Pöttgen J, et al. aHSCT is superior to alemtuzumab in maintaining NEDA and improving cognition in multiple sclerosis. *Ann Clin Transl Neur* 2021; 8: 1269–1278.

[147] Capra R, Morra VB, Mirabella M, et al. Natalizumab is associated with early improvement of working ability in relapsing-remitting multiple sclerosis patients: WANT observational study results. *Neurol Sci* 2021; 42: 2837–2845.

[148] Das J, Snowden J, Burman J, et al. Autologous haematopoietic stem cell transplantation as a first-line disease-modifying therapy in patients with ‘aggressive’ multiple sclerosis. *Multiple Scler Houndmills Basingstoke Engl* 2021; 27: 1198–1204.

[149] Bose G, Rush C, Atkins HL, et al. A real-world single-centre analysis of alemtuzumab and cladribine for multiple sclerosis. *Mult Scler Relat Dis* 2021; 52: 102945.

[150] Perumal J, Balabanov R, Su R, et al. Natalizumab in Early Relapsing-Remitting Multiple Sclerosis: A 4-Year, Open-Label Study. *Adv Ther* 2021; 38: 3724–3742.

[151] Kim KH, Kim S-H, Park NY, et al. Real-World Effectiveness of Natalizumab in Korean Patients With Multiple Sclerosis. *Front Neurol* 2021; 12: 714941.

[152] Braune S, Rossnagel F, Dikow H, et al. Impact of drug diversity on treatment effectiveness in relapsing-remitting multiple sclerosis (RRMS) in Germany between 2010 and 2018: real-world data from the German NeuroTransData multiple sclerosis registry. *Bmj Open* 2021; 11: e042480.

[153] Lin T-Y, Vitkova V, Asseyer S, et al. Increased Serum Neurofilament Light and Thin Ganglion Cell–Inner Plexiform Layer Are Additive Risk Factors for Disease Activity in Early Multiple Sclerosis. *Neurology - Neuroimmunol Neuroinflammation* 2021; 8: e1051.

[154] Cohen M, Mondot L, Bucciarelli F, et al. BEST-MS: A prospective head-to-head comparative study of natalizumab and fingolimod in active relapsing MS. *Mult Scler J* 2020; 27: 1556–1563.

[155] Pfeuffer S, Ruck T, Pul R, et al. Impact of previous disease-modifying treatment on effectiveness and safety outcomes, among patients with multiple sclerosis treated with alemtuzumab. *J Neurology Neurosurg Psychiatry* 2021; 92: 1007–1013.

[156] Gisleskog PO, Valenzuela B, Scherz T, et al. An Exposure-Response Analysis of the Clinical Efficacy of Ponesimod in a Randomized Phase II Study in Patients with Multiple Sclerosis. *Clin Pharmacokinet* 2021; 60: 1227–1237.

[157] Rolfes L, Pawlitzki M, Pfeuffer S, et al. Ocrelizumab Extended Interval Dosing in Multiple Sclerosis in Times of COVID-19. *Neurology - Neuroimmunol Neuroinflammation* 2021; 8: e1035.

[158] Efthimios D, Georgios K, Antonia A, et al. Long-Term Effectiveness of Natalizumab in Patients with Relapsing-Remitting Multiple Sclerosis Treated in the Routine Care in Greece: Results from the Multicenter, Observational 5-Year Prospective Study ‘TOPICS Greece.’ *Clin Drug Invest* 2021; 41: 865–874.

[159] Meca-Lallana JE, Oreja-Guevara C, Muñoz D, et al. Four-year safety and effectiveness data from patients with multiple sclerosis treated with fingolimod: The Spanish GILENYA registry. *Plos One* 2021; 16: e0258437.

[160] Guger M, Enzinger C, Leutmezer F, et al. Long-term outcome and predictors of long-term disease activity in natalizumab-treated patients with multiple sclerosis: real life data from the Austrian MS Treatment Registry. *J Neurol* 2021; 268: 4303–4310.

[161] Pérez CA, Lincoln JA. Racial and ethnic disparities in treatment response and tolerability in multiple sclerosis: A comparative study. *Mult Scler Relat Dis* 2021; 56: 103248.

[162] Petracca M, Quarantelli M, Moccia M, et al. ProspeCtive study to evaluate efficacy, safety and tOlerability of dietary supplemeNT of Curcumin (BCM95) in subjects with Active relapsing MultIple Sclerosis treated with subcutaNeous Interferon beta 1a 44 mcg TIW (CONTAIN): A randomized, controlled trial. *Mult Scler Relat Dis* 2021; 56: 103274.

[163] Zmira O, Halpern AI, Abraham L, et al. Efficacy and safety of alemtuzumab treatment in a real-world cohort of patients with multiple sclerosis. *Acta Neurol Belg* 2021; 121: 1513–1518.

[164] Chitnis T, Banwell B, Kappos L, et al. Safety and efficacy of teriflunomide in paediatric multiple sclerosis (TERIKIDS): a multicentre, double-blind, phase 3, randomised, placebo-controlled trial. *Lancet Neurology* 2021; 20: 1001–1011.

[165] AlRuthia Y, Balkhi B, Alkhalifah SA, et al. Real-World Comparative Cost-Effectiveness Analysis of Different Classes of Disease-Modifying Therapies for Relapsing-Remitting Multiple Sclerosis in Saudi Arabia. *Int J Environ Res Pu* 2021; 18: 13261.

[166] Salvetti M, Wray S, Nelles G, et al. Safety and clinical effectiveness of peginterferon beta-1a for relapsing multiple sclerosis in the real-world setting: Interim results from the Plegridy Observational Program. *Mult Scler Relat Dis* 2022; 57: 103350.

[167] Newsome SD, Scott TF, Arnold DL, et al. Early treatment responses to peginterferon beta-1a are associated with longer-term clinical outcomes in patients with relapsing-remitting multiple sclerosis: Subgroup analyses of ADVANCE and ATTAIN. *Mult Scler Relat Dis* 2022; 57: 103367.

[168] Hellwig K, Tokic M, Thiel S, et al. Multiple Sclerosis Disease Activity and Disability Following Discontinuation of Natalizumab for Pregnancy. *Jama Netw Open* 2022; 5: e2144750.

[169] Hänninen K, Viitala M, Atula S, et al. Initial treatment strategy and clinical outcomes in Finnish MS patients: a propensity-matched study. *J Neurol* 2022; 269: 913–922.

[170] Bosco‐Lévy P, Debouverie M, Brochet B, et al. Comparative effectiveness of dimethyl fumarate in multiple sclerosis. *Brit J Clin Pharmaco* 2022; 88: 1268–1278.

[171] Weinstock-Guttman B, Bermel R, Cutter G, et al. Ocrelizumab treatment for relapsing-remitting multiple sclerosis after a suboptimal response to previous disease-modifying therapy: A nonrandomized controlled trial. *Mult Scler J* 2021; 28: 790–800.

[172] Gold R, Arnold DL, Bar-Or A, et al. Long-term safety and efficacy of dimethyl fumarate for up to 13 years in patients with relapsing-remitting multiple sclerosis: Final ENDORSE study results. *Multiple Scler Houndmills Basingstoke Engl* 2022; 28: 801–816.

[173] Almatrafi YM, Babakkor MA, Irfan M, et al. Efficacy and safety of rituximab in patients with multiple sclerosis: An observational study at a tertiary center in Makkah, Saudi Arabia. *Neurosciences* 2022; 27: 65–70.

[174] Lanzillo R, Carotenuto A, Signoriello E, et al. Prognostic Markers of Ocrelizumab Effectiveness in Multiple Sclerosis: A Real World Observational Multicenter Study. *J Clin Medicine* 2022; 11: 2081.

[175] Tichá V, Počíková Z, Vytlačil J, et al. Real-world effectiveness and safety of fingolimod in patients with multiple sclerosis in the Czech Republic: results from core and extension parts of the GOLEMS study up to 48 months. *Bmc Neurol* 2022; 22: 143.

[176] Rauma I, Viitala M, Kuusisto H, et al. Finnish multiple sclerosis patients treated with cladribine tablets: a nationwide registry study. *Mult Scler Relat Dis* 2022; 61: 103755.

[177] Rowles WM, Hsu W-Y, McPolin K, et al. Transitioning From S1P Receptor Modulators to B Cell–Depleting Therapies in Multiple Sclerosis: Clinical, Radiographic, and Laboratory Data. *Neurology - Neuroimmunol Neuroinflammation* 2022; 9: e1183.

[178] Mariottini A, Bulgarini G, Forci B, et al. Autologous haematopoietic stem cell transplantation versus low‐dose immunosuppression in secondary–progressive multiple sclerosis. *Eur J Neurol* 2022; 29: 1708–1718.

[179] Papeix C, Castelnovo G, Leray E, et al. Long-Term Effectiveness, Safety and Tolerability of Fingolimod in Patients with Multiple Sclerosis in Real-World Treatment Settings in France: The VIRGILE Study. *Neurology Ther* 2022; 11: 633–658.

[180] Nygaard GO, Torgauten H, Skattebøl L, et al. Risk of fingolimod rebound after switching to cladribine or rituximab in multiple sclerosis. *Mult Scler Relat Dis* 2022; 62: 103812.

[181] Brune S, Høgestøl EA, Benavent SA de R, et al. Serum neurofilament light chain concentration predicts disease worsening in multiple sclerosis. *Multiple Scler Houndmills Basingstoke Engl* 2022; 13524585221097296.

[182] Bsteh G, Assar H, Gradl C, et al. Long‐term outcome after COVID‐19 infection in multiple sclerosis: A nation‐wide multicenter matched‐control study. *Eur J Neurol* 2022; 29: 10.1111/ene.15477.

[183] Foley JF, Defer G, Ryerson LZ, et al. Comparison of switching to 6-week dosing of natalizumab versus continuing with 4-week dosing in patients with relapsing-remitting multiple sclerosis (NOVA): a randomised, controlled, open-label, phase 3b trial. *Lancet Neurology* 2022; 21: 608–619.

[184] Zhong M, Walt A van der, Monif M, et al. Prediction of relapse activity when switching to cladribine for multiple sclerosis. *Mult Scler J* 2022; 135245852211116.

[185] Svenningsson A, Frisell T, Burman J, et al. Safety and efficacy of rituximab versus dimethyl fumarate in patients with relapsing-remitting multiple sclerosis or clinically isolated syndrome in Sweden: a rater-blinded, phase 3, randomised controlled trial. *Lancet Neurology* 2022; 21: 693–703.

[186] Simoneau G, Jiang X, Rollot F, et al. Overall and patient-level comparative effectiveness of dimethyl fumarate and fingolimod: A precision medicine application to the Observatoire Français de la Sclérose en Plaques registry. *Multiple Scler J - Exp Transl Clin* 2022; 8: 20552173221116590.

[187] Roos I, Malpas C, Leray E, et al. Disease Reactivation After Cessation of Disease-Modifying Therapy in Patients With Relapsing-Remitting Multiple Sclerosis. *Neurology* 2022; 99: e1926–e1944.

[188] Zanghì A, Avolio C, Signoriello E, et al. Is It Time for Ocrelizumab Extended Interval Dosing in Relapsing Remitting MS? Evidence from An Italian Multicenter Experience During the COVID-19 Pandemic. *Neurotherapeutics* 2022; 19: 1535–1545.

[189] Zhang T, Kingwell E, Zhu F, et al. Effect of adherence to the first-generation injectable immunomodulatory drugs on disability accumulation in multiple sclerosis: a longitudinal cohort study. *Bmj Open* 2017; 7: e018612.

[190] Dahbour S, Jamali F, Alhattab D, et al. Mesenchymal stem cells and conditioned media in the treatment of multiple sclerosis patients: Clinical, ophthalmological and radiological assessments of safety and efficacy. *Cns Neurosci Ther* 2017; 23: 866–874.

[191] Planche V, Moisset X, Morello R, et al. Improvement of quality of life and its relationship with neuropsychiatric outcomes in patients with multiple sclerosis starting treatment with natalizumab: A 3-year follow-up multicentric study. *J Neurol Sci* 2017; 382: 148–154.

[192] Zandi-Esfahan S, Fazeli M, Shaygannejad V, et al. Evaluating the effect of adding Fish oil to Fingolimod on TNF-α, IL1β, IL6, and IFN-γ in patients with relapsing-remitting multiple sclerosis: A double-blind randomized placebo-controlled trial. *Clin Neurol Neurosur* 2017; 163: 173–178.

[193] Rilo O, Peña J, Ojeda N, et al. Integrative group-based cognitive rehabilitation efficacy in multiple sclerosis: a randomized clinical trial. *Disabil Rehabil* 2018; 40: 208–216.

[194] Wang L, Qi C-H, Zhong R, et al. Efficacy of alemtuzumab and natalizumab in the treatment of different stages of multiple sclerosis patients. *Medicine* 2018; 97: e9908.

[195] Kappos L, Bar-Or A, Cree BAC, et al. Siponimod versus placebo in secondary progressive multiple sclerosis (EXPAND): a double-blind, randomised, phase 3 study. *Lancet* 2018; 391: 1263–1273.

[196] Alsaeed MO, Harding KE, Williams OH, et al. Multiple sclerosis: long‐term outcomes in ethnic minorities. Analysis of a UK population‐based registry. *Eur J Neurol* 2018; 25: 701–704.

[197] Stork L, Ellenberger D, Beißbarth T, et al. Differences in the Reponses to Apheresis Therapy of Patients With 3 Histopathologically Classified Immunopathological Patterns of Multiple Sclerosis. *Jama Neurol* 2018; 75: 428.

[198] Kapoor R, Ho P-R, Campbell N, et al. Effect of natalizumab on disease progression in secondary progressive multiple sclerosis (ASCEND): a phase 3, randomised, double-blind, placebo-controlled trial with an open-label extension. *Lancet Neurology* 2018; 17: 405–415.

[199] Trojano M, Butzkueven H, Kappos L, et al. Natalizumab treatment shows low cumulative probabilities of confirmed disability worsening to EDSS milestones in the long-term setting. *Mult Scler Relat Dis* 2018; 24: 11–19.

[200] Bhan A, Jacobsen C, Myhr K-M, et al. Neurofilaments and 10-year follow-up in multiple sclerosis. *Multiple Scler Houndmills Basingstoke Engl* 2018; 24: 1301–1307.

[201] Healy BC, Glanz BI, Zurawski JD, et al. Long-term follow-up for multiple sclerosis patients initially treated with interferon-beta and glatiramer acetate. *J Neurol Sci* 2018; 394: 127–131.

[202] Cohan S, Kappos L, Giovannoni G, et al. Efficacy of daclizumab beta versus intramuscular interferon beta-1a on disability progression across patient demographic and disease activity subgroups in DECIDE. *Multiple Scler Houndmills Basingstoke Engl* 2018; 24: 1883–1891.

[203] Palace J, Duddy M, Lawton M, et al. Assessing the long-term effectiveness of interferon-beta and glatiramer acetate in multiple sclerosis: final 10-year results from the UK multiple sclerosis risk-sharing scheme. *J Neurology Neurosurg Psychiatry* 2019; 90: 251.

[204] Hervás‐García JV, Ramió‐Torrentà L, Brieva‐Ruiz L, et al. Comparison of two high doses of oral methylprednisolone for multiple sclerosis relapses: a pilot, multicentre, randomized, double‐blind, non‐inferiority trial. *Eur J Neurol* 2019; 26: 525–532.

[205] Harding K, Williams O, Willis M, et al. Clinical Outcomes of Escalation vs Early Intensive Disease-Modifying Therapy in Patients With Multiple Sclerosis. *Jama Neurol* 2019; 76: 536–541.

[206] Crielaard L, Kavaliunas A, Ramanujam R, et al. Factors associated with and long-term outcome of benign multiple sclerosis: a nationwide cohort study. *J Neurology Neurosurg Psychiatry* 2019; 90: 761.

[207] Brownlee WJ, Solanky B, Prados F, et al. Cortical grey matter sodium accumulation is associated with disability and secondary progressive disease course in relapse-onset multiple sclerosis. *J Neurology Neurosurg Psychiatry* 2019; 90: 755.

[208] Newsome SD, Geldern G von, Shou H, et al. Longitudinal assessment of hand function in individuals with multiple sclerosis. *Mult Scler Relat Dis* 2019; 32: 107–113.

[209] Drulovic J, Ivanovic J, Mesaros S, et al. Long-term disability outcomes in relapsing-remitting multiple sclerosis: a 10-year follow-up study. *Neurol Sci* 2019; 40: 1–10.

[210] Dekker I, Leurs CE, Hagens MHJ, et al. Long-term disease activity and disability progression in relapsing-remitting multiple sclerosis patients on natalizumab. *Mult Scler Relat Dis* 2019; 33: 82–87.

[211] Cadavid D, Mellion M, Hupperts R, et al. Safety and efficacy of opicinumab in patients with relapsing multiple sclerosis (SYNERGY): a randomised, placebo-controlled, phase 2 trial. *Lancet Neurology* 2019; 18: 845–856.

[212] Dorst J, Fangerau T, Taranu D, et al. Safety and efficacy of immunoadsorption versus plasma exchange in steroid-refractory relapse of multiple sclerosis and clinically isolated syndrome: A randomised, parallel-group, controlled trial. *Eclinicalmedicine* 2019; 16: 98–106.

[213] Goldman MD, LaRocca NG, Rudick RA, et al. Evaluation of multiple sclerosis disability outcome measures using pooled clinical trial data. *Neurology* 2019; 93: 10.1212/WNL.0000000000008519.

[214] Järvinen E, Murtonen A, Tervomaa M, et al. Interferon β-1a subcutaneously 3 times/week clinical outcome in relapsing multiple sclerosis in Finland. *Neurology Int* 2019; 11: 8177.

[215] Spelman T, Freilich J, Anell B, et al. Patients With High-disease-activity Relapsing-Remitting Multiple Sclerosis in Real-world Clinical Practice: A Population-based Study in Sweden. *Clin Ther* 2020; 42: 240–250.

[216] Muthuraman M, Fleischer V, Kroth J, et al. Covarying patterns of white matter lesions and cortical atrophy predict progression in early MS. *Neurology - Neuroimmunol Neuroinflammation* 2020; 7: e681.

[217] He A, Merkel B, Brown JWL, et al. Timing of high-efficacy therapy for multiple sclerosis: a retrospective observational cohort study. *Lancet Neurology* 2020; 19: 307–316.

[218] Inojosa H, Schriefer D, Ziemssen T. Clinical outcome measures in multiple sclerosis: A review. *Autoimmun Rev* 2020; 19: 102512.

[219] Daniels K, Nat PB van der, Frequin STFM, et al. Real-World Results of Ocrelizumab Treatment for Primary Progressive Multiple Sclerosis. *Multiple Scler Int* 2020; 2020: 5463451.

[220] Prosperini L, Mancinelli CR, Solaro CM, et al. Induction Versus Escalation in Multiple Sclerosis: A 10-Year Real World Study. *Neurotherapeutics* 2020; 17: 994–1004.

[221] Guger M, Enzinger C, Leutmezer F, et al. Oral therapies for treatment of relapsing–remitting multiple sclerosis in Austria: a 2-year comparison using an inverse probability weighting method. *J Neurol* 2020; 267: 2090–2100.

[222] Brochet B, Deloire M, Germain C, et al. Double‐blind, randomized controlled trial of therapeutic plasma exchanges vs sham exchanges in moderate‐to‐severe relapses of multiple sclerosis. *J Clin Apheresis* 2020; 35: 281–289.

[223] Reyes S, Smets I, Holden D, et al. CSF neurofilament light chain testing as an aid to determine treatment strategies in MS. *Neurology - Neuroimmunol Neuroinflammation* 2020; 7: e880.

[224] Giovannoni G, Knappertz V, Steinerman JR, et al. A randomized, placebo-controlled, phase 2 trial of laquinimod in primary progressive multiple sclerosis. *Neurology* 2020; 95: e1027–e1040.

[225] Koch MW, Mostert J, Uitdehaag B, et al. Clinical outcome measures in SPMS trials: An analysis of the IMPACT and ASCEND original trial data sets. *Mult Scler J* 2019; 26: 1540–1549.

[226] Kaufmann M, Vaney C, Barin L, et al. Long-term worsening of different body functions in persons with progressive multiple sclerosis. *Multiple Scler J Exp Transl Clin* 2020; 6: 2055217320964514.

[227] Lizak N, Malpas CB, Sharmin S, et al. Association of Sustained Immunotherapy With Disability Outcomes in Patients With Active Secondary Progressive Multiple Sclerosis. *Jama Neurol*; 77. Epub ahead of print 2020. DOI: 10.1001/jamaneurol.2020.2453.

[228] Tourbah A, Papeix C, Tourniaire P, et al. Reasons for switching to fingolimod in patients relapsing-remitting multiple sclerosis in France: the ESGILE study. *Mult Scler Relat Dis* 2020; 46: 102433.

[229] Silva AM da, Torres C, Ferreira I, et al. Prognostic value of odor identification impairment in multiple sclerosis: 10-Years follow-up. *Mult Scler Relat Dis* 2020; 46: 102486.

[230] Couloume L, Barbin L, Leray E, et al. High-dose biotin in progressive multiple sclerosis: A prospective study of 178 patients in routine clinical practice. *Mult Scler J* 2019; 26: 1898–1906.

[231] Cree BAC, Cutter G, Wolinsky JS, et al. Safety and efficacy of MD1003 (high-dose biotin) in patients with progressive multiple sclerosis (SPI2): a randomised, double-blind, placebo-controlled, phase 3 trial. *Lancet Neurology* 2020; 19: 988–997.

[232] Cortese R, Tur C, Prados F, et al. Ongoing microstructural changes in the cervical cord underpin disability progression in early primary progressive multiple sclerosis. *Mult Scler J* 2020; 27: 28–38.

[233] Mariottini A, Filippini S, Innocenti C, et al. Impact of autologous haematopoietic stem cell transplantation on disability and brain atrophy in secondary progressive multiple sclerosis. *Multiple Scler Houndmills Basingstoke Engl* 2020; 27: 61–70.

[234] Koch MW, Mostert J, Repovic P, et al. Reliability of Outcome Measures in Clinical Trials in Secondary Progressive Multiple Sclerosis. *Neurology* 2020; 96: e111–e120.

[235] Lejeune F, Chatton A, Laplaud D-A, et al. SMILE: a predictive model for Scoring the severity of relapses in MultIple scLErosis. *J Neurol* 2021; 268: 669–679.

[236] Bellmann-Strobl J, Paul F, Wuerfel J, et al. Epigallocatechin Gallate in Relapsing-Remitting Multiple Sclerosis: A Randomized, Placebo-Controlled Trial. *Neurology - Neuroimmunol Neuroinflammation* 2021; 8: e981.

[237] Sormani MP, Freedman MS, Aldridge J, et al. MAGNIMS score predicts long-term clinical disease activity-free status and confirmed disability progression in patients treated with subcutaneous interferon beta-1a. *Mult Scler Relat Dis* 2021; 49: 102790.

[238] Healy BC, Glanz BI, Swallow E, et al. Confirmed disability progression provides limited predictive information regarding future disease progression in multiple sclerosis. *Multiple Scler J Exp Transl Clin* 2021; 7: 2055217321999070.

[239] Koch MW, Mostert J, Repovic P, et al. Is the Symbol Digit Modalities Test a useful outcome in secondary progressive multiple sclerosis? *Eur J Neurol* 2021; 28: 2115–2120.

[240] Keller J, Zackowski K, Kim S, et al. Exercise leads to metabolic changes associated with improved strength and fatigue in people with MS. *Ann Clin Transl Neur* 2021; 8: 1308–1317.

[241] Haider L, Prados F, Chung K, et al. Cortical involvement determines impairment 30 years after a clinically isolated syndrome. *Brain* 2021; 144: awab033-.

[242] Ow N, Kuspinar A, Mayo NE, et al. Longitudinal analysis of disability outcomes among young people with MS. *Mult Scler Relat Dis* 2021; 52: 102966.

[243] Krajnc N, Berger T, Bsteh G. Measuring Treatment Response in Progressive Multiple Sclerosis—Considerations for Adapting to an Era of Multiple Treatment Options. *Biomol* 2021; 11: 1342.

[244] Koch MW, Mostert JP, Uitdehaag B, et al. A comparison of clinical outcomes in PPMS in the INFORMS original trial data set. *Multiple Scler Houndmills Basingstoke Engl* 2021; 27: 1864–1874.

[245] Marrodan M, Crema S, Rubstein A, et al. Therapeutic plasma exchange in MS refractory relapses: Long-term outcome. *Mult Scler Relat Dis* 2021; 55: 103168.

[246] Spelman T, Magyari M, Piehl F, et al. Treatment Escalation vs Immediate Initiation of Highly Effective Treatment for Patients With Relapsing-Remitting Multiple Sclerosis. *Jama Neurol* 2021; 78: 1197–1204.

[247] Koch MW, Mostert JP, Wolinsky JS, et al. Comparison of the EDSS, Timed 25-Foot Walk, and the 9-Hole Peg Test as Clinical Trial Outcomes in Relapsing-Remitting Multiple Sclerosis. *Neurology* 2021; 97: e1560–e1570.

[248] Cree BA, Cohen JA, Reder AT, et al. Disability improvement as a clinically relevant outcome in clinical trials of relapsing forms of multiple sclerosis. *Mult Scler J* 2021; 27: 2219–2231.

[249] Lagrèze WA, Küchlin S, Ihorst G, et al. Safety and efficacy of erythropoietin for the treatment of patients with optic neuritis (TONE): a randomised, double-blind, multicentre, placebo-controlled study. *Lancet Neurology* 2021; 20: 991–1000.

[250] Kalinowski A, Cutter G, Bozinov N, et al. The timed 25-foot walk in a large cohort of multiple sclerosis patients. *Multiple Scler Houndmills Basingstoke Engl* 2022; 28: 289–299.

[251] Petracca M, Cutter G, Cocozza S, et al. Cerebellar pathology and disability worsening in relapsing‐remitting multiple sclerosis: A retrospective analysis from the CombiRx trial. *Eur J Neurol* 2022; 29: 515–521.

[252] Vermersch P, Brieva-Ruiz L, Fox RJ, et al. Efficacy and Safety of Masitinib in Progressive Forms of Multiple Sclerosis: A Randomized, Phase 3, Clinical Trial. *Neurology - Neuroimmunol Neuroinflammation* 2022; 9: e1148.

[253] Chen B, Ji S-Q, Shen F, et al. Contribution of relapse-associated worsening to overall disability accrual in patients with relapsing-onset multiple sclerosis: A mediation analysis. *Mult Scler Relat Dis* 2022; 59: 103555.

[254] Koch MW, Mostert J, Repovic P, et al. MRI brain volume loss, lesion burden, and clinical outcome in secondary progressive multiple sclerosis. *Multiple Scler Houndmills Basingstoke Engl* 2022; 28: 561–572.

[255] Mueller C, Baird JF, Motl RW. Whole-Brain Metabolic Abnormalities Are Associated With Mobility in Older Adults With Multiple Sclerosis. *Neurorehab Neural Re* 2022; 36: 286–297.

[256] Koch MW, Mostert J, Repovic P, et al. The timed 25-foot walk is a more sensitive outcome measure than the EDSS for PPMS trials: an analysis of the PROMISE clinical trial dataset. *J Neurol* 2022; 269: 5319–5327.

[257] Zanghì A, D’Amico E, Patti F, et al. Stopping Interferon Beta 1b Does Not Influence the Risk of Disability Accrual in Non-Active SPMS: Results from an Italian Real-World Study. *Int J Environ Res Pu* 2022; 19: 6069.

[258] Leppert D, Kropshofer H, Häring DA, et al. Blood Neurofilament Light in Progressive Multiple Sclerosis: Post Hoc Analysis of 2 Randomized Controlled Trials. *Neurology* 2021; 98: e2120–e2131.

[259] Testud B, Delacour C, Ahmadi AAE, et al. Brain grey matter perfusion in primary progressive multiple sclerosis: Mild decrease over years and regional associations with cognition and hand function. *Eur J Neurol* 2022; 29: 1741–1752.

[260] Brenton JN, Lehner-Gulotta D, Woolbright E, et al. Phase II study of ketogenic diets in relapsing multiple sclerosis: safety, tolerability and potential clinical benefits. *J Neurology Neurosurg Psychiatry* 2022; 93: 637–644.

[261] Lie IA, Wesnes K, Kvistad SS, et al. The Effect of Smoking on Long-term Gray Matter Atrophy and Clinical Disability in Patients with Relapsing-Remitting Multiple Sclerosis. *Neurology - Neuroimmunol Neuroinflammation* 2022; 9: e200008.

[262] Koch MW, Mostert J, Repovic P, et al. Impact of clinical outcomes and imaging measures on health-related quality of life in secondary progressive MS. *Mult Scler J* 2021; 28: 1286–1298.

[263] Alcalá C, Quintanilla-Bordás C, Gascón F, et al. Effectiveness of rituximab vs. ocrelizumab for the treatment of primary progressive multiple sclerosis: a real-world observational study. *J Neurol* 2022; 269: 3676–3681.

[264] Portaccio E, Fonderico M, Iaffaldano P, et al. Disease-Modifying Treatments and Time to Loss of Ambulatory Function in Patients With Primary Progressive Multiple Sclerosis. *Jama Neurol* 2022; 79: 869–878.

[265] Bose G, Healy BC, Barro C, et al. Younger age at multiple sclerosis onset is associated with worse outcomes at age 50. *J Neurology Neurosurg Psychiatry* 2022; 93: 1112–1119.

[266] Pauwels A, Schependom JV, Devolder L, et al. Plasma glial fibrillary acidic protein and neurofilament light chain in relation to disability worsening in multiple sclerosis. *Mult Scler J* 2022; 28: 1685–1696.

[267] Schwid SR, Goodman AD, McDermott MP, et al. Quantitative functional measures in MS: what is a reliable change? *Neurology* 2002; 58: 1294–6.

[268] Learmonth YC, Dlugonski DD, Pilutti LA, et al. The reliability, precision and clinically meaningful change of walking assessments in multiple sclerosis. *Mult Scler J* 2013; 19: 1784–1791.

[269] Hobart J, Blight AR, Goodman A, et al. Timed 25-Foot Walk: Direct evidence that improving 20% or greater is clinically meaningful in MS. *Neurology* 2013; 80: 1509–1517.

[270] Tajali S, Shaterzadeh-Yazdi M-J, Negahban H, et al. Predicting falls among patients with multiple sclerosis: Comparison of patient-reported outcomes and performance-based measures of lower extremity functions. *Mult Scler Relat Dis* 2017; 17: 69–74.

[271] Alvarez-Payero M, Valeiras-Muñoz C, Lion-Vázquez S, et al. Experience with fampridine in clinical practice: analysis of a possible marker of clinical response. *Int J Neurosci* 2017; 127: 915–922.

[272] Almuklass AM, Davis L, Hamilton LD, et al. Pulse Width Does Not Influence the Gains Achieved With Neuromuscular Electrical Stimulation in People With Multiple Sclerosis: Double-Blind, Randomized Trial. *Neurorehab Neural Re* 2018; 32: 84–93.

[273] Maniscalco GT, Aponte R, Bruzzese D, et al. THC/CBD oromucosal spray in patients with multiple sclerosis overactive bladder: a pilot prospective study. *Neurol Sci* 2018; 39: 97–102.

[274] Sola-Valls N, Blanco Y, Sepúlveda M, et al. Combined walking outcome measures identify clinically meaningful response to prolonged-release fampridine. *Ther Adv Neurol Diso* 2018; 11: 1756286418780007.

[275] Klineova S, Farber R, Friedman J, et al. Objective and subjective measures of dalfampridine efficacy in clinical practice. *Multiple Scler J Exp Transl Clin* 2018; 4: 2055217318786742.

[276] Rodriguez-Leal FA, Haase R, Thomas K, et al. Fampridine response in MS patients with gait impairment in a real-world setting: Need for new response criteria? *Mult Scler J* 2017; 24: 1337–1346.

[277] Leone C, Kalron A, Smedal T, et al. Effects of Rehabilitation on Gait Pattern at Usual and Fast Speeds Depend on Walking Impairment Level in Multiple Sclerosis. *Int J Ms Care* 2018; 20: 199–209.

[278] Edwards T, Motl RW, Sebastião E, et al. Pilot randomized controlled trial of functional electrical stimulation cycling exercise in people with multiple sclerosis with mobility disability. *Mult Scler Relat Dis* 2018; 26: 103–111.

[279] Fjeldstad-Pardo C, Thiessen A, Pardo G. Telerehabilitation in Multiple Sclerosis: Results of a Randomized Feasibility and Efficacy Pilot Study. *Int J Telerehabilitation* 2018; 10: 55–64.

[280] Baquet L, Hasselmann H, Patra S, et al. Short-term interval aerobic exercise training does not improve memory functioning in relapsing-remitting multiple sclerosis—a randomized controlled trial. *Peerj* 2018; 6: e6037.

[281] Bakirtzis C, Konstantinopoulou E, Langdon DW, et al. Long-term effects of prolonged-release fampridine in cognitive function, fatigue, mood and quality of life of MS patients: The IGNITE study. *J Neurol Sci* 2018; 395: 106–112.

[282] Decavel P, Moulin T, Sagawa Y. Gait tests in multiple sclerosis: Reliability and cut-off values. *Gait Posture* 2019; 67: 37–42.

[283] Feys P, Moumdjian L, Halewyck FV, et al. Effects of an individual 12-week community-located “start-to-run” program on physical capacity, walking, fatigue, cognitive function, brain volumes, and structures in persons with multiple sclerosis. *Mult Scler J* 2017; 25: 92–103.

[284] Bromley L, Horvath PJ, Bennett SE, et al. Impact of Nutritional Intake on Function in People with Mild-to-Moderate Multiple Sclerosis. *Int J Ms Care* 2019; 21: 1–9.

[285] Paul L, Renfrew L, Freeman J, et al. Web-based physiotherapy for people affected by multiple sclerosis: a single blind, randomized controlled feasibility study. *Clin Rehabil* 2018; 33: 473–484.

[286] Cohen JA, Hunter SF, Brown TR, et al. Safety and efficacy of ADS-5102 (amantadine) extended release capsules to improve walking in multiple sclerosis: A randomized, placebo-controlled, phase 2 trial. *Mult Scler J* 2018; 25: 601–609.

[287] Oveisgharan S, Karimi Z, Abdi S, et al. The use of brain stimulation in the rehabilitation of walking disability in patients with multiple sclerosis: A randomized double-blind clinical trial study. *Iranian J Neurology* 2019; 18: 57–63.

[288] Renfrew L (Miller), Paul L, McFadyen A, et al. The clinical- and cost-effectiveness of functional electrical stimulation and ankle-foot orthoses for foot drop in Multiple Sclerosis: a multicentre randomized trial. *Clin Rehabil* 2019; 33: 1150–1162.

[289] Giglio LD, Luca FD, Gurreri F, et al. Effect of dalfampridine on information processing speed impairment in multiple sclerosis. *Neurology* 2019; 93: e733–e746.

[290] Cambron M, Mostert J, D’Hooghe M, et al. Fluoxetine in progressive multiple sclerosis: The FLUOX-PMS trial. *Mult Scler J* 2019; 25: 1728–1735.

[291] Satchidanand N, Drake A, Smerbeck A, et al. Dalfampridine benefits ambulation but not cognition in multiple sclerosis. *Mult Scler J* 2018; 26: 91–98.

[292] Almuklass AM, Capobianco RA, Feeney DF, et al. Sensory nerve stimulation causes an immediate improvement in motor function of persons with multiple sclerosis: A pilot study. *Mult Scler Relat Dis* 2020; 38: 101508.

[293] Munster CEP van, Kaya L, Lam KH, et al. Responder rates to fampridine differ between clinical subgroups of MS patients and patient reported outcome influences treatment decision making. *Mult Scler Relat Dis* 2020; 38: 101489.

[294] Straudi S, Manfredini F, Lamberti N, et al. Robot-assisted gait training is not superior to intensive overground walking in multiple sclerosis with severe disability (the RAGTIME study): A randomized controlled trial. *Mult Scler J* 2019; 26: 716–724.

[295] Ozdogar AT, Ertekin O, Kahraman T, et al. Effect of video-based exergaming on arm and cognitive function in persons with multiple sclerosis: A randomized controlled trial. *Mult Scler Relat Dis* 2020; 40: 101966.

[296] Kahraman T, Savci S, Ozdogar AT, et al. Physical, cognitive and psychosocial effects of telerehabilitation-based motor imagery training in people with multiple sclerosis: A randomized controlled pilot trial. *J Telemed Telecare* 2018; 26: 251–260.

[297] Geel FV, Geurts E, Abasıyanık Z, et al. Feasibility study of a 10-week community-based program using the WalkWithMe application on physical activity, walking, fatigue and cognition in persons with Multiple Sclerosis. *Mult Scler Relat Dis* 2020; 42: 102067.

[298] Ziliotto N, Lamberti N, Manfredini F, et al. Functional recovery in multiple sclerosis patients undergoing rehabilitation programs is associated with plasma levels of hemostasis inhibitors. *Mult Scler Relat Dis* 2020; 44: 102319.

[299] Donkers SJ, Nickel D, Paul L, et al. Adherence to Physiotherapy-Guided Web-Based Exercise for Persons Living with Moderate-to-Severe Multiple Sclerosis: A Randomized-Controlled Pilot Study. *Int J Ms Care* 2020; 22: 208–214.

[300] Weller D, Lörincz L, Sutter T, et al. Fampridine-induced changes in walking kinetics are associated with clinical improvements in patients with multiple sclerosis. *J Neurol Sci* 2020; 416: 116978.

[301] Chanpimol S, Benson K, Maloni H, et al. Acceptability and outcomes of an individualized exergaming telePT program for veterans with multiple sclerosis: a pilot study. *Archives Physiotherapy* 2020; 10: 18.

[302] Lamberti N, Straudi S, Donadi M, et al. Effectiveness of blood flow‐restricted slow walking on mobility in severe multiple sclerosis: A pilot randomized trial. *Scand J Med Sci Spor* 2020; 30: 1999–2009.

[303] Kosa P, Wu T, Phillips J, et al. Idebenone does not inhibit disability progression in primary progressive MS. *Mult Scler Relat Dis* 2020; 45: 102434.

[304] Hernandez L, O’Donnell M, Postma M, et al. Predictors of Health Utility in Relapsing–Remitting and Secondary-Progressive Multiple Sclerosis: Implications for Future Economic Models of Disease-Modifying Therapies. *Pharmacoeconomics* 2021; 39: 243–256.

[305] Drużbicki M, Guzik A, Przysada G, et al. Effects of Robotic Exoskeleton-Aided Gait Training in the Strength, Body Balance, and Walking Speed in Individuals With Multiple Sclerosis: A Single-Group Preliminary Study. *Arch Phys Med Rehab* 2021; 102: 175–184.

[306] Conway DS, Thompson NR, Meng X, et al. Patient reported outcomes and performance metrics at diagnosis of secondary progressive multiple sclerosis. *Mult Scler J* 2020; 27: 742–754.

[307] Valet M, Sankari SE, Pesch VV, et al. Effects of prolonged-release fampridine on multiple sclerosis-related gait impairments. A crossover, double-blinded, placebo-controlled study. *Clin Biomech* 2021; 86: 105382.

[308] Gulde P, Hermsdörfer J, Rieckmann P. Speed but Not Smoothness of Gait Reacts to Rehabilitation in Multiple Sclerosis. *Multiple Scler Int* 2021; 2021: 5589562.

[309] Sconza C, Negrini F, Matteo BD, et al. Robot-Assisted Gait Training in Patients with Multiple Sclerosis: A Randomized Controlled Crossover Trial. *Medicina* 2021; 57: 713.

[310] Abasıyanık Z, Yiğit P, Özdoğar AT, et al. A comparative study of the effects of yoga and clinical Pilates training on walking, cognition, respiratory functions, and quality of life in persons with multiple sclerosis: A quasi-experimental study. *Explor* 2021; 17: 424–429.

[311] Koch MW, Kaur S, Sage K, et al. Hydroxychloroquine for Primary Progressive Multiple Sclerosis. *Ann Neurol* 2021; 90: 940–948.

[312] Cohen JA, Cameron MH, Goldman MD, et al. A Phase 3, double-blind, placebo-controlled efficacy and safety study of ADS-5102 (Amantadine) extended-release capsules in people with multiple sclerosis and walking impairment. *Multiple Scler Houndmills Basingstoke Engl* 2022; 28: 817–830.

[313] Straudi S, Marco GD, Martinuzzi C, et al. Combining a supervised and home-based task-oriented circuit training improves walking endurance in patients with multiple sclerosis. The MS_TOCT randomized-controlled trial. *Mult Scler Relat Dis* 2022; 60: 103721.

[314] Ozdogar AT, Baba C, Kahraman T, et al. Effects and safety of exergaming in persons with multiple sclerosis during corticosteroid treatment: a pilot study. *Mult Scler Relat Dis* 2022; 63: 103823.

[315] Skov CD, Sørensen CB, Thorning M, et al. Evaluation of functional outcome measures after fampridine treatment in patients with multiple sclerosis - An interventional follow-up study. *Mult Scler Relat Dis* 2022; 66: 104034.

[316] Hvid LG, Stenager E, Dalgas U. Objectively assessed physiological, physical, and cognitive function along with patient-reported outcomes during the first 2 years of Alemtuzumab treatment in multiple sclerosis: a prospective observational study. *J Neurol* 2022; 269: 4895–4908.

[317] Riepl E, Pfeuffer S, Ruck T, et al. Alemtuzumab Improves Cognitive Processing Speed in Active Multiple Sclerosis—A Longitudinal Observational Study. *Front Neurol* 2018; 8: 730.

[318] Stuifbergen AK, Becker H, Perez F, et al. Computer-assisted cognitive rehabilitation in persons with multiple sclerosis: Results of a multi-site randomized controlled trial with six month follow-up. *Disabil Health J* 2018; 11: 427–434.

[319] Eijlers AJC, Geest Q van, Dekker I, et al. Predicting cognitive decline in multiple sclerosis: a 5-year follow-up study. *Brain* 2018; 141: 2605–2618.

[320] Pilutti LA, Edwards T, Motl RW, et al. Functional Electrical Stimulation Cycling Exercise in People with Multiple Sclerosis: Secondary Effects on Cognition, Symptoms, and Quality of Life. *Int J Ms Care* 2019; 21: 258–264.

[321] Johnen A, Elpers C, Riepl E, et al. Early effective treatment may protect from cognitive decline in paediatric multiple sclerosis. *Eur J Paediatr Neuro* 2019; 23: 783–791.

[322] Vilou I, Bakirtzis C, Artemiadis A, et al. Computerized cognitive rehabilitation for treatment of cognitive impairment in multiple sclerosis: an explorative study. *J Integr Neurosci* 2020; 19: 341.

[323] Macaron G, Baldassari LE, Nakamura K, et al. Cognitive processing speed in multiple sclerosis clinical practice: association with patient‐reported outcomes, employment and magnetic resonance imaging metrics. *Eur J Neurol* 2020; 27: 1238–1249.

[324] Jacobsen C, Zivadinov R, Myhr K-M, et al. Brain atrophy and clinical characteristics predicting SDMT performance in multiple sclerosis: A 10-year follow-up study. *Multiple Scler J - Exp Transl Clin* 2021; 7: 2055217321992394.

[325] Leonardi S, Maggio MG, Russo M, et al. Cognitive recovery in people with relapsing/remitting multiple sclerosis: A randomized clinical trial on virtual reality-based neurorehabilitation. *Clin Neurol Neurosur* 2021; 208: 106828.

[326] Leach JM, Cutter G, Golan D, et al. Measuring cognitive function by the SDMT across functional domains: Useful but not sufficient. *Mult Scler Relat Dis* 2022; 60: 103704.

[327] Magnin E, Sagawa Y, Moulin T, et al. What Are the Minimal Detectable Changes in SDMT and Verbal Fluency Tests for Assessing Changes in Cognitive Performance in Persons with Multiple Sclerosis and Non-Multiple Sclerosis Controls? *Eur Neurol* 2020; 83: 263–270.

[328] Morrow SA, Rosehart H, Sener A, et al. Anti-cholinergic medications for bladder dysfunction worsen cognition in persons with multiple sclerosis. *J Neurol Sci* 2018; 385: 39–44.

[329] Bove R, Rowles W, Zhao C, et al. A novel in-home digital treatment to improve processing speed in people with multiple sclerosis: A pilot study. *Mult Scler J* 2020; 27: 778–789.

[330] Strober LB, Bruce JM, Arnett PA, et al. A much needed metric: Defining reliable and statistically meaningful change of the oral version Symbol Digit Modalities Test (SDMT). *Mult Scler Relat Dis* 2022; 57: 103405.

[331] Chiaravalloti ND, Costa SL, Moore NB, et al. The efficacy of speed of processing training for improving processing speed in individuals with multiple sclerosis: a randomized clinical trial. *J Neurol* 2022; 269: 3614–3624.

[332] Reilly S, Hynes SM. A Cognitive Occupation-Based Programme for People with Multiple Sclerosis: A Study to Test Feasibility and Clinical Outcomes. *Occup Ther Int* 2018; 2018: 1614901.

[333] Cree BAC, Arnold DL, Cascione M, et al. Phase IV study of retention on fingolimod versus injectable multiple sclerosis therapies: a randomized clinical trial. *Ther Adv Neurol Diso* 2018; 11: 1756286418774338.

[334] Messinis L, Kosmidis MH, Nasios G, et al. Do Secondary Progressive Multiple Sclerosis patients benefit from Computer- based cognitive neurorehabilitation? A randomized sham controlled trial. *Mult Scler Relat Dis* 2020; 39: 101932.

[335] Ruet A, Deloire MS, Charré-Morin J, et al. A new computerised cognitive test for the detection of information processing speed impairment in multiple sclerosis. *Mult Scler J* 2013; 19: 1665–1672.

[336] Hervault M, Balto JM, Hubbard EA, et al. Reliability, precision, and clinically important change of the Nine-Hole Peg Test in individuals with multiple sclerosis. *Int J Rehabil Res* 2017; 40: 91–93.

[337] Jonsdottir J, Bertoni R, Lawo M, et al. Serious games for arm rehabilitation of persons with multiple sclerosis. A randomized controlled pilot study. *Mult Scler Relat Dis* 2018; 19: 25–29.

[338] Gandolfi M, Valè N, Dimitrova EK, et al. Effects of High-intensity Robot-assisted Hand Training on Upper Limb Recovery and Muscle Activity in Individuals With Multiple Sclerosis: A Randomized, Controlled, Single-Blinded Trial. *Front Neurol* 2018; 9: 905.

[339] Fox EJ, Markowitz C, Applebee A, et al. Ocrelizumab reduces progression of upper extremity impairment in patients with primary progressive multiple sclerosis: Findings from the phase III randomized ORATORIO trial. *Multiple Scler Houndmills Basingstoke Engl* 2018; 24: 1862–1870.

[340] Jonsdottir J, Perini G, Ascolese A, et al. Unilateral arm rehabilitation for persons with multiple sclerosis using serious games in a virtual reality approach: Bilateral treatment effect? *Mult Scler Relat Dis* 2019; 35: 76–82.

[341] Marion S, Leonid C, Belinda B, et al. Effects of modified-release fampridine on upper limb impairment in patients with Multiple Sclerosis. *Mult Scler Relat Dis* 2020; 40: 101971.

[342] Cuesta-Gómez A, Sánchez-Herrera-Baeza P, Oña-Simbaña ED, et al. Effects of virtual reality associated with serious games for upper limb rehabilitation inpatients with multiple sclerosis: randomized controlled trial. *J Neuroeng Rehabil* 2020; 17: 90.

[343] Solaro C, Sire A, Uccelli MM, et al. Efficacy of levetiracetam on upper limb movement in multiple sclerosis patients with cerebellar signs: a multicenter double‐blind, placebo‐controlled, crossover study. *Eur J Neurol* 2020; 27: 2209–2216.

[344] Saini A, Zucker-Levin A, McMillan B, et al. A Descriptive Correlational Study to Evaluate Three Measures of Assessing Upper Extremity Function in Individuals with Multiple Sclerosis. *Multiple Scler Int* 2021; 2021: 5588335.

[345] Giordano A, Clarelli F, Cannizzaro M, et al. BDNF Val66Met Polymorphism Is Associated With Motor Recovery After Rehabilitation in Progressive Multiple Sclerosis Patients. *Front Neurol* 2022; 13: 790360.

[346] Alexander S, Peryer G, Gray E, et al. Wearable technologies to measure clinical outcomes in multiple sclerosis: A scoping review. *Multiple Scler Houndmills Basingstoke Engl* 2021; 27: 1643–1656.

[347] Cheng W-Y, Bourke AK, Lipsmeier F, et al. U-turn speed is a valid and reliable smartphone-based measure of multiple sclerosis-related gait and balance impairment. *Gait Posture* 2021; 84: 120–126.

[348] Polhemus A, Ortiz LD, Brittain G, et al. Walking on common ground: a cross-disciplinary scoping review on the clinical utility of digital mobility outcomes. *Npj Digital Medicine* 2021; 4: 149.

[349] Zhai Y, Nasseri N, Pöttgen J, et al. Smartphone Accelerometry: A Smart and Reliable Measurement of Real-Life Physical Activity in Multiple Sclerosis and Healthy Individuals. *Front Neurol* 2020; 11: 688.

[350] Carpinella I, Gervasoni E, Anastasi D, et al. Instrumentally assessed gait quality is more relevant than gait endurance and velocity to explain patient‐reported walking ability in early‐stage multiple sclerosis. *Eur J Neurol* 2021; 28: 2259–2268.

[351] Pilloni G, Choi C, Shaw MT, et al. Walking in multiple sclerosis improves with tDCS: a randomized, double‐blind, sham‐controlled study. *Ann Clin Transl Neur* 2020; 7: 2310–2319.

[352] Krysko KM, Akhbardeh A, Arjona J, et al. Biosensor vital sign detects multiple sclerosis progression. *Ann Clin Transl Neur* 2021; 8: 4–14.

[353] Chung KK, Altmann D, Barkhof F, et al. A 30‐Year Clinical and Magnetic Resonance Imaging Observational Study of Multiple Sclerosis and Clinically Isolated Syndromes. *Annals of Neurology* 2020; 87: 63–74.

[354] Mamoei S, Jensen HB, Dalgas U, et al. A cross-sectional comparison of performance, neurophysiological and MRI outcomes of responders and non-responders to fampridine treatment in multiple sclerosis – An explorative study. *Journal of Clinical Neuroscience* 2020; 82: 179–185.

[355] Pietrzak A, Kalinowska-Łyszczarz A, Osztynowicz K, et al. A long-term follow-up study on biochemical and clinical biomarkers of response to interferon beta-1b treatment in relapsing-remitting multiple sclerosis. *Advances in clinical and experimental medicine : official organ Wroclaw Medical University* 2020; 29: 841–851.

[356] Gold J, Marta M, Meier UC, et al. A phase II baseline versus treatment study to determine the efficacy of raltegravir (Isentress) in preventing progression of relapsing remitting multiple sclerosis as determined by gadolinium-enhanced MRI: The INSPIRE study. *Multiple Sclerosis and Related Disorders* 2018; 24: 123–128.

[357] Giovannoni G, Knappertz V, Steinerman JR, et al. A randomized, placebo-controlled, phase 2 trial of laquinimod in primary progressive multiple sclerosis. *Neurology* 2019; 95: e1027–e1040.

[358] Bose G, Rush C, Atkins HL, et al. A real-world single-centre analysis of alemtuzumab and cladribine for multiple sclerosis. *Multiple Sclerosis and Related Disorders* 2021; 52: 102945.

[359] Zivadinov R, Horakova D, Bergsland N, et al. A Serial 10-Year Follow-Up Study of Atrophied Brain Lesion Volume and Disability Progression in Patients with Relapsing-Remitting MS. *American Journal of Neuroradiology* 2019; 40: 446–452.

[360] Häußler V, Ufer F, Pöttgen J, et al. aHSCT is superior to alemtuzumab in maintaining NEDA and improving cognition in multiple sclerosis. *Annals of Clinical and Translational Neurology* 2021; 8: 1269–1278.

[361] Havrdova E, Arnold DL, Cohen JA, et al. Alemtuzumab CARE-MS I 5-year follow-up. *Neurology* 2017; 89: 1107–1116.

[362] Coles AJ, Cohen JA, Fox EJ, et al. Alemtuzumab CARE-MS II 5-year follow-up: Efficacy and safety findings. *Neurology* 2017; 89: 1117–1126.

[363] Sprenger T, Kappos L, Radue E-W, et al. Association of brain volume loss and long-term disability outcomes in patients with multiple sclerosis treated with teriflunomide. *Multiple Sclerosis Journal* 2019; 26: 1207–1216.

[364] Bass AD, Arroyo R, Boster AL, et al. Alemtuzumab outcomes by age: Post hoc analysis from the randomized CARE-MS studies over 8 years. *Multiple Sclerosis and Related Disorders* 2021; 49: 102717.

[365] Zimmermann HG, Knier B, Oberwahrenbrock T, et al. Association of Retinal Ganglion Cell Layer Thickness With Future Disease Activity in Patients With Clinically Isolated Syndrome. *JAMA Neurology* 2018; 75: 1071–1079.

[366] Szilasiova J, Mikula P, Rosenberger J, et al. Associations between neurofilament light chain levels, disease activity and brain atrophy in progressive multiple sclerosis. *Biomedical Papers* 2021; 166: 304–311.

[367] Martínez-Lapiscina EH, Mahatanan R, Lee C-H, et al. Associations of serum 25(OH) vitamin D levels with clinical and radiological outcomes in multiple sclerosis, a systematic review and meta-analysis. *Journal of the Neurological Sciences* 2020; 411: 116668.

[368] Nicholas RS, Rhone EE, Mariottini A, et al. Autologous Hematopoietic Stem Cell Transplantation in Active Multiple Sclerosis: A Real-world Case Series. *Neurology* 2020; 97: e890–e901.

[369] Rojas JI, Sanchez F, Caro F, et al. Brain volume loss and no evidence of disease activity over 3 years in multiple sclerosis patients under interferon beta 1a subcutaneous treatment. *Journal of Clinical Neuroscience* 2019; 59: 175–178.

[370] Yokote H, Kamata T, Toru S, et al. Brain volume loss is present in Japanese multiple sclerosis patients with no evidence of disease activity. *Neurological Sciences* 2018; 39: 1713–1716.

[371] Novakova L, Axelsson M, Khademi M, et al. Cerebrospinal fluid biomarkers as a measure of disease activity and treatment efficacy in relapsing‐remitting multiple sclerosis. *Journal of Neurochemistry* 2017; 141: 296–304.

[372] Bartosik-Psujek H, Kaczyński Ł, Górecka M, et al. Cladribine tablets versus other disease-modifying oral drugs in achieving no evidence of disease activity (NEDA) in multiple sclerosis–A systematic review and network meta-analysis. *Multiple Sclerosis and Related Disorders* 2021; 49: 102769.

[373] Anderson A, Krysko KM, Rutatangwa A, et al. Clinical and Radiologic Disease Activity in Pregnancy and Postpartum in MS. *Neurology® Neuroimmunology & Neuroinflammation* 2021; 8: e959.

[374] Smoot K, Chen C, Stuchiner T, et al. Clinical outcomes of patients with multiple sclerosis treated with ocrelizumab in a US community MS center: an observational study. *BMJ Neurology Open* 2021; 3: e000108.

[375] D’Amico E, Zanghì A, Callari G, et al. Comparable efficacy and safety of dimethyl fumarate and teriflunomide treatment in Relapsing-Remitting Multiple Sclerosis: an Italian real-word multicenter experience. *Therapeutic Advances in Neurological Disorders* 2018; 11: 1756286418796404.

[376] Mamoei S, Jensen HB, Pedersen AK, et al. Clinical, Neurophysiological, and MRI Markers of Fampridine Responsiveness in Multiple Sclerosis—An Explorative Study. *Frontiers in Neurology* 2021; 12: 758710.

[377] Boz C, Terzi M, Özer B, et al. Comparative analysis of fingolimod versus teriflunomide in relapsing–remitting multiple sclerosis. *Multiple Sclerosis and Related Disorders* 2019; 36: 101376.

[378] Rollot F, Couturier J, Casey R, et al. Comparative Effectiveness of Natalizumab Versus Anti-CD20 in Highly Active Relapsing–Remitting Multiple Sclerosis After Fingolimod Withdrawal. *Neurotherapeutics* 2022; 19: 476–490.

[379] Laplaud D-A, Casey R, Barbin L, et al. Comparative effectiveness of teriflunomide vs dimethyl fumarate in multiple sclerosis. *Neurology* 2019; 93: e635–e646.

[380] Hersh CM. Comparative efficacy and discontinuation of dimethyl fumarate and fingolimod in clinical practice at 24-month follow-up. *Multiple Sclerosis Journal – Experimental, Translational and Clinical* 2017; 3: 2055217317715485.

[381] Sattarnezhad N, Healy BC, Baharnoori M, et al. Comparison of dimethyl fumarate and interferon outcomes in an MS cohort. *BMC Neurology* 2022; 22: 252.

[382] Foley JF, Defer G, Ryerson LZ, et al. Comparison of switching to 6-week dosing of natalizumab versus continuing with 4-week dosing in patients with relapsing-remitting multiple sclerosis (NOVA): a randomised, controlled, open-label, phase 3b trial. *The Lancet Neurology* 2022; 21: 608–619.

[383] Comi G, Dadon Y, Sasson N, et al. CONCERTO: A randomized, placebo-controlled trial of oral laquinimod in relapsing-remitting multiple sclerosis. *Multiple Sclerosis Journal* 2021; 28: 608–619.

[384] Kolčava J, Kočica J, Hulová M, et al. Conversion of clinically isolated syndrome to multiple sclerosis: a prospective study. *Multiple Sclerosis and Related Disorders* 2020; 44: 102262.

[385] Haider L, Prados F, Chung K, et al. Cortical involvement determines impairment 30 years after a clinically isolated syndrome. *Brain* 2021; 144: awab033-.

[386] Høgestøl EA, Kaufmann T, Nygaard GO, et al. Cross-Sectional and Longitudinal MRI Brain Scans Reveal Accelerated Brain Aging in Multiple Sclerosis. *Frontiers in Neurology* 2019; 10: 450.

[387] Masanneck L, Rolfes L, Regner-Nelke L, et al. Detecting ongoing disease activity in mildly affected multiple sclerosis patients under first-line therapies. *Multiple Sclerosis and Related Disorders* 2022; 63: 103927.

[388] Wu X, Xue T, Wang Z, et al. Different Doses of Fingolimod in Relapsing-Remitting Multiple Sclerosis: A Systematic Review and Meta-Analysis of Randomized Controlled Trials. *Frontiers in Pharmacology* 2021; 12: 621856.

[389] Chow HH, Talbot J, Lundell H, et al. Dimethyl Fumarate Treatment in Patients With Primary Progressive Multiple Sclerosis: A Randomized, Controlled Trial. *Neurology(R) neuroimmunology & neuroinflammation* 2021; 8: e1037.

[390] Tsantes E, Curti E, Ferraro D, et al. Dimethyl fumarate‐induced lymphocyte count drop is related to clinical effectiveness in relapsing–remitting multiple sclerosis. *European Journal of Neurology* 2021; 28: 269–277.

[391] Río J, Rovira À, Tintoré M, et al. Disability progression markers over 6–12 years in interferon-β-treated multiple sclerosis patients. *Multiple Sclerosis Journal* 2017; 24: 322–330.

[392] Vollmer B, Ontaneda D, Bandyopadhyay A, et al. Discontinuation and comparative effectiveness of dimethyl fumarate and fingolimod in 2 centers. *Neurology: Clinical Practice* 2018; 8: 292–301.

[393] Yano H, Gonzalez C, Healy BC, et al. Discontinuation of disease-modifying therapy for patients with relapsing-remitting multiple sclerosis: Effect on clinical and MRI outcomes. *Multiple Sclerosis and Related Disorders* 2019; 35: 119–127.

[394] Hua LH, Harris H, Conway D, et al. Disease activity outcomes with different washout periods after switching from natalizumab to an alternative disease-modifying therapy. *Journal of Neurology* 2020; 267: 2214–2220.

[395] Liu Z, Liao Q, Wen H, et al. Disease modifying therapies in relapsing-remitting multiple sclerosis: A systematic review and network meta-analysis. *Autoimmunity Reviews* 2021; 20: 102826.

[396] Manzano GS, Holroyd KB, Kaplan T, et al. Disease modifying therapy management of multiple sclerosis after stem cell therapies: A retrospective case series. *Multiple Sclerosis and Related Disorders* 2022; 63: 103861.

[397] Riancho J, Setien S, Torre JRS de la, et al. Does Extended Interval Dosing Natalizumab Preserve Effectiveness in Multiple Sclerosis? A 7 Year-Retrospective Observational Study. *Frontiers in Immunology* 2021; 12: 614715.

[398] Ferrè L, Mogavero A, Clarelli F, et al. Early evidence of disease activity during fingolimod predicts medium-term inefficacy in relapsing-remitting multiple sclerosis. *Multiple Sclerosis Journal* 2020; 27: 1374–1383.

[399] Simonsen CS, Flemmen HØ, Broch L, et al. Early High Efficacy Treatment in Multiple Sclerosis Is the Best Predictor of Future Disease Activity Over 1 and 2 Years in a Norwegian Population-Based Registry. *Frontiers in Neurology* 2021; 12: 693017.

[400] Brownlee WJ, Altmann DR, Prados F, et al. Early imaging predictors of long-term outcomes in relapse-onset multiple sclerosis. *Brain* 2019; 142: 2276–2287.

[401] Gajamange S. Early imaging predictors of longer term multiple sclerosis risk and severity in acute optic neuritis. *Multiple Sclerosis Journal – Experimental, Translational and Clinical* 2019; 5: 2055217319863122.

[402] Freedman MS. Early MRI outcomes in participants with a first clinical demyelinating event at risk of multiple sclerosis in the ORACLE-MS study. *Multiple Sclerosis Journal – Experimental, Translational and Clinical* 2021; 7: 2055217321990852.

[403] Coyle PK, Reder AT, Freedman MS, et al. Early MRI results and odds of attaining ‘no evidence of disease activity’ status in MS patients treated with interferon β-1a in the EVIDENCE study. *Journal of the Neurological Sciences* 2017; 379: 151–156.

[404] Wawrzyniak S, Koziarska D, Kułakowska A, et al. Early predictors of injectable disease modifying drugs suboptimal response based on clinical and radiological data assessment in Polish Multiple Sclerosis patients. *Neurologia i Neurochirurgia Polska* 2018; 53: 131–137.

[405] Newsome SD, Scott TF, Arnold DL, et al. Early treatment responses to peginterferon beta-1a are associated with longer-term clinical outcomes in patients with relapsing-remitting multiple sclerosis: Subgroup analyses of ADVANCE and ATTAIN. *Multiple Sclerosis and Related Disorders* 2022; 57: 103367.

[406] Havrdova E, Giovannoni G, Gold R, et al. Effect of delayed-release dimethyl fumarate on no evidence of disease activity in relapsing-remitting multiple sclerosis: integrated analysis of the phase III DEFINE and CONFIRM studies. *European Journal of Neurology* 2017; 24: 726–733.

[407] Crescenzo F, Marastoni D, Zuco C, et al. Effect of glatiramer acetate on cerebral grey matter pathology in patients with relapsing-remitting multiple sclerosis. *Multiple sclerosis and related disorders* 2018; 27: 305–311.

[408] Traboulsee A, Li DKB, Cascione M, et al. Effect of interferon beta-1a subcutaneously three times weekly on clinical and radiological measures and no evidence of disease activity status in patients with relapsing–remitting multiple sclerosis at year 1. *BMC Neurology* 2018; 18: 143.

[409] Menascu S, Fattal-Valevski A, Vaknin-Dembinsky A, et al. Effect of natalizumab treatment on the rate of No Evidence of Disease Activity in young adults with multiple sclerosis in relation to pubertal stage. *Journal of the Neurological Sciences* 2022; 432: 120074.

[410] Kira J, Nakahara J, Sazonov DV, et al. Effect of ofatumumab versus placebo in relapsing multiple sclerosis patients from Japan and Russia: Phase 2 APOLITOS study. *Multiple Sclerosis Journal* 2021; 28: 1229–1238.

[411] Hersh CM, Harris H, Conway D, et al. Effect of switching from natalizumab to moderate- vs high-efficacy DMT in clinical practice. *Neurology: Clinical Practice* 2020; 10: e53–e65.

[412] Zivadinov R, Bergsland N, Hagemeier J, et al. Effect of teriflunomide on gray and white matter brain pathology in multiple sclerosis using volumetric and diffusion-tensor imaging MRI measures. *Journal of the Neurological Sciences* 2018; 388: 175–181.

[413] Esposito F, Ferrè L, Clarelli F, et al. Effectiveness and baseline factors associated to fingolimod response in a real-world study on multiple sclerosis patients. *Journal of Neurology* 2018; 265: 896–905.

[414] Deslandes MQ, Alves PT, Alvarenga MP, et al. Effectiveness and Adverse Events of Use of Natalizumab in a Brazilian Cohort of Patients With Multiple Sclerosis. *Clinical Therapeutics* 2020; 42: 1292–1301.

[415] Pfeuffer S, Rolfes L, Hackert J, et al. Effectiveness and safety of cladribine in MS: Real-world experience from two tertiary centres. *Multiple Sclerosis Journal* 2021; 28: 257–268.

[416] Alroughani R, Ahmed SF, Behbehani R, et al. Effectiveness and Safety of Dimethyl Fumarate Treatment in Relapsing Multiple Sclerosis Patients: Real-World Evidence. *Neurology and Therapy* 2017; 6: 189–196.

[417] Rojas JI, Patrucco L, Alonso R, et al. Effectiveness and Safety of Early High-Efficacy Versus Escalation Therapy in Relapsing-Remitting Multiple Sclerosis in Argentina. *Clinical Neuropharmacology* 2022; 45: 45–51.

[418] Scotti B, Disanto G, Sacco R, et al. Effectiveness and safety of Rituximab in multiple sclerosis: an observational study from Southern Switzerland. *PLoS ONE* 2018; 13: e0197415.

[419] Bauthman MS. Effectiveness of Anti-Cluster of Differentiation 20 as a Disease-Modifying Therapy in Multiple Sclerosis Across Its Different Phenotypes at the University Hospital of Caen. *Cureus* 2022; 14: e22120.

[420] Rodríguez-Regal A, Ramos-Rúa L, Anibarro-García L, et al. Effectiveness of Dimethyl Fumarate in Real-World Clinical Practice and Strategy to Minimize Adverse Effects and Use of Healthcare Resources. *Patient preference and adherence* 2021; 15: 149–158.

[421] Comi G, Pozzilli C, Morra VB, et al. Effectiveness of fingolimod in real-world relapsing-remitting multiple sclerosis Italian patients: the GENIUS study. *Neurological Sciences* 2020; 41: 2843–2851.

[422] Jaklin AK, Benjaminsen E, Alstadhaug KB. Effectiveness of Natalizumab in Achieving No Evidence of Disease Activity (NEDA-3)—Data From a Local Norwegian Cohort. *Frontiers in Neurology* 2021; 12: 765837.

[423] Naismith RT, Bermel RA, Coffey CS, et al. Effects of Ibudilast on MRI Measures in the Phase 2 SPRINT-MS Study. *Neurology* 2020; 96: e491–e500.

[424] Preziosa P, Rocca MA, Riccitelli GC, et al. Effects of Natalizumab and Fingolimod on Clinical, Cognitive, and Magnetic Resonance Imaging Measures in Multiple Sclerosis. *Neurotherapeutics* 2020; 17: 208–217.

[425] Lorefice L, Fronza M, Fenu G, et al. Effects of Pregnancy and Breastfeeding on Clinical Outcomes and MRI Measurements of Women with Multiple Sclerosis: An Exploratory Real-World Cohort Study. *Neurology and Therapy* 2022; 11: 39–49.

[426] O’Connell K, Sulaimani J, Basdeo SA, et al. Effects of vitamin D3 in clinically isolated syndrome and healthy control participants: A double-blind randomised controlled trial. *Multiple Sclerosis Journal – Experimental, Translational and Clinical* 2017; 3: 2055217317727296.

[427] Cree BAC, Goldman MD, Corboy JR, et al. Efficacy and Safety of 2 Fingolimod Doses vs Glatiramer Acetate for the Treatment of Patients With Relapsing-Remitting Multiple Sclerosis. *JAMA Neurology* 2021; 78: 1–13.

[428] Khan O, Rieckmann P, Boyko A, et al. Efficacy and safety of a three-times-weekly dosing regimen of glatiramer acetate in relapsing–remitting multiple sclerosis patients: 3-year results of the Glatiramer Acetate Low-Frequency Administration open-label extension study. *Multiple Sclerosis Journal* 2016; 23: 818–829.

[429] Kim H, Lee E-J, Kim SK, et al. Efficacy and safety of alemtuzumab in Korean multiple sclerosis patients. *Multiple Sclerosis and Related Disorders* 2019; 30: 247–251.

[430] Okai AF, Amezcua L, Berkovich RR, et al. Efficacy and Safety of Alemtuzumab in Patients of African Descent with Relapsing-Remitting Multiple Sclerosis: 8-Year Follow-up of CARE-MS I and II (TOPAZ Study). *Neurology and therapy* 2019; 8: 367–381.

[431] Ziemssen T, Bass AD, Berkovich R, et al. Efficacy and Safety of Alemtuzumab Through 9 Years of Follow-up in Patients with Highly Active Disease: Post Hoc Analysis of CARE-MS I and II Patients in the TOPAZ Extension Study. *CNS Drugs* 2020; 34: 973–988.

[432] Zmira O, Halpern AI, Abraham L, et al. Efficacy and safety of alemtuzumab treatment in a real-world cohort of patients with multiple sclerosis. *Acta Neurologica Belgica* 2021; 121: 1513–1518.

[433] Fernández Ó, Giovannoni G, Fox RJ, et al. Efficacy and Safety of Delayed-release Dimethyl Fumarate for Relapsing-remitting Multiple Sclerosis in Prior Interferon Users: An Integrated Analysis of DEFINE and CONFIRM. *Clinical Therapeutics* 2017; 39: 1671–1679.

[434] Vermersch P, Oreja‐Guevara C, Siva A, et al. Efficacy and safety of ocrelizumab in patients with relapsing‐remitting multiple sclerosis with suboptimal response to prior disease‐modifying therapies: A primary analysis from the phase 3b CASTING single‐arm, open‐label trial. *European Journal of Neurology* 2022; 29: 790–801.

[435] Cree BAC, Pradhan A, Pei J, et al. Efficacy and safety of ocrelizumab vs interferon beta-1a in participants of African descent with relapsing multiple sclerosis in the Phase III OPERA I and OPERA II studies. *Multiple Sclerosis and Related Disorders* 2021; 52: 103010.

[436] Gärtner J, Hauser SL, Bar-Or A, et al. Efficacy and safety of ofatumumab in recently diagnosed, treatment-naive patients with multiple sclerosis: Results from ASCLEPIOS I and II. *Multiple Sclerosis Journal* 2022; 28: 1562–1575.

[437] Cohen JA, Comi G, Arnold DL, et al. Efficacy and safety of ozanimod in multiple sclerosis: Dose-blinded extension of a randomized phase II study. *Multiple Sclerosis (Houndmills, Basingstoke, England)* 2019; 25: 1255–1262.

[438] Almatrafi YM, Babakkor MA, Irfan M, et al. Efficacy and safety of rituximab in patients with multiple sclerosis. *Neurosciences* 2022; 27: 65–70.

[439] Hartung H-P, Derfuss T, Cree BA, et al. Efficacy and safety of temelimab in multiple sclerosis: Results of a randomized phase 2b and extension study. *Multiple Sclerosis Journal* 2021; 28: 429–440.

[440] Hongell K, Silva DG, Ritter S, et al. Efficacy and safety outcomes in vitamin D supplement users in the fingolimod phase 3 trials. *Journal of Neurology* 2018; 265: 348–355.

[441] Wray S, Bergh FT, Wundes A, et al. Efficacy and Safety Outcomes with Diroximel Fumarate After Switching from Prior Therapies or Continuing on DRF: Results from the Phase 3 EVOLVE-MS-1 Study. *Advances in Therapy* 2022; 39: 1810–1831.

[442] Comi G, Alroughani R, Boster AL, et al. Efficacy of alemtuzumab in relapsing-remitting MS patients who received additional courses after the initial two courses: Pooled analysis of the CARE-MS, extension, and TOPAZ studies. *Multiple Sclerosis (Houndmills, Basingstoke, England)* 2020; 26: 1866–1876.

[443] Wijmeersch BV, Singer BA, Boster A, et al. Efficacy of alemtuzumab over 6 years in relapsing–remitting multiple sclerosis patients who relapsed between courses 1 and 2: Post hoc analysis of the CARE-MS studies. *Multiple Sclerosis (Houndmills, Basingstoke, England)* 2020; 26: 1719–1728.

[444] Vermersch P, Galazka A, Dangond F, et al. Efficacy of cladribine tablets in high disease activity patients with relapsing multiple sclerosis: post hoc analysis of subgroups with and without prior disease-modifying drug treatment. *Current Medical Research and Opinion* 2021; 37: 459–464.

[445] Kondo T, Kawachi I, Onizuka Y, et al. Efficacy of dimethyl fumarate in Japanese multiple sclerosis patients: interim analysis of randomized, double-blind APEX study and its open-label extension. *Multiple Sclerosis Journal - Experimental, Translational and Clinical* 2019; 5: 2055217319864974.

[446] Comi G, Patti F, Rocca MA, et al. Efficacy of fingolimod and interferon beta-1b on cognitive, MRI, and clinical outcomes in relapsing–remitting multiple sclerosis: an 18-month, open-label, rater-blinded, randomised, multicentre study (the GOLDEN study). *Journal of Neurology* 2017; 264: 2436–2449.

[447] Chataway J, Angelis FD, Connick P, et al. Efficacy of three neuroprotective drugs in secondary progressive multiple sclerosis (MS-SMART): a phase 2b, multiarm, double-blind, randomised placebo-controlled trial. *The Lancet Neurology* 2020; 19: 214–225.

[448] Manchon E, Laplaud D, Vukusic S, et al. Efficacy, safety and patient reported outcomes in patients with active relapsing multiple sclerosis treated with ocrelizumab: Final results from the PRO-MSACTIVE study. *Multiple sclerosis and related disorders* 2022; 68: 104109.

[449] Saida T, Kira J, Kishida S, et al. Efficacy, safety, and pharmacokinetics of natalizumab in Japanese multiple sclerosis patients: A double-blind, randomized controlled trial and open-label pharmacokinetic study. *Multiple Sclerosis and Related Disorders* 2017; 11: 25–31.

[450] Rust R, Chien C, Scheel M, et al. Epigallocatechin Gallate in Progressive MS. *Neurology® Neuroimmunology & Neuroinflammation* 2021; 8: e964.

[451] Bellmann-Strobl J, Paul F, Wuerfel J, et al. Epigallocatechin Gallate in Relapsing-Remitting Multiple Sclerosis. *Neurology® Neuroimmunology & Neuroinflammation* 2021; 8: e981.

[452] Wolinsky JS, Montalban X, Hauser SL, et al. Evaluation of no evidence of progression or active disease (NEPAD) in patients with primary progressive multiple sclerosis in the ORATORIO trial. *Annals of Neurology* 2018; 84: 527–536.

[453] Battaglini M, Vrenken H, Brocci RT, et al. Evolution from a first clinical demyelinating event to multiple sclerosis in the REFLEX trial: Regional susceptibility in the conversion to multiple sclerosis at disease onset and its amenability to subcutaneous interferon beta‐1a. *European Journal of Neurology* 2022; 29: 2024–2035.

[454] Vollmer BL, Wolf AB, Sillau S, et al. Evolution of Disease Modifying Therapy Benefits and Risks: An Argument for De-escalation as a Treatment Paradigm for Patients With Multiple Sclerosis. *Frontiers in Neurology* 2022; 12: 799138.

[455] Bellinvia A, Prestipino E, Portaccio E, et al. Experience with rituximab therapy in a real-life sample of multiple sclerosis patients. *Neurological Sciences* 2020; 41: 2939–2945.

[456] Damasceno A, Pimentel-Silva LR, Damasceno BP, et al. Exploring the performance of outcome measures in MS for predicting cognitive and clinical progression in the following years. *Multiple Sclerosis and Related Disorders* 2020; 46: 102513.

[457] Cohen JA, Tenenbaum N, Bhatt A, et al. Extended treatment with fingolimod for relapsing multiple sclerosis: the 14-year LONGTERMS study results. *Therapeutic Advances in Neurological Disorders* 2019; 12: 175628641987832.

[458] Algahtani H, Shirah B, Malik YA, et al. Fingolimod for Relapsing-Remitting Multiple Sclerosis: The Experience From Saudi Arabia. *Clinical neuropharmacology* 2020; 43: 35–38.

[459] Prosperini L, Lucchini M, Haggiag S, et al. Fingolimod vs dimethyl fumarate in multiple sclerosis. *Neurology* 2018; 91: e153–e161.

[460] Zivadinov R, Medin J, Khan N, et al. Fingolimod’s Impact on MRI Brain Volume Measures in Multiple Sclerosis: Results from MS‐MRIUS. *Journal of Neuroimaging* 2018; 28: 399–405.

[461] Hauser SL, Kappos L, Arnold DL, et al. Five years of ocrelizumab in relapsing multiple sclerosis. *Neurology* 2020; 95: e1854–e1867.

[462] Tsantes E, Curti E, Collura F, et al. Five- and seven-year prognostic value of new effectiveness measures (NEDA, MEDA and six-month delayed NEDA) in relapsing-remitting multiple sclerosis. *Journal of the Neurological Sciences* 2020; 414: 116827.

[463] Meca-Lallana JE, Oreja-Guevara C, Muñoz D, et al. Four-year safety and effectiveness data from patients with multiple sclerosis treated with fingolimod: The Spanish GILENYA registry. *PLoS ONE* 2021; 16: e0258437.

[464] Harrer A, Pilz G, Oppermann K, et al. From natalizumab to fingolimod in eight weeks — Immunological, clinical, and radiological data in quest of the optimal switch. *Clinical Immunology* 2017; 176: 87–93.

[465] Zecca C, Merlini A, Disanto G, et al. Half-dose fingolimod for treating relapsing-remitting multiple sclerosis: Observational study. *Multiple Sclerosis Journal* 2017; 24: 167–174.

[466] Vries RM van der V de, Wong YYM, Mescheriakova JY, et al. High neurofilament levels are associated with clinically definite multiple sclerosis in children and adults with clinically isolated syndrome. *Multiple Sclerosis Journal* 2018; 25: 958–967.

[467] Thebault S, Tessier DR, Lee H, et al. High serum neurofilament light chain normalizes after hematopoietic stem cell transplantation for MS. *Neurology® Neuroimmunology & Neuroinflammation* 2019; 6: e598.

[468] Couloume L, Barbin L, Leray E, et al. High-dose biotin in progressive multiple sclerosis: A prospective study of 178 patients in routine clinical practice. *Multiple Sclerosis Journal* 2019; 26: 1898–1906.

[469] Dörr J, Bäcker-Koduah P, Wernecke K-D, et al. High-dose vitamin D supplementation in multiple sclerosis – results from the randomized EVIDIMS (efficacy of vitamin D supplementation in multiple sclerosis) trial. *Multiple Sclerosis Journal - Experimental, Translational and Clinical* 2020; 6: 205521732090347.

[470] Mariottini A, Filippini S, Innocenti C, et al. Impact of autologous haematopoietic stem cell transplantation on disability and brain atrophy in secondary progressive multiple sclerosis. *Multiple Sclerosis Journal* 2019; 27: 61–70.

[471] Koch MW, Mostert J, Repovic P, et al. Impact of clinical outcomes and imaging measures on health-related quality of life in secondary progressive MS. *Multiple Sclerosis Journal* 2021; 28: 1286–1298.

[472] Bose D, Ravi R, Maurya M, et al. Impact of Disease-Modifying Therapies on MRI Outcomes in Patients with Relapsing -Remitting Multiple Sclerosis: A Systematic Review and Network Meta-Analysis. *Multiple Sclerosis and Related Disorders* 2022; 61: 103760.

[473] Zivadinov R, Medin J, Khan N, et al. Impact of fingolimod on clinical and magnetic resonance imaging outcomes in routine clinical practice: A retrospective analysis of the multiple sclerosis, clinical and MRI outcomes in the USA (MS-MRIUS) study. *Multiple Sclerosis and Related Disorders* 2019; 27: 65–73.

[474] Guevara C, Villa E, Diaz V, et al. Inclusion of the Symbol Digit Modalities Test in a revised assessment of ‘no evidence of disease activity-4 (NEDA-4)’ in Latin-American patients with multiple sclerosis. *Multiple Sclerosis and Related Disorders* 2020; 42: 102076.

[475] Lin T-Y, Vitkova V, Asseyer S, et al. Increased Serum Neurofilament Light and Thin Ganglion Cell–Inner Plexiform Layer Are Additive Risk Factors for Disease Activity in Early Multiple Sclerosis. *Neurology® Neuroimmunology & Neuroinflammation* 2021; 8: e1051.

[476] Pantazou V, Pasquier RD, Pot C, et al. Is disease activity prior to fingolimod initiation predictive of response? Fingolimod as a “common” first line treatment. *Revue Neurologique* 2021; 177: 935–940.

[477] Zanghì A, Avolio C, Signoriello E, et al. Is It Time for Ocrelizumab Extended Interval Dosing in Relapsing Remitting MS? Evidence from An Italian Multicenter Experience During the COVID-19 Pandemic. *Neurotherapeutics* 2022; 19: 1535–1545.

[478] D’Amico E, Patti F, Zanghì A, et al. Lateral switch to IFN beta-1a 44 mcg may be effective as escalation switch to fingolimod in selected persons with relapsing remitting multiple sclerosis: a real-world setting experience. *Expert Review of Clinical Pharmacology* 2018; 11: 531–536.

[479] Ghezzi A, Chitnis T, K-Laflamme A, et al. Long-Term Effect of Immediate Versus Delayed Fingolimod Treatment in Young Adult Patients with Relapsing–Remitting Multiple Sclerosis: Pooled Analysis from the FREEDOMS/FREEDOMS II Trials. *Neurology and Therapy* 2019; 8: 461–475.

[480] Bigaut K, Fabacher T, Kremer L, et al. Long-term effect of natalizumab in patients with RRMS: TYSTEN cohort. *Multiple Sclerosis Journal* 2020; 27: 729–741.

[481] Horakova D, Uher T, Krasensky J, et al. Long-term effectiveness of natalizumab on MRI outcomes and no evidence of disease activity in relapsing-remitting multiple sclerosis patients treated in a Czech Republic real-world setting: A longitudinal, retrospective study. *Multiple Sclerosis and Related Disorders* 2020; 46: 102543.

[482] Papeix C, Castelnovo G, Leray E, et al. Long-Term Effectiveness, Safety and Tolerability of Fingolimod in Patients with Multiple Sclerosis in Real-World Treatment Settings in France: The VIRGILE Study. *Neurology and Therapy* 2022; 11: 633–658.

[483] Comi G, Cook S, Rammohan K, et al. Long-term effects of cladribine tablets on MRI activity outcomes in patients with relapsing–remitting multiple sclerosis: the CLARITY Extension study. *Therapeutic Advances in Neurological Disorders* 2018; 11: 1756285617753365.

[484] Gold R, Arnold DL, Bar-Or A, et al. Long-term effects of delayed-release dimethyl fumarate in multiple sclerosis: Interim analysis of ENDORSE, a randomized extension study. *Multiple sclerosis (Houndmills, Basingstoke, England)* 2016; 23: 253–265.

[485] Steingo B, Malik YA, Bass AD, et al. Long-term efficacy and safety of alemtuzumab in patients with RRMS: 12-year follow-up of CAMMS223. *Journal of Neurology* 2020; 267: 3343–3353.

[486] Saida T, Itoyama Y, Kikuchi S, et al. Long-term efficacy and safety of fingolimod in Japanese patients with relapsing multiple sclerosis: 3-year results of the phase 2 extension study. *BMC Neurology* 2017; 17: 17.

[487] Boziki M, Bakirtzis C, Giantzi V, et al. Long-Term Efficacy Outcomes of Natalizumab vs. Fingolimod in Patients With Highly Active Relapsing-Remitting Multiple Sclerosis: Real-World Data From a Multiple Sclerosis Reference Center. *Frontiers in Neurology* 2021; 12: 699844.

[488] Diem L, Nedeltchev K, Kahles T, et al. Long-term evaluation of NEDA-3 status in relapsing-remitting multiple sclerosis patients after switching from natalizumab to fingolimod. *Therapeutic Advances in Neurological Disorders* 2018; 11: 1756286418791103.

[489] Wolinsky JS, Arnold DL, Brochet B, et al. Long-term follow-up from the ORATORIO trial of ocrelizumab for primary progressive multiple sclerosis: a post-hoc analysis from the ongoing open-label extension of the randomised, placebo-controlled, phase 3 trial. *The Lancet Neurology* 2020; 19: 998–1009.

[490] Frau J, Carai M, Coghe G, et al. Long-term follow-up more than 10 years after HSCT: a monocentric experience. *Journal of Neurology* 2018; 265: 410–416.

[491] Lublin FD, Cofield SS, Cutter GR, et al. Long-term follow-up of a randomized study of combination interferon and glatiramer acetate in multiple sclerosis: Efficacy and safety results up to 7 years. *Multiple Sclerosis and Related Disorders* 2017; 18: 95–102.

[492] Guger M, Enzinger C, Leutmezer F, et al. Long-term outcome and predictors of long-term disease activity in natalizumab-treated patients with multiple sclerosis: real life data from the Austrian MS Treatment Registry. *Journal of Neurology* 2021; 268: 4303–4310.

[493] Newsome SD, Scott TF, Arnold DL, et al. Long-term outcomes of peginterferon beta-1a in multiple sclerosis: results from the ADVANCE extension study, ATTAIN. *Therapeutic Advances in Neurological Disorders* 2018; 11: 1756286418791143.

[494] Gold R, Radue E-W, Giovannoni G, et al. Long-term safety and efficacy of daclizumab beta in relapsing–remitting multiple sclerosis: 6-year results from the SELECTED open-label extension study. *Journal of Neurology* 2020; 267: 2851–2864.

[495] Freedman MS, Pozzilli C, Havrdova EK, et al. Long-term Treatment With Ponesimod in Relapsing-Remitting Multiple Sclerosis. *Neurology* 2022; 99: e762–e774.

[496] Hyun J-W, Kim Y, Kim G, et al. Longitudinal analysis of serum neurofilament light chain: A potential therapeutic monitoring biomarker for multiple sclerosis. *Multiple Sclerosis Journal* 2019; 26: 659–667.

[497] Yamamura T, Fukazawa T, Tanaka M, et al. Long‐term, single‐arm, open‐label, multicenter phase 2/4 study of glatiramer acetate by subcutaneous injection in Japanese patients with relapsing–remitting multiple sclerosis. *Clinical and Experimental Neuroimmunology* 2019; 10: 49–56.

[498] Walo-Delgado PE, Maza SS de la, Villarrubia N, et al. Low serum neurofilament light chain values identify optimal responders to dimethyl fumarate in multiple sclerosis treatment. *Scientific Reports* 2021; 11: 9299.

[499] Uher T, Havrdova EK, Benkert P, et al. Measurement of neurofilaments improves stratification of future disease activity in early multiple sclerosis. *Multiple Sclerosis Journal* 2021; 27: 2001–2013.

[500] Calabresi PA, Kappos L, Giovannoni G, et al. Measuring treatment response to advance precision medicine for multiple sclerosis. *Annals of Clinical and Translational Neurology* 2021; 8: 2166–2173.

[501] Camara-Lemarroy C, Metz L, Kuhle J, et al. Minocycline treatment in clinically isolated syndrome and serum NfL, GFAP, and metalloproteinase levels. *Multiple Sclerosis Journal* 2022; 28: 2081–2089.

[502] Houtchens M, Bove R, Healy B, et al. MRI activity in MS and completed pregnancy: Data from a tertiary academic center. *Neurology - Neuroimmunology Neuroinflammation* 2020; 7: e890.

[503] Koch MW, Mostert J, Repovic P, et al. MRI brain volume loss, lesion burden, and clinical outcome in secondary progressive multiple sclerosis. *Multiple Sclerosis Journal* 2021; 28: 561–572.

[504] Rasche L, Scheel M, Otte K, et al. MRI Markers and Functional Performance in Patients With CIS and MS: A Cross-Sectional Study. *Frontiers in Neurology* 2018; 9: 718.

[505] Cellina M, Floridi C, Rosti C, et al. MRI of acute optic neuritis (ON) at the first episode: Can we predict the visual outcome and the development of multiple sclerosis (MS)? *La radiologia medica* 2019; 124: 1296–1303.

[506] Saida T, Kira J-I, Kishida S, et al. Natalizumab for Achieving Relapse-Free, T1 Gadolinium-Enhancing-Lesion-Free, and T2 Lesion-Free Status in Japanese Multiple Sclerosis Patients: A Phase 2 Trial Subanalysis. *Neurology and Therapy* 2017; 6: 153–159.

[507] Perumal J, Balabanov R, Su R, et al. Natalizumab in Early Relapsing-Remitting Multiple Sclerosis: A 4-Year, Open-Label Study. *Advances in Therapy* 2021; 38: 3724–3742.

[508] Algahtani H, Shirah B, Abobaker H, et al. Natalizumab Treatment for Relapsing-Remitting Multiple Sclerosis. *Clinical Neuropharmacology* 2018; 41: 199–201.

[509] Vollmer BL, Nair KV, Sillau S, et al. Natalizumab versus fingolimod and dimethyl fumarate in multiple sclerosis treatment. *Annals of Clinical and Translational Neurology* 2019; 6: 252–262.

[510] Butzkueven H, Licata S, Jeffery D, et al. Natalizumab versus fingolimod for patients with active relapsing-remitting multiple sclerosis: results from REVEAL, a prospective, randomised head-to-head study. *BMJ Open* 2020; 10: e038861.

[511] Puthenparampil M, Cazzola C, Zywicki S, et al. NEDA-3 status including cortical lesions in the comparative evaluation of natalizumab versus fingolimod efficacy in multiple sclerosis. *Therapeutic Advances in Neurological Disorders* 2018; 11: 1756286418805713.

[512] Håkansson I, Tisell A, Cassel P, et al. Neurofilament levels, disease activity and brain volume during follow-up in multiple sclerosis. *Journal of Neuroinflammation* 2018; 15: 209.

[513] Szilasiová J, Rosenberger J, Fedičová M, et al. Neurofilament Light Chain Levels Are Associated with Disease Activity Determined by No Evident Disease Activity in Multiple Sclerosis Patients. *European Neurology* 2021; 84: 272–279.

[514] Kuhle J, Plavina T, Barro C, et al. Neurofilament light levels are associated with long-term outcomes in multiple sclerosis. *Multiple Sclerosis Journal* 2019; 26: 1691–1699.

[515] Ryerson LZ, Naismith RT, Krupp LB, et al. No difference in radiologic outcomes for natalizumab patients treated with extended interval dosing compared with standard interval dosing: Real-world evidence from MS PATHS. *Multiple Sclerosis and Related Disorders* 2022; 58: 103480.

[516] Havrdová E, Arnold DL, Bar-Or A, et al. No evidence of disease activity (NEDA) analysis by epochs in patients with relapsing multiple sclerosis treated with ocrelizumab vs interferon beta-1a. *Multiple Sclerosis Journal – Experimental, Translational and Clinical* 2018; 4: 2055217318760642.

[517] Prosperini L, Annovazzi P, Boffa L, et al. No evidence of disease activity (NEDA-3) and disability improvement after alemtuzumab treatment for multiple sclerosis: a 36-month real-world study. *Journal of Neurology* 2018; 265: 2851–2860.

[518] Kappos L, Havrdova E, Giovannoni G, et al. No evidence of disease activity in patients receiving daclizumab versus intramuscular interferon beta-1a for relapsing-remitting multiple sclerosis in the DECIDE study. *Multiple Sclerosis Journal* 2016; 23: 1736–1747.

[519] Zivadinov R, Khan N, Korn JR, et al. No evidence of disease activity in patients receiving fingolimod at private or academic centers in clinical practice: a retrospective analysis of the multiple sclerosis, clinical, and magnetic resonance imaging outcomes in the USA (MS-MRIUS) study. *Current Medical Research and Opinion* 2018; 34: 1431–1440.

[520] Margoni M, Rinaldi F, Riccardi A, et al. No evidence of disease activity including cognition (NEDA-3 plus) in naïve pediatric multiple sclerosis patients treated with natalizumab. *Journal of Neurology* 2020; 267: 100–105.

[521] Zafar A, AlShamrani FJG. No evidence of disease activity-3 (NEDA-3) status in patients with relapsing remitting multiple sclerosis: Evidence from Saudi cohort receiving mainly Interferon. *Multiple Sclerosis and Related Disorders* 2021; 51: 102875.

[522] Johnsson M, Farman HH, Blennow K, et al. No increase of serum neurofilament light in relapsing-remitting multiple sclerosis patients switching from standard to extended-interval dosing of natalizumab. *Multiple Sclerosis Journal* 2022; 28: 2070–2080.

[523] Turner B, Cree BAC, Kappos L, et al. Ocrelizumab efficacy in subgroups of patients with relapsing multiple sclerosis. *Journal of Neurology* 2019; 266: 1182–1193.

[524] Boziki M, Bakirtzis C, Sintila S-A, et al. Ocrelizumab in Patients with Active Primary Progressive Multiple Sclerosis: Clinical Outcomes and Immune Markers of Treatment Response. *Cells* 2022; 11: 1959.

[525] Weinstock-Guttman B, Bermel R, Cutter G, et al. Ocrelizumab treatment for relapsing-remitting multiple sclerosis after a suboptimal response to previous disease-modifying therapy: A nonrandomized controlled trial. *Multiple Sclerosis (Houndmills, Basingstoke, England)* 2022; 28: 790–800.

[526] Barkhof F, Kappos L, Wolinsky JS, et al. Onset of clinical and MRI efficacy of ocrelizumab in relapsing multiple sclerosis. *Neurology* 2019; 93: 10.1212/WNL.0000000000008189.

[527] Frau J, Saccà F, Signori A, et al. Outcomes after fingolimod to alemtuzumab treatment shift in relapsing–remitting MS patients: a multicentre cohort study. *Journal of Neurology* 2019; 266: 2440–2446.

[528] Perumal J, Fox RJ, Balabanov R, et al. Outcomes of natalizumab treatment within 3 years of relapsing-remitting multiple sclerosis diagnosis: a prespecified 2-year interim analysis of STRIVE. *BMC Neurology* 2019; 19: 116.

[529] Sun Y, Yang Y, Wang Z, et al. Ozanimod for Treatment of Relapsing-Remitting Multiple Sclerosis in Adults: A Systematic Review and Meta-Analysis of Randomized Controlled Trials. *Frontiers in Pharmacology* 2020; 11: 589146.

[530] Arnold DL, Calabresi PA, Kieseier BC, et al. Peginterferon beta-1a improves MRI measures and increases the proportion of patients with no evidence of disease activity in relapsing-remitting multiple sclerosis: 2-year results from the ADVANCE randomized controlled trial. *BMC Neurology* 2017; 17: 29.

[531] Arnold DL, You X, Castrillo-Viguera C. Peginterferon beta-1a reduces the evolution of MRI lesions to black holes in patients with RRMS: a post hoc analysis from the ADVANCE study. *Journal of Neurology* 2017; 264: 1728–1734.

[532] Arnold DL, Shang S, Dong Q, et al. Peginterferon β-1a every 2 weeks increased achievement of no evidence of disease activity over 4 years in the ADVANCE and ATTAIN studies in patients with relapsing–remitting multiple sclerosis. *Therapeutic Advances in Neurological Disorders* 2018; 11: 1756286418795085.

[533] Cree BAC, Arnold DL, Cascione M, et al. Phase IV study of retention on fingolimod versus injectable multiple sclerosis therapies: a randomized clinical trial. *Therapeutic Advances in Neurological Disorders* 2018; 11: 1756286418774338.

[534] Harris S, Comi G, Cree BAC, et al. Plasma neurofilament light chain concentrations as a biomarker of clinical and radiologic outcomes in relapsing multiple sclerosis: Post hoc analysis of Phase 3 ozanimod trials. *European Journal of Neurology* 2021; 28: 3722–3730.

[535] Szilasiová J, Mikula P, Rosenberger J, et al. Plasma neurofilament light chain levels are predictors of disease activity in multiple sclerosis as measured by four-domain NEDA status, including brain volume loss. *Multiple Sclerosis Journal* 2021; 27: 2023–2030.

[536] Kappos L, Fox RJ, Burcklen M, et al. Ponesimod Compared With Teriflunomide in Patients With Relapsing Multiple Sclerosis in the Active-Comparator Phase 3 OPTIMUM Study. *JAMA Neurology* 2021; 78: 1–10.

[537] Sormani MP, Truffinet P, Thangavelu K, et al. Predicting long-term disability outcomes in patients with MS treated with teriflunomide in TEMSO. *Neurology® Neuroimmunology & Neuroinflammation* 2017; 4: e379.

[538] Goodin DS, Reder AT, Traboulsee AL, et al. Predictive validity of NEDA in the 16- and 21-year follow-up from the pivotal trial of interferon beta-1b. *Multiple Sclerosis Journal* 2018; 25: 837–847.

[539] Traboulsee A, Li DKB, Cascione M, et al. Predictive value of early magnetic resonance imaging measures is differentially affected by the dose of interferon beta-1a given subcutaneously three times a week: an exploratory analysis of the PRISMS study. *Bmc Neurol* 2018; 18: 68.

[540] Petracca M, Ruggieri S, Barbuti E, et al. Predictors of Cladribine Effectiveness and Safety in Multiple Sclerosis: A Real-World, Multicenter, 2-Year Follow-Up Study. *Neurology Ther* 2022; 11: 1193–1208.

[541] Cellerino M, Boffa G, Lapucci C, et al. Predictors of Ocrelizumab Effectiveness in Patients with Multiple Sclerosis. *Neurother J Am Soc Exp Neurother* 2021; 18: 2579–2588.

[542] Prosperini L, Ruggieri S, Haggiag S, et al. Prognostic Accuracy of NEDA-3 in Long-term Outcomes of Multiple Sclerosis. *Neurology - Neuroimmunology Neuroinflammation* 2021; 8: e1059.

[543] Ziemssen T, Arnold DL, Alvarez E, et al. Prognostic Value of Serum Neurofilament Light Chain for Disease Activity and Worsening in Patients With Relapsing Multiple Sclerosis: Results From the Phase 3 ASCLEPIOS I and II Trials. *Front Immunol* 2022; 13: 852563.

[544] Pongratz V, Schmidt P, Bussas M, et al. Prognostic value of white matter lesion shrinking in early multiple sclerosis: An intuitive or naïve notion? *Brain Behav* 2019; 9: e01417.

[545] Pato AP, Arpín EC, Regal AR, et al. Progression of a series of patients with relapsing-remitting multiple sclerosis treated for 7 years with natalizumab using the “no evidence of disease activity” parameter. *Neurología (English Edition)* 2021; 36: 346–352.

[546] Petracca M, Quarantelli M, Moccia M, et al. ProspeCtive study to evaluate efficacy, safety and tOlerability of dietary supplemeNT of Curcumin (BCM95) in subjects with Active relapsing MultIple Sclerosis treated with subcutaNeous Interferon beta 1a 44 mcg TIW (CONTAIN): A randomized, controlled trial. *Mult Scler Relat Dis* 2021; 56: 103274.

[547] Hupperts R, Smolders J, Vieth R, et al. Randomized trial of daily high-dose vitamin D3 in patients with RRMS receiving subcutaneous interferon β-1a. *Neurology* 2019; 93: 10.1212/WNL.0000000000008445.

[548] Patrucco L, Cristiano E, Sánchez F, et al. Real-World Effectiveness and Safety of Fingolimod in Patients With Relapsing Remitting Multiple Sclerosis: A Prospective Analysis in Buenos Aires, Argentina. *Clin Neuropharmacol* 2019; 42: 163–166.

[549] Hersh CM, Altincatal A, Belviso N, et al. Real-world effectiveness of dimethyl fumarate versus fingolimod in a cohort of patients with multiple sclerosis using standardized, quantitative outcome metrics. *Multiple Scler J Exp Transl Clin* 2022; 8: 20552173211069852.

[550] Kim KH, Kim S-H, Park NY, et al. Real-World Effectiveness of Natalizumab in Korean Patients With Multiple Sclerosis. *Front Neurol* 2021; 12: 714941.

[551] Ysrraelit MC, Caride A, Sinay V, et al. Real-world effectiveness of natalizumab treatment in patients with relapsing multiple sclerosis in Argentina and Chile. *Arq Neuro-psiquiat* 2021; 79: 407–414.

[552] Mazibrada G, Sharples C, Perfect I. Real-world experience of fingolimod in patients with multiple sclerosis (MS Fine): An observational study in the UK. *Multiple Scler J - Exp Transl Clin* 2018; 4: 205521731880163.

[553] ROJAS JI, PATRUCCO L, FRUNS M, et al. Real-world experience of ocrelizumab in multiple sclerosis patients in Latin America. *Arq Neuro-psiquiat* 2021; 79: 305–309.

[554] Alroughani R, AlKawi Z, Hassan A, et al. Real-world retrospective study of effectiveness and safety of FINgOlimod in relapsing remitting multiple sclerosis in the Middle East and North Africa (FINOMENA). *Clin Neurol Neurosur* 2021; 203: 106576.

[555] Gärtner J, Chitnis T, Ghezzi A, et al. Relapse Rate and MRI Activity in Young Adult Patients With Multiple Sclerosis: A Post Hoc Analysis of Phase 3 Fingolimod Trials. *Multiple Sclerosis Journal – Experimental, Translational and Clinical* 2018; 4: 2055217318778610.

[556] Miscioscia A, Puthenparampil M, Miante S, et al. Retinal inner nuclear layer thinning is decreased and associates with the clinical outcome in ocrelizumab-treated primary progressive multiple sclerosis. *J Neurol* 2022; 269: 5436–5442.

[557] Nygaard GO, Torgauten H, Skattebøl L, et al. Risk of fingolimod rebound after switching to cladribine or rituximab in multiple sclerosis. *Multiple Sclerosis and Related Disorders* 2022; 62: 103812.

[558] Cheshmavar M, Mirmosayyeb O, Badihian N, et al. Rituximab and glatiramer acetate in secondary progressive multiple sclerosis: A randomized clinical trial. *Acta Neurologica Scandinavica* 2021; 143: 178–187.

[559] Vollmer BL, Nair K, Sillau S, et al. Rituximab versus natalizumab, fingolimod, and dimethyl fumarate in multiple sclerosis treatment. *Annals of Clinical and Translational Neurology* 2020; 7: 1466–1476.

[560] Kappos L, Cohan S, Arnold DL, et al. Safety and efficacy of daclizumab beta in patients with relapsing multiple sclerosis in a 5-year open-label study (EXTEND): final results following early termination. *Therapeutic Advances in Neurological Disorders* 2021; 14: 1756286420987941.

[561] Alroughani R, Das R, Penner N, et al. Safety and Efficacy of Delayed-Release Dimethyl Fumarate in Pediatric Patients With Relapsing Multiple Sclerosis (FOCUS). *Pediatric Neurology* 2018; 83: 19–24.

[562] Yavari F, Oliazadeh P, Radfar M, et al. Safety and Efficacy of Fingolimod in Iranian Patients with Relapsing-remitting Multiple Sclerosis. *Basic and Clinical Neuroscience* 2021; 12: 233–242.

[563] Mirabella M, Prosperini L, Lucchini M, et al. Safety and Efficacy of Dimethyl Fumarate in Multiple Sclerosis: An Italian, Multicenter, Real-World Study. *CNS Drugs* 2018; 32: 963–970.

[564] Torgauten HM, Myhr K-M. Safety and efficacy of rituximab as first- and second line treatment in multiple sclerosis – A cohort study. *Multiple Sclerosis Journal – Experimental, Translational and Clinical* 2021; 7: 2055217320973049.

[565] Yamout BI, El-Ayoubi NK, Nicolas J, et al. Safety and Efficacy of Rituximab in Multiple Sclerosis: A Retrospective Observational Study. *Journal of Immunology Research* 2018; 2018: 9084759.

[566] Reich DS, Arnold DL, Vermersch P, et al. Safety and efficacy of tolebrutinib, an oral brain-penetrant BTK inhibitor, in relapsing multiple sclerosis: a phase 2b, randomised, double-blind, placebo-controlled trial. *The Lancet Neurology* 2021; 20: 729–738.

[567] Brune S, Høgestøl EA, Benavent SA de R, et al. Serum neurofilament light chain concentration predicts disease worsening in multiple sclerosis. *Multiple Sclerosis Journal* 2022; 28: 1859–1870.

[568] Reinert M-C, Benkert P, Wuerfel J, et al. Serum neurofilament light chain is a useful biomarker in pediatric multiple sclerosis. *Neurology - Neuroimmunology Neuroinflammation* 2020; 7: e749.

[569] Disanto G, Barro C, Benkert P, et al. Serum Neurofilament light: A biomarker of neuronal damage in multiple sclerosis. *Annals of neurology* 2017; 81: 857–870.

[570] Anderson V, Bentley E, Loveless S, et al. Serum neurofilament-light concentration and real-world outcome in MS. *Journal of the Neurological Sciences* 2020; 417: 117079.

[571] Ceccarelli A, Mifsud V, Abusamra E, et al. Short term real-world Fingolimod efficacy and safety in Emirati patients with multiple sclerosis. *Journal of Clinical Neuroscience* 2020; 71: 39–42.

[572] Samjoo IA, Klotz L, Giovannoni G, et al. Simulated treatment comparison of efficacy outcomes for ofatumumab in ASCLEPIOS I/II versus ocrelizumab in OPERA I/II for the treatment of patients with relapsing multiple sclerosis. *Multiple Sclerosis and Related Disorders* 2022; 66: 104031.

[573] Kappos L, Bar-Or A, Cree BAC, et al. Siponimod versus placebo in secondary progressive multiple sclerosis (EXPAND): a double-blind, randomised, phase 3 study. *Lancet (London, England)* 2017; 391: 1263–1273.

[574] Gold R, Piani-Meier D, Kappos L, et al. Siponimod vs placebo in active secondary progressive multiple sclerosis: a post hoc analysis from the phase 3 EXPAND study. *Journal of Neurology* 2022; 269: 5093–5104.

[575] Yang S, Li X, Wang J, et al. Sphingosine-1-phosphate receptor modulators versus interferon beta for the treatment of relapsing–remitting multiple sclerosis: findings from randomized controlled trials. *Neurological Sciences* 2022; 43: 3565–3581.

[576] Allen-Philbey K, Trane SD, Mao Z, et al. Subcutaneous cladribine to treat multiple sclerosis: experience in 208 patients. *Therapeutic Advances in Neurological Disorders* 2021; 14: 17562864211057660.

[577] Bar-Or A, Grove RA, Austin DJ, et al. Subcutaneous ofatumumab in patients with relapsing-remitting multiple sclerosis: The MIRROR study. *Neurology* 2018; 90: 10.1212/WNL.0000000000005516.

[578] Cree BAC, Bowen JD, Hartung H-P, et al. Subgroup analysis of clinical and MRI outcomes in participants with a first clinical demyelinating event at risk of multiple sclerosis in the ORACLE-MS study. *Multiple Sclerosis and Related Disorders* 2021; 49: 102695.

[579] Hunter SF, Thomas FP, Cascione M, et al. Switching to fingolimod in PREFERMS: Effect of treatment history and naïvety on clinical, MRI and treatment satisfaction outcomes✰. *Multiple Sclerosis and Related Disorders* 2020; 45: 102346.

[580] Calabresi PA, Arnold DL, Sangurdekar D, et al. Temporal profile of serum neurofilament light in multiple sclerosis: Implications for patient monitoring. *Multiple Sclerosis (Houndmills, Basingstoke, England)* 2021; 27: 1497–1505.

[581] Derfuss T, Sastre-Garriga J, Montalban X, et al. The ACROSS study: Long-term efficacy of fingolimod in patients with relapsing–remitting multiple sclerosis. *Multiple Sclerosis Journal – Experimental, Translational and Clinical* 2020; 6: 2055217320907951.

[582] Lie IA, Wesnes K, Kvistad SS, et al. The Effect of Smoking on Long-term Gray Matter Atrophy and Clinical Disability in Patients with Relapsing-Remitting Multiple Sclerosis. *Neurology - Neuroimmunology Neuroinflammation* 2022; 9: e200008.

[583] Kapica-Topczewska K, Tarasiuk J, Collin F, et al. The effectiveness of interferon beta versus glatiramer acetate and natalizumab versus fingolimod in a Polish real-world population. *PLoS ONE* 2019; 14: e0223863.

[584] Abdalla MA, Zakhary CM, Rushdi H, et al. The Effectiveness of Statins as Potential Therapy for Multiple Sclerosis: A Systematic Review of Randomized Controlled trials. *Cureus* 2021; 13: e18092.

[585] Yang T, Tian X, Chen C, et al. The efficacy and safety of fingolimod in patients with relapsing multiple sclerosis: A meta‐analysis. *British Journal of Clinical Pharmacology* 2020; 86: 637–645.

[586] Rouhi F, Mohammadpour Z, Noureini SK, et al. The effects and side effects of laquinimod for the treatment of multiple sclerosis patients: a systematic review and meta-analysis of clinical trials. *European Journal of Clinical Pharmacology* 2020; 76: 611–622.

[587] Vollmer B, Honce JM, Sillau S, et al. The impact of very short transition times on switching from Natalizumab to Fingolimod on imaging and clinical effectiveness outcomes in multiple sclerosis. *Journal of the Neurological Sciences* 2018; 390: 89–93.

[588] Curti E, Tsantes E, Baldi E, et al. The real-world effectiveness of natalizumab and fingolimod in relapsing-remitting multiple sclerosis. An Italian multicentre study. *Multiple Sclerosis and Related Disorders* 2019; 33: 146–152.

[589] Tintore M, Arrambide G, Otero-Romero S, et al. The long-term outcomes of CIS patients in the Barcelona inception cohort: Looking back to recognize aggressive MS. *Multiple Sclerosis (Houndmills, Basingstoke, England)* 2020; 26: 1658–1669.

[590] Alroughani R, Ahmed SF, Behbehani R, et al. The Use of Natalizumab in Pediatric Patients With Active Relapsing Multiple Sclerosis: A Prospective Study. *Pediatric Neurology* 2017; 70: 56–60.

[591] Fuente BP de la, Sabín J, Galán V, et al. Three-Year Effectiveness of Dimethyl Fumarate in Multiple Sclerosis: A Prospective Multicenter Real-World Study. *CNS Drugs* 2020; 34: 1275–1286.

[592] Rowles WM, Hsu W-Y, McPolin K, et al. Transitioning From S1P Receptor Modulators to B Cell–Depleting Therapies in Multiple Sclerosis. *Neurology® Neuroimmunology & Neuroinflammation* 2022; 9: e1183.

[593] Zecca C, Bovis F, Novi G, et al. Treatment of multiple sclerosis with rituximab: A multicentric Italian–Swiss experience. *Multiple Sclerosis Journal* 2019; 26: 1519–1531.

[594] Metz LM, Li DKB, Traboulsee AL, et al. Trial of Minocycline in a Clinically Isolated Syndrome of Multiple Sclerosis. *The New England journal of medicine* 2017; 376: 2122–2133.

[595] Abdel-mannan OA, Manchoon C, Rossor T, et al. Use of Disease-Modifying Therapies in Pediatric Relapsing-Remitting Multiple Sclerosis in the United Kingdom. *Neurology - Neuroimmunology Neuroinflammation* 2021; 8: e1008.

[596] Sempere AP, Berenguer-Ruiz L, Borrego-Soriano I, et al. Ocrelizumab in Multiple Sclerosis: A Real-World Study From Spain. *Front Neurol* 2021; 11: 592304.

[597] Mukhtar H, Yasmeen U, Siddiqa S, et al. Outcomes of Ublituximab Compared to Teriflunomide for Relapsing Multiple Sclerosis: A Meta-Analysis. *Mult Scler Relat Dis* 2022; 65: 104002.

[598] Yousuf F, Dupuy SL, Tauhid S, et al. A two-year study using cerebral gray matter volume to assess the response to fingolimod therapy in multiple sclerosis. *Journal of the Neurological Sciences* 2017; 383: 221–229.

[599] Gisleskog PO, Valenzuela B, Scherz T, et al. An Exposure-Response Analysis of the Clinical Efficacy of Ponesimod in a Randomized Phase II Study in Patients with Multiple Sclerosis. *Clinical Pharmacokinetics* 2021; 60: 1227–1237.

[600] Knier B, Leppenetier G, Wetzlmair C, et al. Association of Retinal Architecture, Intrathecal Immunity, and Clinical Course in Multiple Sclerosis. *JAMA Neurology* 2017; 74: 847–856.

[601] Kuhle J, Kropshofer H, Haering DA, et al. Blood neurofilament light chain as a biomarker of MS disease activity and treatment response. *Neurology* 2019; 92: e1007–e1015.

[602] Petrou P, Kassis I, Levin N, et al. Beneficial effects of autologous mesenchymal stem cell transplantation in active progressive multiple sclerosis. *Brain* 2020; 143: 3574–3588.

[603] Leppert D, Kropshofer H, Häring DA, et al. Blood Neurofilament Light in Progressive Multiple Sclerosis. *Neurology* 2022; 98: e2120–e2131.

[604] Elliott C, Belachew S, Wolinsky JS, et al. Chronic white matter lesion activity predicts clinical progression in primary progressive multiple sclerosis. *Brain* 2019; 142: 2787–2799.

[605] Koch MW, Mostert J, Repovic P, et al. Early first‐line treatment response and subsequent disability worsening in relapsing–remitting multiple sclerosis. *European Journal of Neurology* 2022; 29: 1106–1116.

[606] Wang L, Qi C-H, Zhong R, et al. Efficacy of alemtuzumab and natalizumab in the treatment of different stages of multiple sclerosis patients. *Medicine* 2018; 97: e9908.

[607] McComb M, Krikheli M, Uher T, et al. Neuroprotective associations of apolipoproteins A-I and A-II with neurofilament levels in early multiple sclerosis. *Journal of Clinical Lipidology* 2020; 14: 675-684.e2.

[608] Preziosa P, Pagani E, Moiola L, et al. Occurrence and microstructural features of slowly expanding lesions on fingolimod or natalizumab treatment in multiple sclerosis. *Multiple Sclerosis Journal* 2020; 27: 1520–1532.

[609] Garcia-Cañibano B, Ouanes S, Ganesan GS, et al. Real-world experience of ocrelizumab in multiple sclerosis in an Arab population. *Journal of Drug Assessment* 2021; 10: 106–113.

[610] Airas L, Nylund M, Mannonen I, et al. Rituximab in the treatment of multiple sclerosis in the Hospital District of Southwest Finland. *Multiple Sclerosis and Related Disorders* 2020; 40: 101980.

[611] Brown JWL, Cunniffe NG, Prados F, et al. Safety and efficacy of bexarotene in patients with relapsing-remitting multiple sclerosis (CCMR One): a randomised, double-blind, placebo-controlled, parallel-group, phase 2a study. *The Lancet Neurology* 2021; 20: 709–720.

[612] Lie IA, Kaçar S, Wesnes K, et al. Serum neurofilament as a predictor of 10-year grey matter atrophy and clinical disability in multiple sclerosis: a longitudinal study. *Journal of Neurology, Neurosurgery & Psychiatry* 2022; 93: 849–857.

[613] Kuhle J, Nourbakhsh B, Grant D, et al. Serum neurofilament is associated with progression of brain atrophy and disability in early MS. *Neurology* 2017; 88: 826–831.

[614] Srpova B, Uher T, Hrnciarova T, et al. Serum neurofilament light chain reflects inflammation-driven neurodegeneration and predicts delayed brain volume loss in early stage of multiple sclerosis. *Multiple Sclerosis Journal* 2019; 27: 52–60.

[615] Magliozzi R, Scalfari A, Pisani AI, et al. The CSF Profile Linked to Cortical Damage Predicts Multiple Sclerosis Activity. *Annals of Neurology* 2020; 88: 562–573.

[616] Lorefice L, Destro F, Fenu G, et al. The impact of modifiable risk factors on lesion burden in patients with early multiple sclerosis. *Multiple Sclerosis and Related Disorders* 2020; 39: 101886.

[617] Williams T, Heslegrave A, Zetterberg H, et al. The prognostic significance of early blood neurofilament light chain concentration and magnetic resonance imaging variables in relapse‐onset multiple sclerosis. *Brain and Behavior* 2022; 12: e2700.

[618] Izquierdo G, Damas F, Páramo MD, et al. The real-world effectiveness and safety of fingolimod in relapsing-remitting multiple sclerosis patients: An observational study. *PLoS ONE* 2017; 12: e0176174.

[619] Plavina T, Singh CM, Sangurdekar D, et al. Association of Serum Neurofilament Light Levels With Long-term Brain Atrophy in Patients With a First Multiple Sclerosis Episode. *JAMA Network Open* 2020; 3: e2016278.

[620] Lamancová P, Urban P, Mašlanková J, et al. Correlation of selected serum protein levels with the degree of disability and NEDA-3 status in multiple sclerosis phenotypes. *European review for medical and pharmacological sciences* 2022; 26: 3933–3941.

[621] Rice J, Hildebrand A, Spain R, et al. A cross-sectional survey of cannabis use by people with MS in Oregon and Southwest Washington. *Mult Scler Relat Dis* 2021; 55: 103172.

[622] Lorefice L, Frau J, Coghe G, et al. Assessing the burden of vascular risk factors on brain atrophy in multiple sclerosis: A case- control MRI study. *Multiple Sclerosis and Related Disorders* 2019; 27: 74–78.

[623] Weinstock-Guttman B, Medin J, Khan N, et al. Assessing ‘No Evidence of Disease Activity’ Status in Patients with Relapsing-Remitting Multiple Sclerosis Receiving Fingolimod in Routine Clinical Practice: A Retrospective Analysis of the Multiple Sclerosis Clinical and Magnetic Resonance Imaging Outcomes in the USA (MS-MRIUS) Study. *CNS Drugs* 2018; 32: 75–84.

[624] Cantó E, Barro C, Zhao C, et al. Association Between Serum Neurofilament Light Chain Levels and Long-term Disease Course Among Patients With Multiple Sclerosis Followed up for 12 Years. *JAMA Neurology* 2019; 76: 1359.

[625] Cagol A, Schaedelin S, Barakovic M, et al. Association of Brain Atrophy With Disease Progression Independent of Relapse Activity in Patients With Relapsing Multiple Sclerosis. *JAMA Neurology* 2022; 79: 682–692.

[626] Mowry EM, Azevedo CJ, McCulloch CE, et al. Body mass index, but not vitamin D status, is associated with brain volume change in MS. *Neurology* 2018; 91: e2256–e2264.

[627] Vidal-Jordana A, Sastre-Garriga J, Pareto D, et al. Brain atrophy 15 years after CIS: Baseline and follow-up clinico-radiological correlations. *Multiple Sclerosis Journal* 2017; 24: 721–727.

[628] Honce JM, Nair KV, Hoyt BD, et al. Brain Atrophy Rates for Stable Multiple Sclerosis Patients on Long-Term Fingolimod versus Glatiramer Acetate. *Frontiers in Neurology* 2020; 11: 1045.

[629] Alvarez E, Nair KV, Hoyt BD, et al. Brain atrophy rates in patients with multiple sclerosis on long term natalizumab resembles healthy controls. *Multiple Sclerosis and Related Disorders* 2021; 55: 103170.

[630] Kjølhede T, Siemonsen S, Wenzel D, et al. Can resistance training impact MRI outcomes in relapsing-remitting multiple sclerosis? *Multiple Sclerosis Journal* 2017; 24: 1356–1365.

[631] Macaron G, Baldassari LE, Nakamura K, et al. Cognitive processing speed in multiple sclerosis clinical practice: association with patient‐reported outcomes, employment and magnetic resonance imaging metrics. *European Journal of Neurology* 2020; 27: 1238–1249.

[632] Uher T, Vaneckova M, Sobisek L, et al. Combining clinical and magnetic resonance imaging markers enhances prediction of 12-year disability in multiple sclerosis. *Multiple Sclerosis Journal* 2016; 23: 51–61.

[633] Abbatemarco JR, Ontaneda D, Nakamura K, et al. Comorbidity effect on processing speed test and MRI measures in multiple sclerosis patients. *Multiple Sclerosis and Related Disorders* 2020; 46: 102593.

[634] Sormani MP, Kappos L, Radue E-W, et al. Defining brain volume cutoffs to identify clinically relevant atrophy in RRMS. *Multiple sclerosis (Houndmills, Basingstoke, England)* 2016; 23: 656–664.

[635] Jakimovski D, Weinstock-Guttman B, Gandhi S, et al. Dietary and lifestyle factors in multiple sclerosis progression: results from a 5-year longitudinal MRI study. *Journal of Neurology* 2019; 266: 866–875.

[636] Galioto R, Berenholz O, Wang Z, et al. Does obesity exacerbate brain lesion volume and atrophy in patients with multiple sclerosis? *Multiple Sclerosis and Related Disorders* 2020; 46: 102502.

[637] Sotirchos ES, Gonzalez-Caldito N, Dewey BE, et al. Effect of disease-modifying therapies on subcortical gray matter atrophy in multiple sclerosis. *Multiple Sclerosis Journal* 2019; 26: 312–321.

[638] Sprenger T, Kappos L, Sormani MP, et al. Effects of teriflunomide treatment on cognitive performance and brain volume in patients with relapsing multiple sclerosis: Post hoc analysis of the TEMSO core and extension studies. *Multiple Sclerosis Journal* 2022; 28: 1719–1728.

[639] Coles AJ, Arnold DL, Bass AD, et al. Efficacy and safety of alemtuzumab over 6 years: final results of the 4-year CARE-MS extension trial. *Therapeutic Advances in Neurological Disorders* 2021; 14: 1756286420982134.

[640] Langeskov-Christensen M, Hvid LG, Nygaard MKE, et al. Efficacy of High-Intensity Aerobic Exercise on Brain MRI Measures in Multiple Sclerosis. *Neurology* 2020; 96: e203–e213.

[641] Gaetano L, Häring DA, Radue E-W, et al. Fingolimod effect on gray matter, thalamus, and white matter in patients with multiple sclerosis. *Neurology* 2018; 90: e1324–e1332.

[642] Cambron M, Mostert J, D’Hooghe M, et al. Fluoxetine in progressive multiple sclerosis: The FLUOX-PMS trial. *Multiple Sclerosis Journal* 2019; 25: 1728–1735.

[643] Ciampi E, Pareto D, Sastre-Garriga J, et al. Grey matter atrophy is associated with disability increase in natalizumab-treated patients. *Multiple Sclerosis Journal* 2016; 23: 556–566.

[644] Damjanovic D, Valsasina P, Rocca MA, et al. Hippocampal and Deep Gray Matter Nuclei Atrophy Is Relevant for Explaining Cognitive Impairment in MS: A Multicenter Study. *American Journal of Neuroradiology* 2017; 38: 18–24.

[645] SAVŠEK L, STERGAR T, STROJNIK V, et al. IMPACT OF AEROBIC EXERCISE ON CLINICAL AND MAGNETIC RESONANCE IMAGING BIOMARKERS IN PERSONS WITH MULTIPLE SCLEROSIS: AN EXPLORATORY RANDOMIZED CONTROLLED TRIAL. *Journal of Rehabilitation Medicine* 2021; 53: 2772.

[646] Mariottini A, Marchi L, Innocenti C, et al. Intermediate-Intensity Autologous Hematopoietic Stem Cell Transplantation Reduces Serum Neurofilament Light Chains and Brain Atrophy in Aggressive Multiple Sclerosis. *Frontiers in Neurology* 2022; 13: 820256.

[647] Riemenschneider M, Hvid LG, Ringgaard S, et al. Investigating the potential disease-modifying and neuroprotective efficacy of exercise therapy early in the disease course of multiple sclerosis: The Early Multiple Sclerosis Exercise Study (EMSES). *Multiple Sclerosis Journal* 2022; 28: 1620–1629.

[648] Häring DA, Kropshofer H, Kappos L, et al. Long-term prognostic value of longitudinal measurements of blood neurofilament levels. *Neurology - Neuroimmunology Neuroinflammation* 2020; 7: e856.

[649] Conway DS, Planchon SM, Oh SH, et al. Measures of Thalamic Integrity are Associated with Cognitive Functioning in Fingolimod-treated Multiple Sclerosis Patients. *Multiple Sclerosis and Related Disorders* 2021; 47: 102635.

[650] Bernitsas E, Kopinsky H, Lichtman‐Mikol S, et al. Multimodal MRI Response to Fingolimod in Multiple Sclerosis: A Nonrandomized, Single Arm, Observational Study. *Journal of Neuroimaging* 2021; 31: 379–387.

[651] Talmage GD, Coppes OJM, Javed A, et al. Natalizumab stabilizes physical, cognitive, MRI, and OCT markers of disease activity: A prospective, non-randomized pilot study. *PLoS ONE* 2017; 12: e0173299.

[652] Uher T, Vaneckova M, Krasensky J, et al. Pathological cut-offs of global and regional brain volume loss in multiple sclerosis. *Multiple Sclerosis Journal* 2017; 25: 541–553.

[653] Krajnc N, Bsteh G, Kasprian G, et al. Peripheral Hemolysis in Relation to Iron Rim Presence and Brain Volume in Multiple Sclerosis. *Frontiers in Neurology* 2022; 13: 928582.

[654] Stuart CM, Varatharaj A, Domjan J, et al. Physical activity monitoring to assess disability progression in multiple sclerosis. *Multiple Sclerosis Journal - Experimental, Translational and Clinical* 2020; 6: 2055217320975185.

[655] Ziliotto N, Zivadinov R, Baroni M, et al. Plasma levels of protein C pathway proteins and brain magnetic resonance imaging volumes in multiple sclerosis. *European Journal of Neurology* 2020; 27: 235–243.

[656] Colato E, Stutters J, Tur C, et al. Predicting disability progression and cognitive worsening in multiple sclerosis using patterns of grey matter volumes. *J Neurology Neurosurg Psychiatry* 2020; 92: 995–1006.

[657] Tsagkas C, Magon S, Gaetano L, et al. Preferential spinal cord volume loss in primary progressive multiple sclerosis. *Mult Scler J* 2018; 25: 947–957.

[658] Stefano ND, Giorgio A, Battaglini M, et al. Reduced brain atrophy rates are associated with lower risk of disability progression in patients with relapsing multiple sclerosis treated with cladribine tablets. *Multiple Scler Houndmills Basingstoke Engl* 2017; 24: 222–226.

[659] Lev MH, Romero JM, Schwamm LH, et al. Robert H. Ackerman, MD, MPH (1935-2018). *Ajnr Am J Neuroradiol* 2019; 40: E12–E13.

[660] Jakimovski D, Kuhle J, Ramanathan M, et al. Serum neurofilament light chain levels associations with gray matter pathology: a 5‐year longitudinal study. *Annals of Clinical and Translational Neurology* 2019; 6: 1757–1770.

[661] Aktas O, Renner A, Huss A, et al. Serum neurofilament light chain: No clear relation to cognition and neuropsychiatric symptoms in stable MS. *Neurology - Neuroimmunology Neuroinflammation* 2020; 7: e885.

[662] Calvi A, Tur C, Chard D, et al. Slowly expanding lesions relate to persisting black-holes and clinical outcomes in relapse-onset multiple sclerosis. *NeuroImage : Clinical* 2022; 35: 103048.

[663] Cawley N, Tur C, Prados F, et al. Spinal cord atrophy as a primary outcome measure in phase II trials of progressive multiple sclerosis. *Multiple Sclerosis Journal* 2017; 24: 932–941.

[664] Radue E-W, Sprenger T, Gaetano L, et al. Teriflunomide slows BVL in relapsing MS. *Neurology® Neuroimmunology & Neuroinflammation* 2017; 4: e390.

[665] Diaz-Cruz C, Chua AS, Malik MT, et al. The effect of alcohol and red wine consumption on clinical and MRI outcomes in multiple sclerosis. *Multiple Sclerosis and Related Disorders* 2017; 17: 47–53.

[666] Jakimovski D, Weinstock-Guttman B, Hagemeier J, et al. Walking disability measures in multiple sclerosis patients: Correlations with MRI-derived global and microstructural damage. *Journal of the Neurological Sciences* 2018; 393: 128–134.

[667] Hemond CC, Chu R, Tummala S, et al. Whole‐brain atrophy assessed by proportional‐ versus registration‐based pipelines from 3T MRI in multiple sclerosis. *Brain and Behavior* 2018; 8: e01068.

[668] Bose G, Healy BC, Barro C, et al. Younger age at multiple sclerosis onset is associated with worse outcomes at age 50. *Journal of Neurology, Neurosurgery & Psychiatry* 2022; 93: 1112–1119.

[669] Krajnc N, Altmann P, Riedl K, et al. Association of Cerebrospinal Fluid Parameters and Neurofilament Light Chain With Retinal Nerve Fiber Layer Thickness in Multiple Sclerosis. *Frontiers in Neurology* 2022; 13: 814734.

[670] Button J, Al-Louzi O, Lang A, et al. Disease-modifying therapies modulate retinal atrophy in multiple sclerosis. *Neurology* 2017; 88: 525–532.

[671] Shangraw K, Murchison CF, Silbermann E, et al. Effect of Vascular Comorbidity on Visual and Disability Outcomes in a Secondary Progressive Multiple Sclerosis Clinical Trial Cohort. *International journal of MS care* 2022; 24: 169–174.

[672] Schurz N, Sariaslani L, Altmann P, et al. Evaluation of Retinal Layer Thickness Parameters as Biomarkers in a Real-World Multiple Sclerosis Cohort. *Eye and Brain* 2021; 13: 59–69.

[673] Kosa P, Wu T, Phillips J, et al. Idebenone does not inhibit disability progression in primary progressive MS. *Multiple sclerosis and related disorders* 2020; 45: 102434.

[674] Aly L, Havla J, Lepennetier G, et al. Inner retinal layer thinning in radiologically isolated syndrome predicts conversion to multiple sclerosis. *European Journal of Neurology* 2020; 27: 2217–2224.

[675] Andersen MR, Roar M, Sejbaek T, et al. Long-term structural retinal changes in patients with optic neuritis related to multiple sclerosis. *Clinical Ophthalmology (Auckland, NZ)* 2017; 11: 1519–1525.

[676] Chen Y, Larraz J, Wong M, et al. Longitudinal retinal imaging study of newly diagnosed relapsing-remitting multiple sclerosis in Scottish population: baseline and 12 months follow-up profile of FutureMS retinal imaging cohort. *BMJ Open Ophthalmology* 2022; 7: e001024.

[677] Bsteh G, Berek K, Hegen H, et al. Macular ganglion cell–inner plexiform layer thinning as a biomarker of disability progression in relapsing multiple sclerosis. *Multiple Sclerosis Journal* 2020; 27: 684–694.

[678] Tourbah A, Gout O, Vighetto A, et al. MD1003 (High-Dose Pharmaceutical-Grade Biotin) for the Treatment of Chronic Visual Loss Related to Optic Neuritis in Multiple Sclerosis: A Randomized, Double-Blind, Placebo-Controlled Study. *CNS Drugs* 2018; 32: 661–672.

[679] Lambe J, Risher H, Filippatou AG, et al. Modulation of Retinal Atrophy With Rituximab in Multiple Sclerosis. *Neurology* 2021; 96: e2525–e2533.

[680] Capuano R, Zubizarreta I, Alba-Arbalat S, et al. Oligoclonal IgM bands in the cerebrospinal fluid of patients with relapsing MS to inform long-term MS disability. *Multiple Sclerosis Journal* 2021; 27: 1706–1716.

[681] Denis M, Woillez J-P, Smirnov VM, et al. Optic Nerve Lesion Length at the Acute Phase of Optic Neuritis Is Predictive of Retinal Neuronal Loss. *Neurology - Neuroimmunology Neuroinflammation* 2022; 9: e1135.

[682] Outteryck O, Lopes R, Drumez É, et al. Optical coherence tomography for detection of asymptomatic optic nerve lesions in clinically isolated syndrome. *Neurology* 2020; 95: e733–e744.

[683] Bermel RA, Fedler JK, Kaiser P, et al. Optical coherence tomography outcomes from SPRINT-MS, a multicenter, randomized, double-blind trial of ibudilast in progressive multiple sclerosis. *Multiple sclerosis (Houndmills, Basingstoke, England)* 2020; 27: 1384–1390.

[684] Nolan RC, Galetta SL, Frohman TC, et al. Optimal Intereye Difference Thresholds in Retinal Nerve Fiber Layer Thickness for Predicting a Unilateral Optic Nerve Lesion in Multiple Sclerosis. *Journal of Neuro-Ophthalmology* 2018; 38: 451–458.

[685] Bsteh G, Hegen H, Teuchner B, et al. Peripapillary retinal nerve fibre layer as measured by optical coherence tomography is a prognostic biomarker not only for physical but also for cognitive disability progression in multiple sclerosis. *Multiple Sclerosis Journal* 2017; 25: 196–203.

[686] Klumbies K, Rust R, Dörr J, et al. Retinal Thickness Analysis in Progressive Multiple Sclerosis Patients Treated With Epigallocatechin Gallate: Optical Coherence Tomography Results From the SUPREMES Study. *Frontiers in Neurology* 2021; 12: 615790.

[687] Bsteh G, Berek K, Hegen H, et al. Serum neurofilament levels correlate with retinal nerve fiber layer thinning in multiple sclerosis. *Multiple Sclerosis Journal* 2019; 26: 1682–1690.

[688] Zivadinov R, Tavazzi E, Hagemeier J, et al. The Effect of Glatiramer Acetate on Retinal Nerve Fiber Layer Thickness in Patients with Relapsing–Remitting Multiple Sclerosis: A Longitudinal Optical Coherence Tomography Study. *CNS Drugs* 2018; 32: 763–770.

[689] Pietroboni AM, Dell’Arti L, Caprioli M, et al. The loss of macular ganglion cells begins from the early stages of disease and correlates with brain atrophy in multiple sclerosis patients. *Multiple Sclerosis Journal* 2017; 25: 31–38.

[690] Andorrà M, Alba-Arbalat S, Camos-Carreras A, et al. Using Acute Optic Neuritis Trials to Assess Neuroprotective and Remyelinating Therapies in Multiple Sclerosis. *JAMA Neurology* 2020; 77: 234–244.

[691] Bsteh G, Hegen H, Altmann P, et al. Validation of inter-eye difference thresholds in optical coherence tomography for identification of optic neuritis in multiple sclerosis. *Multiple Sclerosis and Related Disorders* 2020; 45: 102403.

[692] Kleerekooper I, Chua S, Foster PJ, et al. Associations of Alcohol Consumption and Smoking With Disease Risk and Neurodegeneration in Individuals With Multiple Sclerosis in the United Kingdom. *JAMA Network Open* 2022; 5: e220902.

[693] Murphy OC, Kalaitzidis G, Vasileiou E, et al. Optical Coherence Tomography and Optical Coherence Tomography Angiography Findings After Optic Neuritis in Multiple Sclerosis. *Frontiers in Neurology* 2020; 11: 618879.

[694] Vasileiou ES, Filippatou AG, Maldonado DP, et al. Socioeconomic disparity is associated with faster retinal neurodegeneration in multiple sclerosis. *Brain : a journal of neurology* 2021; 144: 3664–3673.

[695] Absinta M, Sati P, Masuzzo F, et al. Association of Chronic Active Multiple Sclerosis Lesions With Disability In Vivo. *JAMA Neurology* 2019; 76: 1474–1483.

[696] Treaba CA, Conti A, Klawiter EC, et al. Cortical and phase rim lesions on 7 T MRI as markers of multiple sclerosis disease progression. *Brain Communications* 2021; 3: fcab134.

[697] Absinta M, Sati P, Fechner A, et al. Identification of Chronic Active Multiple Sclerosis Lesions on 3T MRI. *American Journal of Neuroradiology* 2018; 39: 1233–1238.

[698] Dal-Bianco A, Grabner G, Kronnerwetter C, et al. Long-term evolution of multiple sclerosis iron rim lesions in 7 T MRI. *Brain : a journal of neurology* 2020; 144: 833–847.

[699] Dal-Bianco A, Grabner G, Kronnerwetter C, et al. Slow expansion of multiple sclerosis iron rim lesions: pathology and 7 T magnetic resonance imaging. *Acta Neuropathologica* 2017; 133: 25–42.

[700] Huang W, Sweeney EM, Kaunzner UW, et al. Quantitative susceptibility mapping versus phase imaging to identify multiple sclerosis iron rim lesions with demyelination. *J Neuroimaging* 2022; 32: 667–675.

[701] Williams TE, Holdsworth KP, Nicholas JM, et al. Assessing Neurofilaments as Biomarkers of Neuroprotection in Progressive Multiple Sclerosis. *Neurology: Neuroimmunology & Neuroinflammation* 2022; 9: e1130.

[702] Sormani MP, Haering DA, Kropshofer H, et al. Blood neurofilament light as a potential endpoint in Phase 2 studies in MS. *Annals of Clinical and Translational Neurology* 2019; 6: 1081–1089.

[703] Delcoigne B, Manouchehrinia A, Barro C, et al. Blood neurofilament light levels segregate treatment effects in multiple sclerosis. *Neurology* 2020; 94: e1201–e1212.

[704] Tsagkas C, Naegelin Y, Amann M, et al. Central nervous system atrophy predicts future dynamics of disability progression in a real‐world multiple sclerosis cohort. *European Journal of Neurology* 2021; 28: 4153–4166.

[705] Olesen MN, Soelberg K, Debrabant B, et al. Cerebrospinal fluid biomarkers for predicting development of multiple sclerosis in acute optic neuritis: a population-based prospective cohort study. *Journal of Neuroinflammation* 2019; 16: 59.

[706] Fabis‐Pedrini MJ, Kuhle J, Roberts KMA, et al. Changes in serum neurofilament light chain levels following narrowband ultraviolet B phototherapy in clinically isolated syndrome. *Brain and Behavior* 2022; 12: e2494.

[707] Flon P de, Laurell K, Sundström P, et al. Comparison of plasma and cerebrospinal fluid neurofilament light in a multiple sclerosis trial. *Acta Neurologica Scandinavica* 2019; 139: 462–468.

[708] Christensen JR, Komori M, Essen MR von, et al. CSF inflammatory biomarkers responsive to treatment in progressive multiple sclerosis capture residual inflammation associated with axonal damage. *Multiple Sclerosis Journal* 2018; 25: 937–946.

[709] Reyes S, Smets I, Holden D, et al. CSF neurofilament light chain testing as an aid to determine treatment strategies in MS. *Neurology® Neuroimmunology & Neuroinflammation* 2020; 7: e880.

[710] Sejbaek T, Nielsen HH, Penner N, et al. Dimethyl fumarate decreases neurofilament light chain in CSF and blood of treatment naïve relapsing MS patients. *Journal of neurology, neurosurgery, and psychiatry* 2019; 90: 1324–1330.

[711] Alcalá C, Quintanilla-Bordás C, Gascón F, et al. Effectiveness of rituximab vs. ocrelizumab for the treatment of primary progressive multiple sclerosis: a real-world observational study. *Journal of Neurology* 2022; 269: 3676–3681.

[712] Bock M, Steffen F, Zipp F, et al. Impact of Dietary Intervention on Serum Neurofilament Light Chain in Multiple Sclerosis. *Neurology: Neuroimmunology & Neuroinflammation* 2021; 9: e1102.

[713] Chitnis T, Gonzalez C, Healy BC, et al. Neurofilament light chain serum levels correlate with 10‐year MRI outcomes in multiple sclerosis. *Annals of Clinical and Translational Neurology* 2018; 5: 1478–1491.

[714] Bhan A, Jacobsen C, Myhr KM, et al. Neurofilaments and 10-year follow-up in multiple sclerosis. *Multiple sclerosis (Houndmills, Basingstoke, England)* 2018; 24: 1301–1307.

[715] Pauwels A, Schependom JV, Devolder L, et al. Plasma glial fibrillary acidic protein and neurofilament light chain in relation to disability worsening in multiple sclerosis. *Multiple Sclerosis Journal* 2022; 28: 1685–1696.

[716] Galetta K, Deshpande C, Healy BC, et al. Serum neurofilament levels and patient‐reported outcomes in multiple sclerosis. *Annals of Clinical and Translational Neurology* 2021; 8: 631–638.

[717] Bjornevik K, Munger KL, Cortese M, et al. Serum Neurofilament Light Chain Levels in Patients With Presymptomatic Multiple Sclerosis. *JAMA Neurology* 2020; 77: 58–64.

[718] Thebault S, Abdoli M, Fereshtehnejad S-M, et al. Serum neurofilament light chain predicts long term clinical outcomes in multiple sclerosis. *Scientific reports* 2019; 10: 10381.

[719] Bsteh G, Berek K, Hegen H, et al. Serum neurofilament light levels correlate with change of olfactory function in multiple sclerosis. *Multiple Sclerosis Journal - Experimental, Translational and Clinical* 2019; 5: 2055217319885987.

[720] Wendel E-M, Bertolini A, Kousoulos L, et al. Serum neurofilament light-chain levels in children with monophasic myelin oligodendrocyte glycoprotein-associated disease, multiple sclerosis, and other acquired demyelinating syndrome. *Multiple Sclerosis Journal* 2022; 28: 1553–1561.

[721] Lokhande H, Rosso M, Tauhid S, et al. Serum NfL levels in the first five years predict 10-year thalamic fraction in patients with MS. *Multiple Sclerosis Journal – Experimental, Translational and Clinical* 2022; 8: 20552173211069348.

[722] Novakova L, Axelsson M, Khademi M, et al. Cerebrospinal fluid biomarkers of inflammation and degeneration as measures of fingolimod efficacy in multiple sclerosis. *Multiple sclerosis (Houndmills, Basingstoke, England)* 2016; 23: 62–71.

[723] Busse M, Playle R, Latchem-Hastings J, et al. A web-based life-style, exercise and activity intervention for people with progressive multiple sclerosis: Results of a single-arm feasibility study. *Mult Scler Relat Dis* 2022; 57: 103388.

[724] Buttolph L, Corn J, Hanes D, et al. Community qigong for People with Multiple Sclerosis: A Pragmatic Feasibility Study. *J Altern Complementary Medicine* 2021; 27: 506–514.

[725] Freeman J, Hendrie W, Jarrett L, et al. Assessment of a home-based standing frame programme in people with progressive multiple sclerosis (SUMS): a pragmatic, multi-centre, randomised, controlled trial and cost-effectiveness analysis. *Lancet Neurology* 2019; 18: 736–747.

[726] Gold J, Marta M, Meier UC, et al. A phase II baseline versus treatment study to determine the efficacy of raltegravir (Isentress) in preventing progression of relapsing remitting multiple sclerosis as determined by gadolinium-enhanced MRI: The INSPIRE study. *Mult Scler Relat Dis* 2018; 24: 123–128.

[727] Hortobágyi T, Ács P, Baumann P, et al. Comparative Effectiveness of 4 Exercise Interventions Followed by 2 Years of Exercise Maintenance in Multiple Sclerosis: A Randomized Controlled Trial. *Arch Phys Med Rehab* 2022; 103: 1908–1916.

[728] Sand IK, Benn EKT, Fabian M, et al. Randomized-controlled trial of a modified Mediterranean dietary program for multiple sclerosis: A pilot study. *Mult Scler Relat Dis* 2019; 36: 101403.

[729] Krause A, Lee K, König D, et al. Six weeks of whole-body vibration improves fine motor accuracy, functional mobility and quality of life in people with multiple sclerosis. *Plos One* 2022; 17: e0270698.

[730] Lincoln NB, Bradshaw LE, Constantinescu CS, et al. Cognitive rehabilitation for attention and memory in people with multiple sclerosis: a randomized controlled trial (CRAMMS). *Clin Rehabil* 2019; 34: 229–241.

[731] Martini DN, Zeeboer E, Hildebrand A, et al. ADSTEP: Preliminary Investigation of a Multicomponent Walking Aid Program in People With Multiple Sclerosis. *Arch Phys Med Rehab* 2018; 99: 2050–2058.

[732] Plow M, Motl RW, Finlayson M, et al. Response heterogeneity in a randomized controlled trial of telerehabilitation interventions among adults with multiple sclerosis. *J Telemed Telecare* 2020; 28: 642–652.

[733] Plow M, Finlayson M, Liu J, et al. Randomized Controlled Trial of a Telephone-Delivered Physical Activity and Fatigue Self-management Interventions in Adults With Multiple Sclerosis. *Arch Phys Med Rehab* 2019; 100: 2006–2014.

[734] Sesel A-L, Sharpe L, Beadnall HN, et al. A randomized controlled trial of a web-based mindfulness programme for people with MS with and without a history of recurrent depression. *Mult Scler J* 2022; 28: 1392–1401.

[735] TOLLÁR J, NAGY F, TÓTH BE, et al. Exercise Effects on Multiple Sclerosis Quality of Life and Clinical–Motor Symptoms. *Medicine Sci Sports Exerc* 2019; 52: 1007–1014.

[736] Veldkamp R, Baert I, Kalron A, et al. Structured Cognitive-Motor Dual Task Training Compared to Single Mobility Training in Persons with Multiple Sclerosis, a Multicenter RCT. *J Clin Medicine* 2019; 8: 2177.

[737] Castelnovo G, Gerlach O, Freedman MS, et al. Safety, Patient-Reported Well-Being, and Physician-Reported Assessment of Walking Ability in Patients with Multiple Sclerosis for Prolonged-Release Fampridine Treatment in Routine Clinical Practice: Results of the LIBERATE Study. *Cns Drugs* 2021; 35: 1009–1022.

[738] Chataway J, Angelis FD, Connick P, et al. Efficacy of three neuroprotective drugs in secondary progressive multiple sclerosis (MS-SMART): a phase 2b, multiarm, double-blind, randomised placebo-controlled trial. *Lancet Neurology* 2020; 19: 214–225.

[739] Flon P de, Laurell K, Söderström L, et al. Improved treatment satisfaction after switching therapy to rituximab in relapsing–remitting MS. *Mult Scler J* 2017; 23: 1249–1257.

[740] Hardy TA, Parratt J, Beadnall H, et al. Treatment satisfaction in patients with relapsing-remitting multiple sclerosis initiated on teriflunomide in routine clinical practice: Australian observational data. *Bmj Neurology Open* 2022; 4: e000315.

[741] Liu Y, Vollmer T, Havrdova E, et al. Impact of daclizumab versus interferon beta-1a on patient-reported outcomes in relapsing-remitting multiple sclerosis. *Mult Scler Relat Dis* 2017; 11: 18–24.

[742] Marck CH, Livera AMD, Brown CR, et al. Health outcomes and adherence to a healthy lifestyle after a multimodal intervention in people with multiple sclerosis: Three year follow-up. *Plos One* 2018; 13: e0197759.

[743] Williams J, Moldavskiy M, Bauer K, et al. Safety and Feasibility of Various Functional Electrical Stimulation Cycling Protocols in Individuals With Multiple Sclerosis Who Are Nonambulatory. *Archives Rehabilitation Res Clin Transl* 2020; 2: 100045.

[744] Angelova G, Skodova T, Prokopiusova T, et al. Ambulatory Neuroproprioceptive Facilitation and Inhibition Physical Therapy Improves Clinical Outcomes in Multiple Sclerosis and Modulates Serum Level of Neuroactive Steroids: A Two-Arm Parallel-Group Exploratory Trial. *Life* 2020; 10: 267.

[745] Cree BAC, Goldman MD, Corboy JR, et al. Efficacy and Safety of 2 Fingolimod Doses vs Glatiramer Acetate for the Treatment of Patients With Relapsing-Remitting Multiple Sclerosis. *Jama Neurol* 2021; 78: 1–13.

[746] Dardiotis E, Perpati G, Borsos M, et al. Real-World Assessment of Quality of Life in Patients with Relapsing Remitting Multiple Sclerosis Treated with Teriflunomide for Two Years: Patient-Reported Outcomes from the AURELIO Study in Greece. *Neurology Ther* 2022; 11: 1375–1390.

[747] Kaplan J, Miller T, Baker M, et al. Repository corticotropin injection improves quality metrics in an observational study of multiple sclerosis relapse. *Neurodegener Dis Management* 2021; 11: 469–476.

[748] Kaplan J, Miller T, Baker M, et al. A Prospective Observational Registry of Repository Corticotropin Injection (Acthar® Gel) for the Treatment of Multiple Sclerosis Relapse. *Front Neurol* 2020; 11: 598496.

[749] Learmonth YC, Adamson BC, Kinnett-Hopkins D, et al. Results of a feasibility randomised controlled study of the guidelines for exercise in multiple sclerosis project. *Contemp Clin Trials* 2017; 54: 84–97.

[750] Tanasescu R, Constantinescu CS, Tench CR, et al. Smoking cessation and the reduction of disability progression in Multiple Sclerosis: a cohort study. *Nicotine Tob Res* 2017; 20: ntx084.

[751] Hupperts R, Gasperini C, Lycke J, et al. Efficacy of prolonged-release fampridine versus placebo on walking ability, dynamic and static balance, physical impact of multiple sclerosis, and quality of life: an integrated analysis of MOBILE and ENHANCE. *Ther Adv Neurol Diso* 2022; 15: 17562864221090398.

[752] Turkowitch D, Ludwig R, Nelson E, et al. Telehealth-Delivered Cognitive Behavioral Therapy for Insomnia in Individuals with Multiple Sclerosis: A Pilot Study. *Multiple Scler Int* 2022; 2022: 7110582.

[753] Gunn H, Stevens KN, Creanor S, et al. Balance Right in Multiple Sclerosis (BRiMS): a feasibility randomised controlled trial of a falls prevention programme. *Pilot Feasibility Stud* 2021; 7: 2.

[754] Macdonell R, Nagels G, Laplaud D-A, et al. Improved patient-reported health impact of multiple sclerosis: The ENABLE study of PR-fampridine. *Mult Scler J* 2015; 22: 944–954.

[755] Riazi A, Hobart JC, Lamping DL, et al. Multiple Sclerosis Impact Scale (MSIS-29): reliability and validity in hospital based samples. *J Neurology Neurosurg Psychiatry* 2002; 73: 701.

[756] Zigmond AS, Snaith RP. The hospital anxiety and depression scale. *Acta Psychiat Scand* 1983; 67: 361–70.

[757] Alketbi A, Basit S, Hamza N, et al. The added value of cognition-targeted exercise versus symptom-targeted exercise for multiple sclerosis fatigue: A randomized controlled pilot trial. *Plos One* 2021; 16: e0258752.

[758] Chalah MA, Riachi N, Ahdab R, et al. Effects of left DLPFC versus right PPC tDCS on multiple sclerosis fatigue. *J Neurol Sci* 2017; 372: 131–137.

[759] Coe S, Cossington J, Collett J, et al. A randomised double-blind placebo-controlled feasibility trial of flavonoid-rich cocoa for fatigue in people with relapsing and remitting multiple sclerosis. *J Neurology Neurosurg Psychiatry* 2019; 90: 507.

[760] D’hooghe M, Gassen GV, Kos D, et al. Improving fatigue in multiple sclerosis by smartphone-supported energy management: The MS TeleCoach feasibility study. *Mult Scler Relat Dis* 2018; 22: 90–96.

[761] Fleming KM, Coote SB, Herring MP. Home-based Pilates for symptoms of anxiety, depression and fatigue among persons with multiple sclerosis: An 8-week randomized controlled trial. *Multiple Scler Houndmills Basingstoke Engl* 2021; 27: 2267–2279.

[762] Garjani A, Middleton RM, Nicholas R, et al. Pre-existing anxiety, depression, and neurological disability is associated with long COVID: A prospective and longitudinal cohort of the United Kingdom Multiple Sclerosis Register. *Medrxiv* 2021; 2021.06.25.21259256.

[763] Giovannetti AM, Quintas R, Tramacere I, et al. A resilience group training program for people with multiple sclerosis: Results of a pilot single-blind randomized controlled trial and nested qualitative study. *Plos One* 2020; 15: e0231380.

[764] Hoogerwerf A, Bol Y, Lobbestael J, et al. Mindfulness-based cognitive therapy for severely fatigued multiple sclerosis patients: A waiting list controlled study. *J Rehabil Med* 2017; 49: 497–504.

[765] Hughes AJ, Botanov Y, Beier M. Dialectical behavior therapy skills training for individuals with multiple sclerosis and their support partners: A pilot randomized controlled trial. *Mult Scler Relat Dis* 2022; 59: 103481.

[766] Kleiter I, Lang M, Jeske J, et al. Adherence, satisfaction and functional health status among patients with multiple sclerosis using the BETACONNECT® autoinjector: a prospective observational cohort study. *Bmc Neurol* 2017; 17: 174.

[767] Köpke S, Kasper J, Flachenecker P, et al. Patient education programme on immunotherapy in multiple sclerosis (PEPIMS): a controlled rater-blinded study. *Clin Rehabil* 2016; 31: 250–261.

[768] Krysko KM, Bischof A, Nourbakhsh B, et al. A pilot study of oxidative pathways in MS fatigue: randomized trial of N‐acetyl cysteine. *Ann Clin Transl Neur* 2021; 8: 811–824.

[769] Ladeira F, Mendonça M, Caetano A, et al. Effect of patients’ expectations on clinical response to fampridine treatment. *Neurol Sci* 2019; 40: 175–180.

[770] Mehrabani G, Aminian S, Norton S, et al. Preliminary efficacy of the “SitLess with MS” intervention for changing sedentary behaviour, symptoms, and physical performance in multiple sclerosis. *Disabil Rehabil* 2022; 44: 6374–6381.

[771] Nazari N, Sadeghi M, Ghadampour E, et al. Transdiagnostic treatment of emotional disorders in people with multiple sclerosis: randomized controlled trial. *Bmc Psychology* 2020; 8: 114.

[772] Ozsoy-Unubol T, Ata E, Cavlak M, et al. Effects of Robot-Assisted Gait Training in Patients With Multiple Sclerosis: A Single-Blinded Randomized Controlled Study. *Am J Phys Med Rehab* 2021; 101: 768–774.

[773] Rolf L, Damoiseaux J, Huitinga I, et al. Stress-Axis Regulation by Vitamin D3 in Multiple Sclerosis. *Front Neurol* 2018; 9: 263.

[774] Smyth P, Watson KE, Hamarneh YNA, et al. The effect of nurse practitioner (NP-led) care on health-related quality of life in people with multiple sclerosis – a randomized trial. *Bmc Neurol* 2022; 22: 275.

[775] Tasci I, Demir CF, Bilek F, et al. Physical exercise may improve problem-solving skills and emotional intelligence in patients with relapsing-remitting multiple sclerosis: A cross-sectional study. *Mult Scler Relat Dis* 2022; 59: 103641.

[776] Thomas S, Fazakarley L, Thomas PW, et al. Mii-vitaliSe: a pilot randomised controlled trial of a home gaming system (Nintendo Wii) to increase activity levels, vitality and well-being in people with multiple sclerosis. *Bmj Open* 2017; 7: e016966.

[777] Cavalera C, Rovaris M, Mendozzi L, et al. Online meditation training for people with multiple sclerosis: A randomized controlled trial. *Mult Scler J* 2018; 25: 610–617.

[778] Chen J, Taylor BV, Blizzard L, et al. Effects of multiple sclerosis disease-modifying therapies on employment measures using patient-reported data. *J Neurology Neurosurg Psychiatry* 2018; 89: 1200.

[779] Pöttgen J, Friede T, Lau S, et al. Managing neuropsychological impairment in multiple sclerosis – Controlled study on a standardized metacognitive intervention (MaTiMS). *Mult Scler Relat Dis* 2022; 59: 103687.

[780] Pöttgen J, Moss-Morris R, Wendebourg J-M, et al. Randomised controlled trial of a self-guided online fatigue intervention in multiple sclerosis. *J Neurology Neurosurg Psychiatry* 2018; 89: 970.

[781] Chen J, Diouf I, Taylor BV, et al. Superior effects of natalizumab versus other DMTs on patient-reported outcomes in people with multiple sclerosis. *J Neurology Neurosurg Psychiatry* 2022; 93: 1120–1127.

[782] Hsu W-Y, Anderson A, Rowles W, et al. Effects of melatonin on sleep disturbances in multiple sclerosis: A randomized, controlled pilot study. *Multiple Scler J - Exp Transl Clin* 2021; 7: 20552173211048756.

[783] Rodgers J, Friede T, Vonberg FW, et al. The impact of smoking cessation on multiple sclerosis disease progression. *Brain* 2021; 145: awab385-.

[784] Stimmel MB, Cohen JN, Schneider SJ, et al. A neuropsychologically-based intervention with increased follow-up support for employed women with multiple sclerosis: a pilot randomized controlled trial. *Clin Rehabil* 2020; 34: 1292–1302.

[785] Bouquiaux O, Beaudart C, Thibaut A, et al. Beneficial effects of a supervised and individualized training circuit on physical capacities and quality of life of patients suffering from multiple sclerosis. *Sci Sport* 2022; 37: 468–476.

[786] Rolf L, Muris A-H, Bol Y, et al. Vitamin D3 supplementation in multiple sclerosis: Symptoms and biomarkers of depression. *J Neurol Sci* 2017; 378: 30–35.

[787] Wolf F, Rademacher A, Joisten N, et al. The aerobic capacity – fatigue relationship in persons with Multiple Sclerosis is not reproducible in a pooled analysis of two randomized controlled trials. *Mult Scler Relat Dis* 2022; 58: 103476.

[788] Bjelland I, Dahl AA, Haug TT, et al. The validity of the Hospital Anxiety and Depression Scale An updated literature review. *J Psychosom Res* 2002; 52: 69–77.

[789] Krupp LB, LaRocca NG, Muir-Nash J, et al. The Fatigue Severity Scale: Application to Patients With Multiple Sclerosis and Systemic Lupus Erythematosus. *Arch Neurol-chicago* 1989; 46: 1121–1123.

[790] Bevens W, Weiland TJ, Gray K, et al. The Feasibility of a Web-Based Educational Lifestyle Program for People With Multiple Sclerosis: A Randomized Controlled Trial. *Frontiers Public Heal* 2022; 10: 852214.

[791] Blikman LJ, Meeteren J van, Twisk JW, et al. Effectiveness of energy conservation management on fatigue and participation in multiple sclerosis: A randomized controlled trial. *Mult Scler J* 2017; 23: 1527–1541.

[792] Carletto S, Tesio V, Borghi M, et al. The Effectiveness of a Body-Affective Mindfulness Intervention for Multiple Sclerosis Patients with Depressive Symptoms: A Randomized Controlled Clinical Trial. *Front Psychol* 2017; 8: 2083.

[793] Escudero-Uribe S, Hochsprung A, Heredia-Camacho B, et al. Effect of Training Exercises Incorporating Mechanical Devices on Fatigue and Gait Pattern in Persons with Relapsing-Remitting Multiple Sclerosis. *Physiother Can* 2017; 69: 292–302.

[794] Fitzgerald KC, Morris B, Soroosh A, et al. Pilot randomized active-placebo-controlled trial of low-dose ketamine for the treatment of multiple sclerosis–related fatigue. *Mult Scler J* 2020; 27: 942–953.

[795] Huang S-C, Guerrieri S, Costa GD, et al. Intensive Neurorehabilitation and Gait Improvement in Progressive Multiple Sclerosis: Clinical, Kinematic and Electromyographic Analysis. *Brain Sci* 2022; 12: 258.

[796] Khadadah S, Kimoff RJ, Duquette P, et al. Effect of continuous positive airway pressure treatment of obstructive sleep apnea-hypopnea in multiple sclerosis: A randomized, double-blind, placebo-controlled trial (SAMS-PAP study). *Multiple Scler Houndmills Basingstoke Engl* 2022; 28: 82–92.

[797] Kratz AL, Atalla M, Whibley D, et al. Calling Out MS Fatigue: Feasibility and Preliminary Effects of a Pilot Randomized Telephone-Delivered Exercise Intervention for Multiple Sclerosis Fatigue. *J Neurol Phys Ther* 2020; 44: 23–31.

[798] Flamme ACL, Abernethy D, Sim D, et al. Safety and acceptability of clozapine and risperidone in progressive multiple sclerosis: a phase I, randomised, blinded, placebo-controlled trial. *Bmj Neurology Open* 2020; 2: e000060.

[799] Ozkul C, Guclu-Gunduz A, Irkec C, et al. Effect of combined exercise training on serum brain-derived neurotrophic factor, suppressors of cytokine signaling 1 and 3 in patients with multiple sclerosis. *J Neuroimmunol* 2018; 316: 121–129.

[800] Shangyan H, Kuiqing L, Yumin X, et al. Meta-analysis of the efficacy of modafinil versus placebo in the treatment of multiple sclerosis fatigue. *Mult Scler Relat Dis* 2018; 19: 85–89.

[801] Akker LE van den, Beckerman H, Collette EH, et al. Cognitive behavioral therapy positively affects fatigue in patients with multiple sclerosis: Results of a randomized controlled trial. *Mult Scler J* 2017; 23: 1542–1553.

[802] Vermöhlen V, Schiller P, Schickendantz S, et al. Hippotherapy for patients with multiple sclerosis: A multicenter randomized controlled trial (MS-HIPPO). *Mult Scler J* 2017; 24: 1375–1382.

[803] Yazgan YZ, Tarakci E, Tarakci D, et al. Comparison of the effects of two different exergaming systems on balance, functionality, fatigue, and quality of life in people with multiple sclerosis: A randomized controlled trial. *Mult Scler Relat Dis* 2020; 39: 101902.

[804] Charvet LE, Dobbs B, Shaw MT, et al. Remotely supervised transcranial direct current stimulation for the treatment of fatigue in multiple sclerosis: Results from a randomized, sham-controlled trial. *Mult Scler J* 2017; 24: 1760–1769.

[805] Webster GA, Sim DA, Flamme ACL, et al. Evaluation of neurological changes in secondary progressive multiple sclerosis patients treated with immune modulator MIS416: results from a feasibility study. *Pilot Feasibility Stud* 2017; 3: 60.

[806] Abbadessa G, Lavorgna L, Miele G, et al. Assessment of Multiple Sclerosis Disability Progression Using a Wearable Biosensor: A Pilot Study. *J Clin Medicine* 2021; 10: 1160.

[807] Cehelyk EK, Harvey DY, Grubb ML, et al. Uncovering the association between fatigue and fatigability in multiple sclerosis using cognitive control. *Mult Scler Relat Dis* 2019; 27: 269–275.

[808] Guger M, Ackerl MM, Heine M, et al. Favorable benefit–risk ratio with teriflunomide treatment in relapsing-remitting multiple sclerosis: Results of the 2-year, multicenter, prospective, noninterventional TAURUS MS study in Austria. *Eneurologicalsci* 2022; 27: 100396.

[809] Kallmann BA, Tiel-Wilck K, Kullmann JS, et al. Real-life outcomes of teriflunomide treatment in patients with relapsing multiple sclerosis: TAURUS-MS observational study. *Ther Adv Neurol Diso* 2019; 12: 1756286419835077.

[810] Simpson‐Yap S, Nag N, Probst Y, et al. Higher‐quality diet and non‐consumption of meat are associated with less self‐determined disability progression in people with multiple sclerosis: A longitudinal cohort study. *Eur J Neurol* 2022; 29: 225–236.

[811] Khazaei M, Karevan A, Taheri M, et al. Comparison of the effects of amantadine and ondansetron in treatment of fatigue in patients with multiple sclerosis. *Clin Transl Medicine* 2019; 8: 20.

[812] Pompa A, Morone G, Iosa M, et al. Does robot-assisted gait training improve ambulation in highly disabled multiple sclerosis people? A pilot randomized control trial. *Mult Scler J* 2016; 23: 696–703.

[813] Sadeghi-Naini M, Esslami GG, Fayyazi S, et al. Low dose aspirin for MS-related fatigue: Results of a pilot, double-blind, randomized trial. *Neurology Psychiatry Brain Res* 2017; 25: 24–30.

[814] Carling A, Forsberg A, Gunnarsson M, et al. CoDuSe group exercise programme improves balance and reduces falls in people with multiple sclerosis: A multi-centre, randomized, controlled pilot study. *Multiple Scler Houndmills Basingstoke Engl* 2017; 23: 1394–1404.

[815] Zuber P, Tsagkas C, Papadopoulou A, et al. Efficacy of inpatient personalized multidisciplinary rehabilitation in multiple sclerosis: behavioural and functional imaging results. *J Neurol* 2020; 267: 1744–1753.

[816] Backus D, Moldavskiy M, Sweatman WM. Effects of Functional Electrical Stimulation Cycling on Fatigue and Quality of Life in People with Multiple Sclerosis Who Are Nonambulatory. *Int J Ms Care* 2020; 22: 193–200.

[817] Hestvik ALK, Frederiksen JL, Nielsen HH, et al. Real-world study of relapsing-remitting multiple sclerosis patients treated with Teriflunomide in Nordic countries: Quality-Of-Life, efficacy, safety and adherence outcomes. *Mult Scler Relat Dis* 2022; 63: 103892.

[818] Penner I, Raselli C, Stöcklin M, et al. The Fatigue Scale for Motor and Cognitive Functions (FSMC): validation of a new instrument to assess multiple sclerosis-related fatigue. *Mult Scler J* 2009; 15: 1509–1517.

[819] Arntzen EC, Straume B, Odeh F, et al. Group‐based, individualized, comprehensive core stability and balance intervention provides immediate and long‐term improvements in walking in individuals with multiple sclerosis: A randomized controlled trial. *Physiotherapy Res Int* 2020; 25: e1798.

[820] Cohen ET, Huser S, Barone K, et al. Trekking Poles to Aid Multiple Sclerosis Walking Impairment. *Int J Ms Care* 2021; 23: 135–141.

[821] Hayes S, Uszynski MK, Motl RW, et al. Randomised controlled pilot trial of an exercise plus behaviour change intervention in people with multiple sclerosis: the Step it Up study. *Bmj Open* 2017; 7: e016336.

[822] Kahraman T, Ozdogar AT, Yigit P, et al. Feasibility of a 6-Month Yoga Program to Improve the Physical and Psychosocial Status of Persons with Multiple Sclerosis and their Family Members. *Explor* 2018; 14: 36–43.

[823] KOCICA J, KOLCAVA J, SLADECKOVA M, et al. INTENSIVE CIRCUIT CLASS THERAPY IN PATIENTS WITH RELAPSING-REMITTING MULTIPLE SCLEROSIS. *J Rehabil Med* 2022; 54: 2027.

[824] Molhemi F, Monjezi S, Mehravar M, et al. Effects of Virtual Reality vs Conventional Balance Training on Balance and Falls in People With Multiple Sclerosis: A Randomized Controlled Trial. *Arch Phys Med Rehab* 2021; 102: 290–299.

[825] Novotna K, Rusz J, Havrdova EK, et al. Why patients with multiple sclerosis perceive improvement of gait during treatment with natalizumab? *J Neural Transmission* 2019; 126: 731–737.

[826] Sandroff BM, Bollaert RE, Pilutti LA, et al. Multimodal exercise training in multiple sclerosis: A randomized controlled trial in persons with substantial mobility disability. *Contemp Clin Trials* 2017; 61: 39–47.

[827] Tavazzi E, Bergsland N, Cattaneo D, et al. Effects of motor rehabilitation on mobility and brain plasticity in multiple sclerosis: a structural and functional MRI study. *J Neurol* 2018; 265: 1393–1401.

[828] Conroy SS, Zhan M, Culpepper WJ, et al. Self-directed exercise in multiple sclerosis: Evaluation of a home automated tele-management system. *J Telemed Telecare* 2017; 24: 410–419.

[829] Novotna K, Janatova M, Hana K, et al. Biofeedback Based Home Balance Training can Improve Balance but Not Gait in People with Multiple Sclerosis. *Multiple Scler Int* 2019; 2019: 2854130.

[830] Bethoux F, Varsanik JS, Chevalier TW, et al. Walking speed measurement with an Ambient Measurement System (AMS) in patients with multiple sclerosis and walking impairment. *Gait Posture* 2018; 61: 393–397.

[831] Mamoei S, Jensen HB, Dalgas U, et al. A cross-sectional comparison of performance, neurophysiological and MRI outcomes of responders and non-responders to fampridine treatment in multiple sclerosis – An explorative study. *J Clin Neurosci* 2020; 82: 179–185.

[832] Spain R, Powers K, Murchison C, et al. Lipoic acid in secondary progressive MS. *Neurology Neuroimmunol Neuroinflammation* 2017; 4: NA;

[833] Hobart JC, Riazi A, Lamping DL, et al. Measuring the impact of MS on walking ability: the 12-Item MS Walking Scale (MSWS-12). *Neurology* 2003; 60: 31–6.

[834] Atkinson MJ, Sinha A, Hass SL, et al. Health and Quality of Life Outcomes. *Health and Quality of Life Outcomes* 2004; 2: 12–13.

[835] Coyle PK, Khatri B, Edwards KR, et al. Teriflunomide real-world evidence: Global differences in the phase 4 Teri-PRO study. *Mult Scler Relat Dis* 2019; 31: 157–164.

[836] Schultz TJ, Thomas A, Georgiou P, et al. Home infusions of natalizumab for people with multiple sclerosis: a pilot randomised crossover trial. *Ann Clin Transl Neur* 2021; 8: 1610–1621.

[837] Cutter G, Veneziano A, Grinspan A, et al. Satisfaction and adherence with glatiramer acetate 40mg/mL TIW in RRMS after 12 months, and the effect of switching from 20mg/mL QD. *Mult Scler Relat Dis* 2020; 40: 101957.

[838] Foley JF, Nair KV, Vollmer T, et al. Long-term natalizumab treatment is associated with sustained improvements in quality of life in patients with multiple sclerosis. *Patient Prefer Adher* 2017; 11: 1035–1048.

[839] Hoffmann FA, Trenova A, Llaneza MA, et al. Patient satisfaction with ExtaviPro^TM^ 30G, a new auto-injector for administering interferon β-1b in multiple sclerosis: results from a real-world, observational EXCHANGE study. *Bmc Neurol* 2017; 17: 156.

[840] Fernández O, Duran E, Ayuso T, et al. Treatment satisfaction with injectable disease-modifying therapies in patients with relapsing-remitting multiple sclerosis (the STICK study). *Plos One* 2017; 12: e0185766.

[841] Hendin B, Naismith RT, Wray SE, et al. Treatment satisfaction significantly improves in patients with multiple sclerosis switching from interferon beta therapy to peginterferon beta-1a every 2 weeks. *Patient Prefer Adher* 2018; 12: 1289–1297.

[842] Manchon E, Laplaud D, Vukusic S, et al. Efficacy, safety and patient reported outcomes in patients with active relapsing multiple sclerosis treated with ocrelizumab: Final results from the PRO-MSACTIVE study. *Mult Scler Relat Dis* 2022; 68: 104109.

[843] Repovic P, Robertson D, Kresa-Reahl K, et al. Effectiveness of Dimethyl Fumarate in Patients With Relapsing Multiple Sclerosis Switching After Suboptimal Response to Glatiramer Acetate, Including Patients With Early Multiple Sclerosis: Subgroup Analysis of RESPOND. *Neurology Ther* 2021; 10: 169–182.

[844] Schreiber K, Kant M, Pfleger C, et al. High treatment adherence, satisfaction, motivation, and health-related quality of life with fingolimod in patients with relapsing-remitting multiple sclerosis – results from a 24-month, multicenter, open-label Danish study. *Patient Prefer Adher* 2018; 12: 1139–1150.

[845] Turčáni P, Mašková J, Húska J. Real-World Treatment Patterns of Disease Modifying Therapy (DMT) for Patients with Relapse-Remitting Multiple Sclerosis and Patient Satisfaction with Therapy: Results of the Non-Interventional SKARLET Study in Slovakia. *Patient Prefer Adher* 2020; 14: 1129–1135.

[846] Menge T, Rehberg-Weber K, Taipale K, et al. Peginterferon beta-1a was associated with high adherence and satisfaction in patients with multiple sclerosis in a German real-world study. *Ther Adv Neurol Diso* 2021; 14: 17562864211000460.

[847] Goodin DS, Reder AT, Traboulsee AL, et al. Predictive validity of NEDA in the 16- and 21-year follow-up from the pivotal trial of interferon beta-1b. *Multiple Scler Houndmills Basingstoke Engl* 2018; 25: 837–847.

[848] Horakova D, Uher T, Krasensky J, et al. Long-term effectiveness of natalizumab on MRI outcomes and no evidence of disease activity in relapsing-remitting multiple sclerosis patients treated in a Czech Republic real-world setting: A longitudinal, retrospective study. *Mult Scler Relat Dis* 2020; 46: 102543.

[849] Pietrzak A, Kalinowska-Łyszczarz A, Kozubski W, et al. Evaluation of clinical prognostic factors in Polish interferon beta-1b treated multiple sclerosis patients. *Neurol Neurochir Pol* 2019; 53: 458–465.

[850] Lattanzi S, Rocchi C, Danni M, et al. Long-term outcome in multiple sclerosis patients treated with fingolimod. *Mult Scler Relat Dis* 2020; 45: 102416.

[851] Prosperini L, Ruggieri S, Haggiag S, et al. Prognostic Accuracy of NEDA-3 in Long-term Outcomes of Multiple Sclerosis. *Neurology - Neuroimmunol Neuroinflammation* 2021; 8: e1059.

[852] Walo-Delgado PE, Maza SS de la, Villarrubia N, et al. Low serum neurofilament light chain values identify optimal responders to dimethyl fumarate in multiple sclerosis treatment. *Sci Rep-uk* 2021; 11: 9299.

[853] Kvistad SAS, Burman J, Lehmann AK, et al. Impact of previous disease-modifying treatment on safety and efficacy in patients with MS treated with AHSCT. *J Neurology Neurosurg Psychiatry* 2022; 93: 844–848.

[854] Koch MW, Mostert J, Repovic P, et al. Early first‐line treatment response and subsequent disability worsening in relapsing–remitting multiple sclerosis. *Eur J Neurol* 2022; 29: 1106–1116.

[855] Rotstein D, Solomon JM, Sormani MP, et al. Association of No Evidence of Disease Activity With No Long-term Disability Progression in Multiple Sclerosis. *Neurology* 2022; 99: e209–e220.

[856] Harris S, Comi G, Cree BAC, et al. Plasma neurofilament light chain concentrations as a biomarker of clinical and radiologic outcomes in relapsing multiple sclerosis: Post hoc analysis of Phase 3 ozanimod trials. *Eur J Neurol* 2021; 28: 3722–3730.

[857] Kappos L, Havrdová E, Giovannoni G, et al. No evidence of disease activity in patients receiving daclizumab versus intramuscular interferon beta-1a for relapsing-remitting multiple sclerosis in the DECIDE study. *Mult Scler J* 2016; 259: 1352458516683266.

[858] Giovannoni G, Singer BA, Issard D, et al. Durability of no evidence of disease activity-3 (NEDA-3) in patients receiving cladribine tablets: The CLARITY extension study. *Mult Scler J* 2021; 28: 1219–1228.

[859] Guerra T, Caputo F, Orlando B, et al. Long-term comparative analysis of no evidence of disease activity (NEDA-3) status between multiple sclerosis patients treated with natalizumab and fingolimod for up to 4 years. *Neurol Sci* 2021; 42: 4647–4655.

[860] Ozakbas S, Cinar BP, Baba C, et al. Self‐injectable DMTs in relapsing MS: NEDA assessment at 10 years in a real‐world cohort. *Acta Neurol Scand* 2022; 145: 557–564.

[861] Guevara C, Garrido C, Martinez M, et al. Prospective Assessment of No Evidence of Disease Activity-4 Status in Early Disease Stages of Multiple Sclerosis in Routine Clinical Practice. *Front Neurol* 2019; 10: 788.

[862] Mansilla MJ, Navarro‐Barriuso J, Presas‐Rodríguez S, et al. Optimal response to dimethyl fumarate is mediated by a reduction of Th1‐like Th17 cells after 3 months of treatment. *Cns Neurosci Ther* 2019; 25: 995–1005.

[863] Margoni M, Rinaldi F, Riccardi A, et al. No evidence of disease activity including cognition (NEDA-3 plus) in naïve pediatric multiple sclerosis patients treated with natalizumab. *J Neurol* 2020; 267: 100–105.

[864] Ioia M di, Stefano VD, Farina D, et al. Alemtuzumab treatment of multiple sclerosis in real-world clinical practice: A report from a single Italian center. *Mult Scler Relat Dis* 2020; 38: 101504.

[865] Alcalá C, Gascón F, Pérez-Miralles F, et al. Treatment with alemtuzumab or rituximab after fingolimod withdrawal in relapsing–remitting multiple sclerosis is effective and safe. *J Neurol* 2019; 266: 726–734.

[866] Piervincenzi C, Petsas N, Giglio LD, et al. Increased Within-Network Functional Connectivity May Predict NEDA Status in Fingolimod-Treated MS Patients. *Front Neurol* 2021; 12: 632917.

[867] Masanneck L, Rolfes L, Regner-Nelke L, et al. Detecting ongoing disease activity in mildly affected multiple sclerosis patients under first-line therapies. *Mult Scler Relat Dis* 2022; 63: 103927.

[868] Kvistad SAS, Lehmann AK, Trovik LH, et al. Safety and efficacy of autologous hematopoietic stem cell transplantation for multiple sclerosis in Norway. *Mult Scler J* 2019; 26: 1889–1897.

[869] Herman JA, Khalighinejad F, York K, et al. A real-world cohort analysis of alemtuzumab outcomes in relapsing multiple sclerosis. *Mult Scler Relat Dis* 2021; 47: 102619.

[870] Menascu S, Fattal-Valevski A, Vaknin-Dembinsky A, et al. Effect of natalizumab treatment on the rate of No Evidence of Disease Activity in young adults with multiple sclerosis in relation to pubertal stage. *J Neurol Sci* 2022; 432: 120074.

[871] Havrdová E, Arnold DL, Bar-Or A, et al. No evidence of disease activity (NEDA) analysis by epochs in patients with relapsing multiple sclerosis treated with ocrelizumab vs interferon beta-1a. *Multiple Scler J Exp Transl Clin* 2018; 4: 2055217318760642.

[872] Sormani MP, Bruzzi P, Beckmann K, et al. MRI metrics as surrogate endpoints for EDSS progression in SPMS patients treated with IFN &bgr;-1b. *Neurology* 2003; 60: 1462–1466.

[873] Sormani MP, Bruzzi P, Comi G, et al. MRI metrics as surrogate markers for clinical relapse rate in relapsing-remitting MS patients. *Neurology* 2002; 58: 417–421.

[874] Eijlers AJC, Geest Q van, Dekker I, et al. Predicting cognitive decline in multiple sclerosis: a 5-year follow-up study. *Brain* 2018; 141: 2605–2618.

[875] Portaccio E, Goretti B, Zipoli V, et al. Reliability, practice effects, and change indices for Rao’s brief repeatable battery. *Mult Scler J* 2010; 16: 611–617.
